# Organocatalytic Enantioselective Conjugate Addition of Nitromethane to Benzylidene-2-Benzoyl Acetate: Asymmetric Synthesis of ABT – 627, an Endothelin Receptor Antagonist

**DOI:** 10.3389/fchem.2020.00135

**Published:** 2020-03-05

**Authors:** Saumen Hajra, Sk Mohammad Aziz, Bibekananda Jana, Sunit Hazra

**Affiliations:** ^1^Centre of Biomedical Research, Sanjay Gandhi Post-Graduate Institute of Medical Sciences, Lucknow, India; ^2^Department of Chemistry, Indian Institute of Technology Kharagpur, Kharagpur, India

**Keywords:** nitromethane, conjugate addition, organocatalytic, asymmetric synthesis, ABT – 627

## Abstract

First catalytic and enantioselective conjugate addition of nitromethane to benzylidene-2-benzoyl acetate has been developed using dihydroquinine derived squaramide catalyst with moderate to high selectivities. Asymmetric total synthesis of ABT-627, a potent ETA receptor antagonist is accomplished utilizing the developed method in overall 15.7% yield.

**Graphical Abstract:**
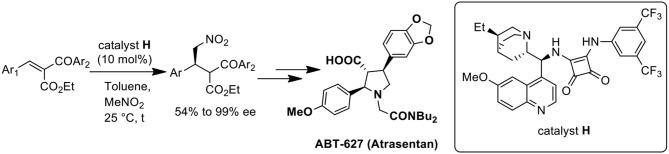
Catalytic enantioselective conjugate addition of nitromethane for the synthesis of ABT-627.

## Introduction

**Figure 1:**
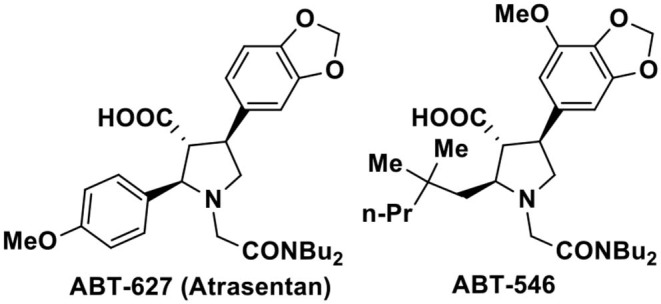
Representative of biologically active 2,4-di-substituted-pyrrolidine-3-carboxylic acids.

The nitro compounds due to their easy transformations into other diverse functional groups, role as valuable reagents as well as their presence in many natural products occupy a distinct position in organic synthesis (Ono, 2001). Particularly, synthesis of nitrogen containing heterocyclic compounds such as pyrroles, indoles, pyrrolidines, and their derivatives have often been achieved via the intermediacy of suitable nitro compounds. Among the five-membered nitrogen heterocycles, the pyrrolidine-3-carboxylic acid and its aryl substituted analogs are privileged structural motifs present in a wide range of biologically active therapeutic agents (Pandey et al., 2006; Royer, 2009). Specially, the 2,4-disubstituted pyrrolidine-3-carboxylic acids are of particular interest owing to their preclinical and clinical activities toward endothelin-A receptor antagonists such as ABT 627 and ABT 546 (Figure 1; Bhagwat et al., 1996; Winn et al., 1996; Tasker et al., 1997; Liu et al., 1998; Wu-Wong et al., 1999; Jae et al., 2001; Young et al., 2007; He et al., 2010). The important biological profile coupled with the *trans-trans* stereo disposition of the -CO_2_H group with adjacent substituents have generated substantial synthetic challenges to organic chemists for the asymmetric synthesis of these 2,4-disubstituted pyrrolidine-3-carboxylic acids.

**Scheme 1:**
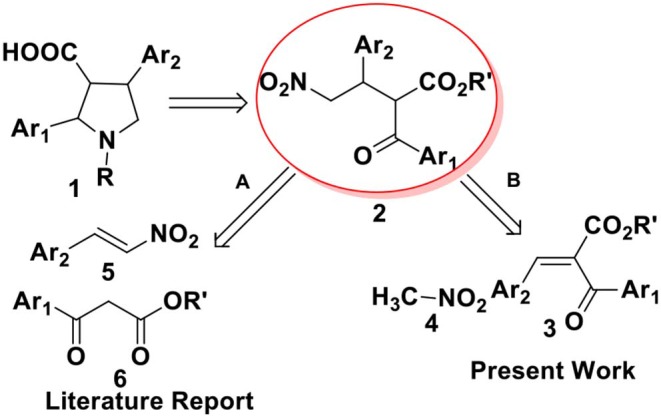
Retrosynthetic routes toward synthesis of 2,4-diarylpyrrolidine-3-carboxylic acid 1.

From a retrosynthetic view point, 2,4-diaryl pyrrolidine-3-carboxylic acids **1** can be obtained from α-benzoyl-β-aryl-γ-nitro butyric acid derivative **2**, which can be synthesized either by Michael addition of 1,3-dicarbonyl compounds to nitrostyrenes (route A, Scheme 1) or by nitromethane addition to benzylidene-2-benzoyl acetate (route B, Scheme 1). Till date, there are several reports on metal (Ji et al., 1999; Barnes et al., 2002; Evans and Seidel, 2005; Evans et al., 2007) or organo-catalyzed (Okino et al., 2005; Malerich et al., 2008; Han et al., 2009; Jiang et al., 2009; Dong et al., 2012; Chen et al., 2013; Jia et al., 2013) asymmetric conjugate addition of malonates to nitroolefins. However, the addition of ethyl benzoyl acetate or its derivatives to nitro styrene, particularly toward the synthesis of ABT-627 and ABT-546 are not explored much. In 2002, Barnes and co-workers reported an efficient bis(oxazoline)-Mg(OTf)_2_ complex mediated addition of ketoesters to nitroolefins for the synthesis of ABT-546 with 88% selectivity and the adduct for ABT-627 with 70% selectivity (Barnes et al., 2002). The second strategy to synthesize α-acyl-β-aryl-γ-amino butyric acid esters **2** includes the addition of nitromethane to benzylidene-2-benzoyl acetate **3**. We (Hajra et al., 2013) and others (Ooi et al., 2004; Chiarucci et al., 2012) reported the asymmetric conjugate addition of nitroalkane to alkylidenemalonates with good to excellent enantioselectivity. However, the addition of nitromethane to benzylidene benzoyl acetate **3** toward the synthesis of 2,4-disubstituted pyrrolidine-3-carboxylic acid derivatives **1** has not been investigated in details. Herein, we report an organocatalytic asymmetric conjugate addition of nitromethane to benzylidene-2-benzoyl acetate for the synthesis of α-benzoyl-β-aryl-γ-nitro butyric acid derivatives and its application for the total synthesis of ABT-627, an endothelin receptor antagonist.

## Results and Discussion

Our previous report (Hajra et al., 2013) on enantioselective nitromethane addition to alkylidenemalonates catalyzed by cinchona-alkaloid derived thiourea based organocatalyst offered a simple but efficient route to the non-racemic synthesis of 3-substituted pyrrolidine/pyrrolidone class of compounds. The regular occurrence of aryl groups in many pyrrolidine/pyrrolidone based natural products provoked us to further extend the protocol to the enantioselective conjugate addition of nitromethane to benzyledine-2-benzoyl acetate.

In recent decade, the library of chiral bifunctional hydrogen bond donor organocatalysts have been enriched by the pioneering discoveries of Jacobsen (Yoon and Jacobsen, 2005), Takemoto (Okino et al., 2005), Rawal (Malerich et al., 2008), Dixon (Núñez et al., 2013), and others (Vakulya et al., 2005; Yang and Du, 2010). These vast arrays of catalysts along with their unique activation mode toward nitroalkanes allured us to identify the effective organocatalyst for our reaction.

**Scheme 2:**
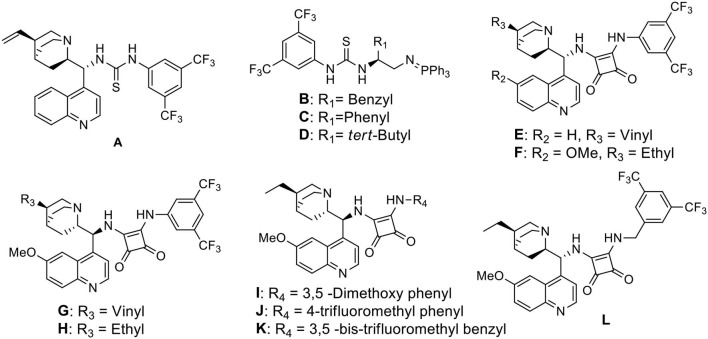
Bifunctional organocatalysts selected for the optimization study.

We started our investigation by studying the nitromethane addition to substrate **3a** using various organocatalysts (Scheme 2, Table 1). To our delight, the initial experiment with catalyst **A** gave good yield (71%) of the product with encouraging enantioselectivity (47% ee, of major diastereisomer) (Table 1, entry 1). Then effect of increasing basicity of the catalysts was checked using bifunctional iminophosphorane organocatalysts. The chiral amino acids derived catalysts **B-D** gave better yields (82, 80, and 85%, respectively), but the diastereo- and enantioselectivities were poor (entries 2–4). We also examined some chiral squaramide based hydrogen bonding organocatalysts **E-L** (entries 5–12).

**Table 1:** Catalyst screening study^a^.

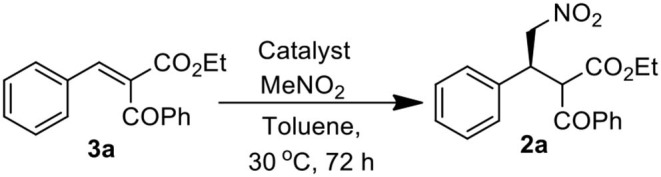				
**Entry**	**catalyst**	**Yield^b^ (%)**	**dr**	**ee^c^ (major)(%)**
1	A	71	4:1	47
2	B	82	1:1	25
3	C	80	1:1	33
4	D	85	1.5:1	40
5	E	61	4:1	58
6	F	72	1.9:1	75
7	G	72	1.2:1	(**–**)64
**8**	**H**	**74**	**4:1**	**(–)81**
9	I	56	1.2:1	(**–**)54
10	J	64	1.2:1	(**–**)58
11	K	64	1.5:1	(**–**)33
12	L	62	2.3:1	58

a Reaction conditions: **3a** (0.40 mmol), catalyst (10 mol%) in nitromethane (0.4 ml, 20 equiv.) and toluene (0.8 ml) was stirred at room temperature (30°C).

b *Isolated yields after column chromatography; 10–20% substrate was recovered*.

c *Enantioselectivity was determined by HPLC using chiral column*.

*Bold values indicate the optimized reaction conditions*.

The cinchonine derived squaramide catalyst **E** gave 61% yield of the product with better dr (4:1) and slightly higher ee (58%) (entry 5) whereas a quinine derived squaramide catalyst **G** further improved the yield (72%) and enantioselectivity (64%) with reverse asymmetric induction (entry 7) but the diastereoselectivity was not good (dr 1.2:1). On the other hand, the dihydroquinidine derived catalyst **F** afforded similar yield (72%) of the product with a bit higher selectivity (dr 1.9:1, ee 75%) (entry 6) and its pseudoenantiomeric catalyst **H** afforded the best result among the catalysts screened with 74% yield and 81% enantioselectivity in favor of the major isomer (dr 4:1) (entry 8). Variation in aryl moieties of the squaramide motifs (catalysts **I-L**), however, were found to be inferior to catalyst **H** on the stereochemical outcome of the reaction (entries 9–12).

With the optimized catalyst **H**, we then turned our attention to find the suitable solvent for the reaction and thus a solvent screening study was performed (Table 2). The study revealed the effect of different solvents on the diastereoselectivity and enantioselectivity of the reaction. We found toluene to be the best solvent of choice in terms of overall yield (74%), diastereoselectivity (dr = 4:1) and enantioselectivity [ee = 81/69 (major/ minor)] of the desired product **2a** (entry 6, Table 2).

**Table 2:** Solvent screening with optimized catalyst **H**^a^.

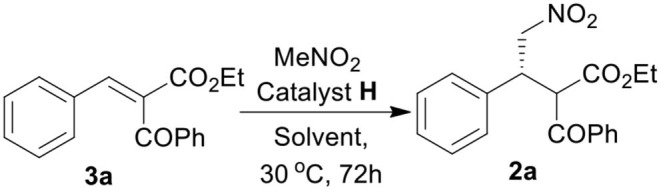				
	**Solvent**	**Yield^b^ (%)**	**ee^c^ (major/minor)**	**dr**
1	Neat	67	72/63	1:1
2	MTBE	66	68/52	1.5:1
3	THF	57	63/54	1.5:1
4	DCE	65	72/72	1.5:1
5	Chloroform	64	55/70	3:1
6	Toluene	74	81/69	4:1

a Reaction conditions: **3a** (0.357 mmol), catalyst (10 mol%) in nitromethane (0.4 ml, 20 equiv), and solvent (0.7 ml) was stirred at room temperature (30°C).

b *Isolated yields after column chromatography; 10–20% substrate **3a** was recovered*.

c *Enantioselectivity was determined by HPLC using chiral column*.

**Scheme 3:**
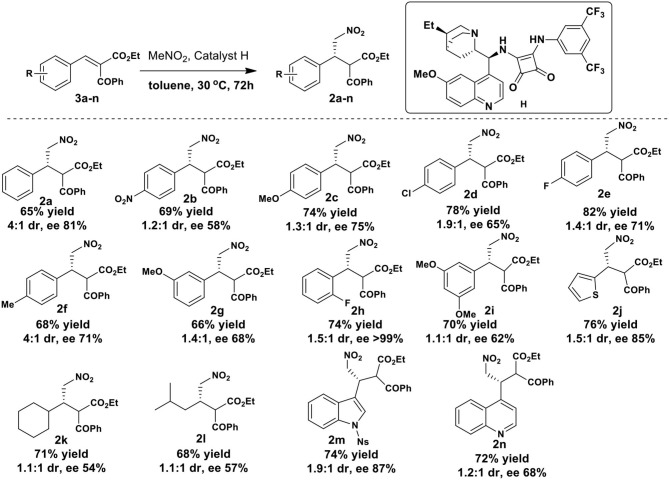
Scope of nitromethane addition to benzylidine benzoyl acetates **3a-n**.

Having optimized the reaction conditions, the applicability of current method was then checked with various aromatic and heterocyclic benzylidene-2-benzoyl acetate substrates (Scheme 3; NMR spectra and HPLC chromatograms of all new compounds are disclosed in the Supplementary Material). All the substrates **3a-n** investigated underwent smooth reaction to provide the corresponding products **2a-n** in good yields with moderate diastereoselectivity. In general, yields were higher with substituted aromatic groups on substrates. The *para*-substituent on benzene ring lowered the diaseteroselectivity (**2b-e**) except for *p*-methyl substrate where the diaseteroselectivity was good (**2f**, 4:1). Substrates with electron donating group at *para*-position showed slightly higher enantioselectivity compared to those with electron withdrawing groups. Substrate with a methoxy group at *meta*-position showed moderate selectivity (**2g**, dr 1.4:1, ee 68%). The best enantioselection was observed in case of *ortho*-fluoro substrate (**2h**, ee>99%) with moderate diastereoselectivity (dr 1.5:1). The 3,5-dimethoxy (**2i**, dr 1.1:1, ee 62%) and quinoline (**2n**, dr.1.2:1, ee 68%) derived substrates showed comparable results. Moderate dr with good enantioselectivity of the addition product was obtained in case of 2-thienyl substrate (**2j**, dr 1.5:1, ee 85%) and *N*-nosyl-3-indolyl substrate (**2m**, dr 1.9:1, ee 87%). Both dr and ee were found to be low for aliphatic 2-benzoyl acetate substrates **2k** (dr 1.1:1, ee 54%) and **2l** (dr 1.1:1, ee 57%). The stereochemistry of the products **2** was assigned based on the analogy of our earlier work (Hajra et al., 2013).

**Scheme 4:**
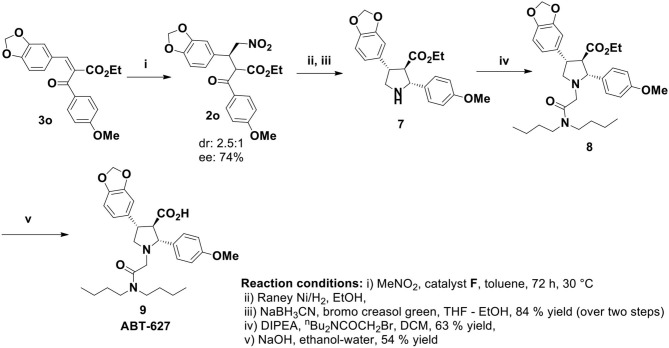
Total synthesis of ABT-627.

The nitromethane addition product **2** could be the key precursor for the synthesis of 2,4-diaryl-pyrrolidine-3-carboxylic acids or esters by common synthetic transformation. One of these classes of compounds is atrasentan (ABT 627), a potent selective antagonist of endothelin-A (ETA) receptor, inhibits angiogenesis and tumor cell proliferation by down regulating the endothelin-A (ETA) receptor signaling. Previously the total synthesis of ABT-627 had been achieved by employing chiral auxiliary mediated acylation (Winn et al., 1996), aldol reaction (Wittenberger and McLaughlin, 1999), and hetero Diels-Alder reaction (Buchholz and Reißig, 2003). Our journey for the catalytic enantioselective synthesis of ABT-627 began with the 1.0 mmol scale reaction of **3o** with nitromethane under optimized condition. Catalyst **F** was used in this reaction for stereochemical requirement of the addition product (Scheme 4). Raney nickel mediated hydrogenation of the nitromethane addition product **2o** produced intermediated imine compound, which was then subjected to sodium cyanoborohydride reduction to yield the pyrrolidine **7** in 84% yield over two steps (crude reaction mixture showed 2.6:1 dr, major product separated by column chromatography to obtain 56% of desired diastereoisomer of **7**). Pyrrolidine **7** was then alkylated to get compound **8** (yield 63%). Finally, the synthesis of ABT-627 was completed by saponification of **8** in ethanol/water with overall 15.7% yield and 67% enantioselectivity.

## Conclusion

In conclusion, we disclose the first catalytic and enantioselective conjugate addition of nitromethane to benzylidene-2-benzoyl acetate. Dihydroquinine derived squaramide catalyst was found to render good yields with moderate to excellent selectivities. The methodology was further elaborated by transformation of the appropriate addition product to a potent ETA receptor antagonist ABT-627 in overall 15.7% yield with moderate enantioselectivity.

## Materials and Methods

### Experimental Section

#### General Information

All the reactions were carried out using oven dried glass wares under an atmosphere of Argon (Ar). Commercially available reagents were used without further purification. Solvents were dried and distilled following standard procedure. Flash column chromatography was performed in all the cases using silica gel (230–400 mesh) purchased from SRL India. Analytical TLC was performed on aluminum-backed plates coated with silica gel 60 with F_254_ indicator and compounds were visualized by irradiation of UV light. The ^1^H NMR and ^13^C NMR spectra were measured with Bruker-200 (200 MHz) and Bruker-400 (400 MHz) using CDCl_3_.^1^H NMR chemicals shift are expressed in ppm(δ) relative to CDCl_3_ (δ = 7.26) and ^13^C NMR chemical shift are expressed in ppm(δ) relative to CDCl_3_ resonance (δ = 77.00). Electron spray ionization (ESI) mass spectrometry (MS) experiment were performed on Agilent Technologies 6530 Accurate- Mass Q-TOF LC/MS. High performance liquid chromatography (HPLC) analyses were conducted in CHIRALPAK AD-3 column using two different catalysts (**H** and **F**).

##### General procedure for the synthesis of (3R)-ethyl 2-benzoyl-4-nitro-3-phenylbutanoate (2a)

Under argon atmosphere to a stirred solution of benzylidene-2-benzoyl acetate **3a** (0.112 g, 0.40 mmol) and nitromethane (0.4 mL, 20 equiv) in toluene (0.8 ml) was added organocatalyst **H** (0.022 g, 0.040 mmol) at room temperature (30°C). The resulting mixture was stirred at the same temperature and monitored by TLC. After being stirred for 3 days, the reaction mixture was concentrated in vacuum under rt. The residue was purified by flash column chromatography on silica gel (Hexanes/EtOAc = 9/1) to afford the desired product **2**(0.10 g, 74%).

##### (3R)-ethyl 2-benzoyl-4-nitro-3-phenylbutanoate (2a; Jiang et al., 2009)

The product was prepared by following the general procedure and was obtained as a white solid in 65% yield. ^1^H NMR (400 MHz, Chloroform-*d*) δ 8.07 (d, *J* = 7.6 Hz, 2H), 7.64 (t, *J* = 7.4 Hz, 1H), 7.51 (t, *J* = 7.7 Hz, 2H), 7.37 – 7.28 (m, 5H), 4.96 (d, *J* = 10.0 Hz, 1H), 4.88 – 4.73 (m, 3H), 4.51 (ddd, *J* = 9.9, 8.2, 4.9 Hz, 1H), 3.89 (qd, *J* = 7.1, 2.2 Hz, 2H), 0.91 (t, *J* = 7.1 Hz, 3H) 0.1^3^C NMR (100 MHz, Chloroform-*d*) δ 192.7, 167.0, 136.2, 135.8, 134.3, 128.9, 128.35, 128.29, 78.0, 61.9, 57.0, 43.1, 13.6. HRMS (ESI-TOF) m/z: [M+Na]^+^ Calcd for C_19_H_19_NO_5_ 364.1161; found 364.1151. HPLC analysis**:** *ee* 81% (major diastereoisomer); 25 cm Chiralpak AD-3 column, *n*-hexane/*i*-PrOH = 60/40 (v/v), flow rate: 1 mL/min, 254 nm; *t*_R_(major) = 5.58 min, *t*_R_(minor) = 8.47 min; *ee* 67% (minor diastreoisomer) *t*_*R*_ (major) = 7.6 min, *t*_*R*_ (minor) = 11.44 min.

##### (3R)-ethyl 2-benzoyl-4-nitro-3-(4-nitrophenyl)butanoate (2b)

The product was prepared by following the general procedure and was obtained as a pale yellow solid in 69% yield. ^1^H NMR (400 MHz, Chloroform-*d*) δ 8.23 (d, *J* = 8.3 Hz, 2H), 8.07 (d, *J* = 7.8 Hz, 2H), 7.68 (t, *J* = 7.4 Hz, 1H), 7.56 – 7.51 (m, 4H), 4.98 – 4.94 (m, 1H), 4.88 – 4.81 (m, 3H), 4.68 – 4.61 (m, 1H), 3.93 (q, *J* = 7.1 Hz, 2H), 0.95 (td, *J* = 7.1, 1.8 Hz, 3H). ^13^C NMR (100 MHz, Chloroform-*d*) δ 192.0, 166.5, 147.8, 143.8, 135.4, 134.6, 129.5, 129.0, 128.9, 124.1, 77.3, 62.4, 56.34, 42.7, 13.7. HRMS (ESI-TOF) m/z: [M+Na]^+^ Calcd for C_19_H_18_N_2_O_7_ 409.1012; found 409.0997. HPLC analysis: *ee* 58% (major diastereoisomer); 25 cm Chiralpak AD-3 column, *n*-hexane/*i*-PrOH = 60/40 (v/v), flow rate: 1 mL/min, 254 nm; *t*_R_(major) = 8.96 min, *t*_R_(minor) = 18.95 min; *ee* 56% (minor diastereoisomer); *t*_R_(major) = 11.71 min, *t*_R_(minor) = 21.86 min.

##### (3R)-ethyl 2-benzoyl-3-(4-methoxyphenyl)-4-nitrobutanoate (2c)

The product was prepared by following the general procedure and was obtained as a white solid in 74% yield. ^1^H NMR (400 MHz, Chloroform-*d*) δ 7.87 (d, *J* = 7.4 Hz, 2H), 7.43 (t, *J* = 7.7 Hz, 2H), 7.24 (d, *J* = 8.6 Hz, 2H), 7.15 – 7.12 (d, *J* = 8.6 Hz, 2H), 6.85 (s, 1H), 4.77 – 4.73 (m, 4H), 4.41 (dt, *J* = 9.2, 4.8 Hz, 1H), 4.19 (q, *J* = 7.1 Hz, 2H), 3.79 (s, 3H), 1.19 (t, *J* = 7.1 Hz, 3H). ^13^C NMR (100 MHz, Chloroform-*d*) δ 192.8, 167.8, 159.9, 135.8, 133.8, 129.1, 128.9, 126.0, 128.6, 114.9, 76.9, 62.2, 56.5, 55.3, 42.5, 13.9. HRMS (ESI-TOF) m/z: [M+Na]^+^ Calcd for C_20_H_21_NO_6_ 371.1369 found 371.1351. HPLC analysis**:** *ee* 78% (major diastereoisomer); 25 cm Chiralpak AD-3 column, *n*-hexane/*i*-PrOH = 80/20 (v/v), flow rate: 1 mL/min, 254 nm; *t*_R_(major) = 10.49 min, *t*_R_(minor) = 19.32 min; *ee* 70% (minor diastereoisomer); *t*_R_(major) = 17.14 min, *t*_R_(minor) = 27.15 min.

##### (3*R*)-ethyl 2-benzoyl-3-(4-chlorophenyl)-4-nitrobutanoate (2d; Jiang et al., 2009)

The product was prepared by following the general procedure and was obtained as colorless semisolid in 78% yield. Major Diastereoisomer: ^1^H NMR (200 MHz, Chloroform-*d*) δ 8.11 – 7.98 (m, 2H), 7.56 – 7.44 (m, 3H), 7.32 – 7.23 (m, 4H), 4.96 – 4.83 (m, 3H), 4.49 – 4.38 (m, 1H), 3.90 (q, *J* = 7.1 Hz, 2H), 0.94 (t, *J* = 7.1 Hz, 3H). ^13^C NMR (50 MHz, Chloroform-*d*) δ 192.4, 166.8, 134.8, 134.3, 134.0, 129.7, 129.4, 129.1, 128.92, 128.87, 128.81, 128.5, 77.8, 62.1, 56.8, 42.5, 13.6. Minor Diastereoisomer: ^1^H NMR (200 MHz, Chloroform-*d*) δ 7.91 – 7.81 (m, 1H), 7.67 – 7.56 (m, 2H), 7.18 (d, *J* = 2.6 Hz, 2H), 4.79 – 4.69 (m, 2H), 4.54 – 4.48 (m, 1H), 4.19 (q, *J* = 7.1 Hz, 1H), 1.18 (t, *J* = 7.1 Hz, 2H). ^13^C NMR (50 MHz, Chloroform-*d*) δ 192.3, 167.4, 135.8, 135.6, 135.3, 129.7, 129.4, 128.92, 128.8, 62.4, 56.2, 42.6, 13.9. HRMS (ESI-TOF) m/z: [M+Na]^+^ Calcd for C_19_H_18_ClNO_5_ 375.0874 found 375.0863. HPLC analysis**:** *ee* 65% (major diastereoisomer); 25 cm Chiralpak AD-3 column, *n*-hexane/*i*-PrOH = 60/40 (v/v), flow rate: 1 mL/min, 254 nm; *t*_R_(major) = 6.29 min, *t*_R_(minor) = 10.59 min; *ee* 64% (minor diastereoisomer); *t*_*R*_ (major) = 8.67 min, *t*_*R*_ (minor) = 13.10 min.

##### (3*R*)-ethyl 2-benzoyl-3-(4-fluorophenyl)-4-nitrobutanoate (2e)

The product was prepared by following the general procedure and was obtained as a colorless sticky solid in 82% yield. Major Diastereoisomer: ^1^H NMR (200 MHz, Chloroform-*d*) δ 8.11 – 7.99 (m, 2H), 7.51 – 7.39 (m, 3H), 7.28 (dd, *J* = 6.1, 2.5 Hz, 2H), 7.09 – 6.99 (m, 2H), 4.95 – 4.84 (m, 3H), 4.50 – 4.40 (m, 1H), 3.89 (q, *J* = 7.2 Hz, 2H), 0.93 (t, *J* = 7.1 Hz, 3H). ^13^C NMR (50 MHz, Chloroform-*d*) δ 192.5, 166.8, 135.7, 134.3, 133.9, 130.1, 129.9, 129.8, 129.6, 128.91, 128.86, 128.78, 128.5, 116.1, 115.7, 78.0, 62.0, 57.0, 42.4, 13.6. Minor Diastereoisomer: ^1^H NMR (200 MHz, Chloroform-*d*) δ 7.91 – 7.79 (m, 2H), 7.59 (d, *J* = 3.6 Hz, 1H), 7.45 – 7.38 (m, 2H), 7.22 – 7.12 (m, 2H), 6.91 (t, *J* = 8.6 Hz, 2H), 4.80 – 4.71 (m, 2H), 4.55 – 4.49 (m, 1H), 4.19 (q, *J* = 7.1 Hz, 2H), 1.18 (t, *J* = 7.2 Hz, 2H) 0.1^3^C NMR (50 MHz, Chloroform-*d*) δ 192.5, 167.5, 135.9, 135.7, 134.3, 133.9, 132.5, 131.9, 130.1, 129.9, 129.8, 129.6, 128.9, 128.8, 128.8, 128.5, 116.1, 115.7, 77.9, 62.3, 56.3, 42.4, 13.9.HRMS (ESI-TOF) m/z: [M+Na]^+^ Calcd for C_19_H_18_FNO_5_ 382.1067; found 382.1044. HPLC analysis**:***ee* 70% (major diastereoisomer); 25 cm Chiralpak AD-3 column, *n*-hexane/*i*-PrOH = 80/20 (v/v), flow rate: 1 mL/min, 254 nm; *t*_R_(major) = 8.84 min, *t*_R_(minor) = 16.23 min; *ee* 67% (minor diastereoisomer); *t*_*R*_ (major) = 15.30 min, *t*_*R*_ (minor) = 23.18 min.

##### (3*R*)-ethyl 2-benzoyl-4-nitro-3-(p-tolyl)butanoate (2f; Jiang et al., 2009)

The product was prepared by following the general procedure and was obtained as a colorless gummy liquid in 68% yield. Major Diastereoisomer: ^1^H NMR (400 MHz, Chloroform-*d*) δ 8.08 – 7.99 (m, 2H), 7.65 – 7.58 (m, 1H), 7.49 (t, *J* = 7.8 Hz, 2H), 7.18 (d, *J* = 8.1 Hz, 2H), 7.11 (d, *J* = 8.1 Hz, 2H), 4.94 – 4.88 (m, 2H), 4.81 – 4.76 (m, 1H), 4.48 – 4.39 (m, 1H), 3.88 (qd, *J* = 7.1, 1.8 Hz, 2H), 2.30 (s, 3H), 0.92 (t, *J* = 7.1 Hz, 3H). ^13^C NMR (100 MHz, Chloroform-*d*) δ 193.1, 167.2, 138.3, 134.4, 129.8, 129.9, 129.1, 129.07, 128.3, 128.0, 78.3, 62.1, 57.3, 43.0, 21.3, 13.8. Minor Diastreoisomer:^1^H NMR (400 MHz, Chloroform-*d*) δ 7.89 – 7.84 (m, 1H), 7.58 – 7.52 (m, 1H), 7.42 (t, *J* = 7.7 Hz, 1H), 7.31 – 7.26 (m, 1H), 7.09 (d, *J* = 8.0 Hz, 1H), 7.02 (d, *J* = 7.9 Hz, 1H), 4.94 – 4.88 (m, 2H), 4.41 (ddd, *J* = 14.4, 6.8, 3.7 Hz, 1H), 2.23 (s, 1H), 1.17 (t, *J* = 7.1 Hz, 1H). ^13^C NMR (100 MHz, Chloroform-*d*) δ 192.9, 167.9, 136.1, 133.3, 129.9, 129.9, 128.9, 128.8, 128.3, 127.9, 78.3, 62.4, 56.7, 42.9, 21.2, 14.1. HRMS (ESI-TOF) m/z: [M+Na]^+^ Calcd for C_20_H_21_NO_5_ 378.1317; found 378.1324. HPLC analysis: *ee* 73% (major diastereoisomer); 25 cm Chiralpak AD-3 column, *n*-hexane/*i*-PrOH = 70/30 (v/v), flow rate: 1 mL/min, 254 nm; *t*_R_(major) = 5.71 min, *t*_R_(minor) = 7.64 min; *ee* 73% (minor diastereoisomer) *t*_*R*_ (major) = 6.71 min, *t*_*R*_ (minor) = 8.82 min.

##### (3*R*)-ethyl 2-benzoyl-3-(3-methoxyphenyl)-4-nitrobutanoate (2g)

The product was prepared by following the general procedure and was obtained as a pale yellow semisolid in 66% yield. Major Diastreoisomer: ^1^H NMR (400 MHz, Chloroform-*d*) δ 8.08 – 8.00 (m, 2H), 7.65 – 7.58 (m, 1H), 7.48 (t, *J* = 7.6 Hz, 2H), 7.23 (t, *J* = 7.8 Hz, 1H), 6.87 (dd, *J* = 7.6, 1.4 Hz, 1H), 6.83 – 6.77 (m, 2H), 4.95 – 4.89 (m, 3H), 4.45 (ddd, *J* = 12.7, 8.9, 4.6 Hz, 1H), 3.95 – 3.84 (m, 2H), 3.77 (s, 3H), 0.93 (td, *J* = 7.1, 1.0 Hz, 3H). ^13^C NMR (100 MHz, Chloroform-*d*) δ 193.0, 167.1, 160.0, 138.0, 136.0, 134.4, 130.1, 129.1, 128.9, 120.5, 114.5, 113.8, 78.2, 62.2, 57.1, 55.4, 43.3, 13.8. Minor Diastreoisomer: ^1^H NMR (400 MHz, Chloroform-*d*) δ 7.87 (dt, *J* = 8.3, 1.2 Hz, 2H), 7.58 – 7.52 (m, 1H), 7.42 (t, *J* = 7.7 Hz, 2H), 7.13 (t, *J* = 7.9 Hz, 1H), 6.78 (d, *J* = 7.5 Hz, 1H), 6.74 – 6.68 (m, 2H), 4.83 – 4.72 (m, 2H), 4.39 (dd, *J* = 8.8, 5.2 Hz, 1H), 4.18 (qd, *J* = 7.2, 1.0 Hz, 2H), 3.69 (d, *J* = 1.0 Hz, 2H), 1.17 (td, *J* = 7.2, 1.0 Hz, 2H). ^13^C NMR (100 MHz, Chloroform-*d*) δ 192.8, 167.9, 160.0, 138.5, 136.2, 134.0, 130.2, 129.1, 128.8, 120.2, 114.2, 113.7, 78.2, 62.4, 56.4, 55.3, 43.28, 14.1. HRMS (ESI-TOF) m/z: [M+Na]^+^ Calcd for C_20_H_21_NO_6_ 394.1267; found 394.1276. HPLC analysis: *ee* 70% (major diastereoisomer); 25 cm Chiralpak AD-3 column, *n*-hexane/*i*-PrOH = 70/30 (v/v), flow rate: 1 mL/min, 254 nm; *t*_R_(major) = 7.08 min, *t*_R_(minor) = 9.97 min; *ee* 52% (minor diastereoisomer); *t*_R_(major) = 8.35 min, *t*_R_(minor) = 12.14 min.

##### (3*R*)-ethyl 2-benzoyl-3-(2-fluorophenyl)-4-nitrobutanoate (2h)

The product was prepared by following the general procedure and was obtained as a colorless sticky solid in 74% yield. Major Diastreoisomer: ^1^H NMR (400 MHz, Chloroform-*d*) δ 8.10 – 8.01 (m, 2H), 7.65 – 7.57 (m, 1H), 7.48 (t, *J* = 7.8 Hz, 2H), 7.32 – 7.24 (m, 2H), 7.11 – 7.04 (m, 2H), 5.10 – 4.97 (m, 3H), 4.74 – 4.67 (m, 1H), 3.85 (qd, *J* = 7.1, 3.0 Hz, 2H), 0.89 (t, *J* = 7.1 Hz, 3H). ^13^C NMR (100 MHz, Chloroform-*d*) δ 192.4, 166.9, 134.5, 134.1, 130.4, 130.4, 130.2, 130.1, 129.0, 129.0, 128.9, 128.7, 124.71, 124.68, 116.3, 116.2, 116.0, 116.0, 76.69, 76.67, 62.1, 55.5, 39.2, 13.7. Minor Diastreoisomer: ^1^H NMR (400 MHz, Chloroform-*d*) δ 7.90 – 7.82 (m, 1H), 7.57 – 7.52 (m, 1H), 7.41 (t, *J* = 7.8 Hz, 1H), 7.24 – 7.19 (m, 1H), 7.15 (tdd, *J* = 7.3, 5.3, 1.7 Hz, 1H), 6.99 (td, *J* = 7.5, 1.2 Hz, 1H), 6.91 (ddd, *J* = 11.3, 8.2, 1.2 Hz, 1H), 4.86 – 4.75 (m, 2H), 4.63 (td, *J* = 9.3, 4.8 Hz, 1H), 4.17 (q, *J* = 7.0 Hz, 1H), 1.16 (t, *J* = 7.1 Hz, 2H). ^13^C NMR (100 MHz, Chloroform-*d*) δ 192.9, 167.7, 162.7, 162.2, 135.9, 135.8, 131.4, 131.3, 131.2, 131.2, 129.1, 129.0, 128.9, 128.7, 124.76, 124.72, 123.6, 123.5, 116.3, 116.2, 116.0, 115.9, 76.5, 76.5, 62.4, 54.6, 39.23, 14.0. HRMS (ESI-TOF) m/z: [M+Na]^+^ Calcd for C_19_H_18_FNO_5_ 382.1067; found 382.1049. HPLC analysis: *ee* 99% (major diastereoisomer); 25 cm Chiralpak AD-3 column, *n*-hexane/*i*-PrOH = 70/30 (v/v), flow rate: 1 mL/min, 254 nm; *t*_R_(major) = 7.61 min, *t*_R_(minor) = 9.47 min; *ee* 72% (minor diastereoisomer) *t*_R_(major) = 8.67 min, *t*_R_(minor) = 6.11 min.

##### (3*R*)-ethyl 2-benzoyl-3-(3,5-dimethoxyphenyl)-4-nitrobutanoate (2i)

The product was prepared by following the general procedure and was obtained as a yellow semisolid in 70% yield. Major Diastreoisomer: ^1^H NMR (400 MHz, Chloroform-*d*) δ 8.06 – 7.97 (m, 2H), 7.61 (td, *J* = 7.3, 1.3 Hz, 1H), 7.51 – 7.45 (m, 2H), 6.42 (d, *J* = 2.3 Hz, 2H), 6.35 (s, 1H), 4.91 (dt, *J* = 8.4, 3.3 Hz, 3H), 4.41 (td, *J* = 8.8, 8.4, 4.5 Hz, 1H), 3.94 (q, *J* = 7.1 Hz, 2H), 3.74 (s, 6H), 0.99 – 0.92 (m, 3H). ^13^C NMR (100 MHz, Chloroform-*d*) δ 192.9, 167.9, 161.2, 139.3, 136.3, 134.4, 129.1, 128.9, 106.6, 100.2, 78.0, 62.2, 57.0, 56.3, 55.5, 43.5, 13.8. Minor Diastreoisomer: ^1^H NMR (400 MHz, Chloroform-*d*) δ 7.90 – 7.85 (m, 2H), 7.55 (dd, *J* = 7.3, 1.4 Hz, 1H), 7.44 – 7.38 (m, 2H), 6.32 (d, *J* = 2.2 Hz, 2H), 6.25 (d, *J* = 2.2 Hz, 1H), 4.81 – 4.71 (m, 2H), 4.34 (dd, *J* = 8.7, 5.1 Hz, 1H), 4.21 – 4.13 (m, 2H), 3.66 (d, *J* = 1.1 Hz, 5H), 1.17 (td, *J* = 7.1, 1.1 Hz, 3H). HRMS (ESI-TOF) m/z: [M+Na]^+^ Calcd for C_21_H_23_NO_7_ 424.1372; found 424.1349. HPLC analysis: *ee* 60% (major diastereoisomer); 25 cm Chiralpak AD-3 column, *n*-hexane/*i*-PrOH = 80/20 (v/v), flow rate: 1 mL/min, 254 nm; *t*_R_(major) = 10.74 min, *t*_R_(minor) = 15.55 min; *ee* 61% (minor diastereoisomer); *t*_*R*_ (major) = 11.94 min, *t*_*R*_ (minor) = 18.37 min.

##### (3*S*)-ethyl 2-benzoyl-4-nitro-3-(thiophen-2-yl)butanoate (2j)

The product was prepared by following the general procedure and was obtained as a brown semisolid in 76% yield. Major Diastreoisomer: ^1^H NMR (400 MHz, Chloroform-*d*) δ 8.04 (d, *J* = 8.2 Hz, 2H), 7.46 (dd, *J* = 15.7, 7.9 Hz, 3H), 7.22 (d, *J* = 5.1 Hz, 1H), 6.97 (d, *J* = 3.3 Hz, 1H), 6.94 – 6.91 (m, 1H), 5.01 – 4.94 (m, 3H), 4.73 (td, *J* = 8.3, 4.7 Hz, 1H), 3.98 (q, *J* = 7.1 Hz, 2H), 1.01 (t, *J* = 7.1 Hz, 3H). ^13^C NMR (100 MHz, Chloroform-*d*) δ 192.7, 167.0, 138.9, 135.9, 134.5, 129.1, 128.9, 127.2, 127.1, 125.6, 78.5, 62.4, 57.8, 38.7, 13.9. Minor Diastreoisomer: ^1^H NMR (400 MHz, Chloroform-*d*) δ 7.92 (d, *J* = 7.8 Hz, 2H), 7.60 (dt, *J* = 14.7, 7.3 Hz, 2H), 7.13 (d, *J* = 5.1 Hz, 1H), 6.83 (t, *J* = 4.4 Hz, 1H), 4.82 (ddd, *J* = 17.9, 7.2, 4.2 Hz, 3H), 4.17 (q, *J* = 7.2 Hz, 2H), 1.17 (t, *J* = 7.1 Hz, 2H). ^13^C NMR (100 MHz, Chloroform-*d*) δ 192.9, 167.5, 139.3, 136.0, 134.2, 129.1, 128.9, 127.2, 127.1, 125.5, 78.6, 62.5, 57.1, 38.8, 14.1. HRMS (ESI-TOF) m/z: [M+Na]^+^ Calcd for C_17_H_17_NO_5_S 370.0725; found 370.0706. HPLC analysis: *ee* 84% (major diastereoisomer); 25 cm Chiralpak AD-3 column, *n*-hexane/*i*-PrOH = 60/40 (v/v), flow rate: 1 mL/min, 254 nm; *t*_R_(major) = 11.87 min, *t*_R_(minor) = 7.17 min; *ee* 83% (minor diastereoisomer); *t*_*R*_ (major) = 13.07 min, *t*_*R*_ (minor) = 8.54 min.

##### (3*S*)-ethyl 2-benzoyl-3-cyclohexyl-4-nitrobutanoate (2k)

The product was prepared by following the general procedure and was obtained as a colorless liquid in 71% yield. ^1^H NMR (400 MHz, Chloroform-*d*) δ 7.98 (d, *J* = 7.5 Hz, 2H), 7.62 (q, *J* = 7.5 Hz, 1H), 7.50 (q, *J* = 7.9 Hz, 2H), 4.86 – 4.66 (m, 3H), 4.19 – 4.10 (m, 2H), 2.96 (ddt, *J* = 21.4, 10.0, 6.1 Hz, 1H), 1.79 (dt, *J* = 28.7, 15.3 Hz, 3H), 1.66 (d, *J* = 13.5 Hz, 2H), 1.53 (ddd, *J* = 14.7, 11.5, 3.2 Hz, 1H), 1.18 (td, *J* = 7.1, 4.7 Hz, 6H), 1.08 – 0.97 (m, 2H). ^13^C NMR (100 MHz, Chloroform-*d*) δ 195.0, 168.8, 135.8, 134.2, 129.2, 128.8, 75.4, 62.0, 53.1, 42.3, 40.3, 30.7, 30.2, 26.5, 26.4, 26.2, 14.1. HRMS (ESI-TOF) m/z: [M+Na]^+^ Calcd for C_19_H_25_NO_5_ 370.1630; found 370.1661. HPLC analysis: *ee* 56% (major diastereoisomer); 25 cm Chiralpak AD-3 column, *n*-hexane/*i*-PrOH = 70/30 (v/v), flow rate: 1 mL/min, 254 nm; *t*_R_(major) = 13.81 min, *t*_R_(minor) = 11.40 min; *ee* 54% (minor diastereoisomer); *t*_R_(major) = 12.99 min, *t*_R_(minor) = 9.299 min.

##### (3*R*)-ethyl 2-benzoyl-5-methyl-3-(nitromethyl)hexanoate (2l)

The product was prepared by following the general procedure and was obtained as a colorless liquid in 68% yield. ^1^H NMR (400 MHz, Chloroform-*d*) δ 7.99 (t, *J* = 7.3 Hz, 2H), 7.62 (q, *J* = 6.6 Hz, 1H), 7.50 (q, *J* = 7.5 Hz, 2H), 4.81 – 4.69 (m, 1H), 4.68 – 4.56 (m, 2H), 4.20 – 4.09 (m, 2H), 3.08 (dp, *J* = 14.6, 6.4 Hz, 1H), 1.71 (tt, *J* = 14.4, 7.2 Hz, 1H), 1.38 (ddd, *J* = 24.5, 12.2, 5.8 Hz, 1H), 1.29 – 1.23 (m, 1H), 1.20 – 1.13 (m, 3H), 0.95 – 0.90 (m, 3H), 0.88 (d, *J* = 6.4 Hz, 3H). ^13^C NMR (100 MHz, Chloroform-*d*) δ 194.5, 168.5, 136.4, 134.2, 129.1, 128.8, 76.8, 62.0, 54.7, 39.4, 35.3, 25.4, 22.9, 22.1, 14.1. HRMS (ESI-TOF) m/z: [M+Na]^+^ Calcd for C_17_H_23_NO_5_ 344.1474; found 344.1452. HPLC analysis: *ee* 50% (major diastereoisomer); 25 cm Chiralpak AD-3 column, *n*-hexane/*i*-PrOH = 95/05 (v/v), flow rate: 1 mL/min, 254 nm; *t*_R_(major) = 7.92 min, *t*_R_(minor) = 12.53 min; *ee* 52% (minor diastereoisomer *t*_*R*_ (major) = 7.40 min, *t*_*R*_ (minor) = 10.11 min.

##### (3*R*)-ethyl 2-benzoyl-4-nitro-3-(1-((4-nitrophenyl)sulfonyl)-1H-indol-3-yl)butanoate (2m)

The product was prepared by following the general procedure and was obtained as a yellow solid in 74% yield. Major Diastreoisomer: ^1^H NMR (400 MHz, Chloroform-*d*) δ 8.20 (d, *J* = 8.9 Hz, 2H), 7.96 – 7.90 (m, 3H), 7.75 (d, *J* = 8.9 Hz, 1H), 7.65 – 7.55 (m, 4H), 7.52 – 7.42 (m, 3H), 5.12 (dd, *J* = 9.4, 3.4 Hz, 1H), 4.90 (dd, *J* = 12.8, 7.3 Hz, 1H), 4.82 (dd, *J* = 12.7, 4.2 Hz, 1H), 4.74 – 4.64 (m, 1H), 3.85 (qt, *J* = 6.9, 3.6 Hz, 2H), 0.82 (t, *J* = 7.1 Hz, 3H). ^13^C NMR (100 MHz, Chloroform-*d*) δ 192.9, 167.1, 150.8, 142.7, 135.7, 134.7, 129.2, 128.8, 128.2, 126.3, 125.1, 124.7, 119.6, 114.0, 77.1, 62.4, 55.8, 34.2, 13.7. Minor Diastreoisomer: ^1^H NMR (400 MHz, Chloroform-*d*) δ 8.10 – 8.06 (m, 1H), 8.05 – 7.99 (m, 2H), 7.88 (dd, *J* = 7.7, 1.5 Hz, 1H), 7.78 – 7.72 (m, 1H), 7.34 (dddd, *J* = 13.8, 7.5, 6.0, 1.6 Hz, 3H), 5.13 (d, *J* = 3.5 Hz, 1H), 5.02 (dd, *J* = 12.9, 7.7 Hz, 1H), 4.97 – 4.93 (m, 1H), 4.16 (q, *J* = 7.1 Hz, 1H), 1.13 (t, *J* = 7.1 Hz, 1H). ^13^C NMR (100 MHz, Chloroform-*d*) δ 192.3, 167.4, 150.6, 142.7, 135.0, 134.5, 129.8, 128.8, 128.0, 126.3, 125.1, 124.6, 119.9, 113.9, 77.1, 62.6, 54.9, 34.3, 14.0. HRMS (ESI-TOF) m/z: [M+Na]^+^ Calcd for C_27_H_30_N_3_O_9_S 588.1053; found 588.1020. HPLC analysis: *ee* 87% (major diastereoisomer); 25 cm Chiralpak AD-3 column, *n*-hexane/*i*-PrOH = 50/50 (v/v), flow rate: 1 mL/min, 254 nm; *t*_R_(major) = 17.70 min, *t*_R_(minor) = 11.00 min; *ee* 54% (minor diastereoisomer); *t*_R_(major) = 18.51 min, *t*_R_(minor) = 11.45 min.

##### (3*R*)-ethyl 2-benzoyl-4-nitro-3-(quinolin-4-yl)butanoate (2n)

The product was prepared by following the general procedure and was obtained as a blackish semisolid in 70% yield. Major Diastreoisomer: ^1^H NMR (400 MHz, Chloroform-*d*) δ 8.82 (dd, *J* = 4.5, 1.5 Hz, 1H), 8.29 (d, *J* = 8.4 Hz, 1H), 8.17 – 8.12 (m, 1H), 8.03 – 7.97 (m, 2H), 7.80 – 7.71 (m, 2H), 7.49 – 7.39 (m, 3H), 7.36 (dd, *J* = 4.6, 1.5 Hz, 1H), 5.44 (td, *J* = 8.6, 4.3 Hz, 1H), 5.22 – 5.08 (m, 3H), 3.76 (qd, *J* = 7.1, 1.5 Hz, 2H), 0.73 (td, *J* = 7.1, 1.6 Hz, 3H). ^13^C NMR (100 MHz, Chloroform-*d*) δ 192.7, 166.8, 149.7, 148.7, 142.9, 135.7, 134.7, 130.6, 130.1, 129.1, 128.8, 127.8, 126.6, 122.7, 119.5, 62.5, 56.1, 36.6, 13.5. Minor Diastreoisomer: ^1^H NMR (400 MHz, Chloroform-*d*) δ 8.71 (dd, *J* = 4.6, 1.5 Hz, 1H), 8.22 (d, *J* = 8.5 Hz, 1H), 8.11 (d, *J* = 8.3 Hz, 1H), 7.88 – 7.82 (m, 1H), 7.71 – 7.65 (m, 2H), 7.62 – 7.53 (m, 2H), 7.20 (dd, *J* = 4.6, 1.6 Hz, 1H), 5.35 (td, *J* = 8.0, 4.9 Hz, 1H), 5.04 – 4.90 (m, 2H), 4.17 (qd, *J* = 7.1, 1.6 Hz, 1H), 1.14 (td, *J* = 7.1, 1.6 Hz, 2H). ^13^C NMR (100 MHz, Chloroform-*d*) δ 192.3, 167.5, 149.5, 148.7, 143.6, 135.6, 134.4, 130.6, 130.1, 129.0, 128.8, 127.9, 126.4, 122.6, 119.5, 62.7, 55.6, 36.6, 14.0. HRMS (ESI-TOF) m/z: [M+Na]^+^ Calcd for C_22_H_21_N_2_O_5_ 393.1450; found 393.1427. HPLC analysis: *ee* 70% (major diastereoisomer); 25 cm Chiralpak AD-3 column, *n*-hexane/*i*-PrOH = 75/25 (v/v), flow rate: 1 mL/min, 254 nm; *t*_R_(major) = 16.17 min, *t*_R_(minor) = 10.86 min; *ee* 35% (minor diastereoisomer), *t*_*R*_ (major) = 13.76 min, *t*_*R*_ (minor) = 10.20 min.

##### (3*S*)-ethyl 3-(benzo[d][1,3]dioxol-5-yl)-2-(4-methoxybenzoyl)-4-nitrobutanoate (2o; Barnes et al., 2002)

The product was prepared with 1.0 mmol scale of **3o** following the general procedure with catalyst **F** and product **2o** was obtained as a reddish gummy liquid in 55% yield (0.228 g). Major Diastreoisomer: ^1^H NMR (400 MHz, Chloroform-*d*) δ 7.89 (d, *J* = 8.9 Hz, 2H), 6.91 (d, *J* = 9.0 Hz, 2H), 6.70 (d, *J* = 1.7 Hz, 1H), 6.68 – 6.61 (m, 2H), 5.90 – 5.83 (m, 2H), 4.95 – 4.85 (m, 2H), 4.83 – 4.78 (m, 1H), 4.34 (td, *J* = 8.8, 5.0 Hz, 1H), 4.18 (q, *J* = 7.0 Hz, 2H), 3.86 (s, 3H), 1.19 (t, *J* = 7.1 Hz, 3H). ^13^C NMR (100 MHz, Chloroform-*d*) δ 191.0, 167.3, 164.7, 148.1, 147.7, 131.6, 130.1, 129.0, 122.0, 114.3, 108.7, 101.4, 78.5, 62.1, 57.0, 55.8, 43.1, 29.9, 13.9. Minor Diastreoisomer: ^1^H NMR (400 MHz, Chloroform-*d*) δ 8.05 (d, *J* = 8.9 Hz, 1H), 6.96 (d, *J* = 8.9 Hz, 1H), 6.78 – 6.73 (m, 1H), 5.94 (s, 1H), 4.75 (dd, *J* = 12.6, 4.4 Hz, 1H), 4.66 (dd, *J* = 12.6, 8.7 Hz, 1H), 3.96 – 3.91 (m, 1H), 3.89 (s, 1H), 0.98 (t, *J* = 7.1 Hz, 1H). ^13^C NMR (100 MHz, Chloroform-*d*) δ 191.0, 167.3, 164.7, 148.1, 147.7, 131.3, 130.1, 129.0, 122.0, 114.2, 108.7, 101.4, 78.4, 62.3, 56.9, 56.3, 43.1, 29.9, 14.1. HRMS (ESI-TOF) m/z: [M+Na]^+^ Calcd for C_21_H_21_NO_8_ 438.1165; found 438.1162. HPLC analysis: *ee* 74% (major diastereoisomer); 25 cm Chiralpak AD-3 column, *n*-hexane/*i*-PrOH =40/60 (v/v), flow rate: 1 mL/min, 254 nm; *t*_R_(major) = 16.71 min, *t*_R_(minor) = 9.90 min; *ee* 76% (minor diastereoisomer); *t*_*R*_ (major) = 12.59 min, *t*_*R*_ (minor) = 9.26 min.

##### (2*R*,3*R*,4*S*)-ethyl 4-(benzo[d][1,3]dioxol-5-yl)-2-(4-methoxyphenyl)pyrrolidine-3-carboxylate (7)

The nitro ester **2o** (0.20 g, 0.482 mmol) in 5 mL of ethanol was hydrogenated at high pressure with 0.50 g of Raney nickel (washed with ethanol three times before use). After full conversion of the starting material the catalyst was filtered and the solution was concentrated and the residue was directly taken for the next step. This compound must be kept away from air as it is easily hydroxylated at the 3-position and this byproduct could not be separated by chromatography. The imine (0.166 g, 0.452 mmol) was dissolved in 3 mL of THF and 6 mL of ethanol. Sodium cyanoborohydride (0.032 g, 0.50 mmol) and pinch of bromocresol green were added. To this blue solution was added dropwise a solution of concentrated HCl in ethanol (1:2), at such a rate that the color was kept at light yellow-green. After the yellow color persisted without additional HCl, the solution was stirred an additional 20 min. The solution was concentrated in vacuum and then partitioned between CHCl_3_ and KHCO_3_ solution. The organic phase was separated, dried (Na_2_SO_4_) and concentrated. The residue was chromatographed on silica gel eluting with 80% EtOAc-20% hexanes to give 0.152 g of yellow gummy liquid, yield 84% over two steps. ^1^H NMR (400 MHz, Chloroform-*d*) δ 7.29 (d, *J* = 6.7 Hz, 2H), 6.86 (d, *J* = 7.1 Hz, 2H), 6.79 (s, 1H), 6.74 (s, 2H), 5.92 (s, 2H), 4.62 (d, *J* = 5.1 Hz, 1H), 3.88 – 3.81 (m, 1H), 3.79 (d, *J* = 1.3 Hz, 3H), 3.69 (q, *J* = 7.4, 5.9 Hz, 3H), 3.59 – 3.48 (m, 2H), 3.39 (s, 1H), 0.81 (td, *J* = 7.1, 1.3 Hz, 3H). ^13^C NMR (100 MHz, Chloroform-*d*) δ 171.4, 158.8, 147.7, 146.4, 132.7, 131.0, 127.6, 121.1, 113.8, 108.5, 108.2, 101.0, 66.1, 59.9, 56.4, 55.4, 50.5, 49.6, 14.0. HRMS (ESI-TOF) m/z: [M+Na]^+^ Calcd for C_21_H_24_NO_5_ 370.1654; found 374.1647.

##### *N,N*-Dibutylbromoacetamide

Using the method of Weaver and Whaley (1947) bromoacetyl bromide (1.0 g, 5 mmol) was dissolved in 2 mL of dichloroethane and added over a 2 min period to 1.3 g (10 mmol) of dibutylamine dissolved in 5 mL of dichloroethane cooled to −45°C. The mixture was stirred at −30°C for 20 min and at room temp for 30 min. Additional (10 mL) dichloroethane was added and the solution was washed with 4 mL of water twice. The solvents were removed in vacuum, and the heptane (15 mL) was added to the residue. Some insoluble material was removed by filtration, and the solution was concentrated in vacuum to yield 1.15 g (92%) of the desired product.

##### (2*R*,3*R*,4*S*)-ethyl 4-(benzo[d][1,3]dioxol-5-yl)-1-(2-(dibutylamino)-2-oxoethyl)-2-(4-methoxyphenyl)pyrrolidine-3-carboxylate (8)

The pyrrolidine **7** (55 mg, 0.15 mmol) was combined with 38 mg (0.15 mmol) of the *N,N*-dibutylbromoacetamide in 3 mL of acetonitrile; 0.5 mL (2.9 mmol) of DIPEA was added, and the solution was allowed to stir overnight at ambient temperature. Solvents were removed in vacuum; the residue was partitioned between EtOAc and aqueous 1 N phosphoric acid. The organic layer was washed with sodium bicarbonate solution and brine, then dried over sodium sulfate, filtered and concentrated. The residue was purified by flash chromatography on silica gel, eluting with 2:1 hexanes/EtOAc afforded compound **8** as a gummy liquid, yield 63%. ^1^H NMR (400 MHz, Chloroform-*d*) δ 7.33 (d, *J* = 8.5 Hz, 2H), 6.84 (d, *J* = 8.6 Hz, 2H), 6.79 (s, 1H), 6.71 (d, *J* = 2.3 Hz, 2H), 5.90 (s, 2H), 4.80 (d, *J* = 6.1 Hz, 1H), 4.08 (td, *J* = 9.3, 6.4 Hz, 1H), 3.98 (dd, *J* = 9.9, 8.0 Hz, 1H), 3.79 (s, 3H), 3.70 – 3.58 (m, 3H), 3.53 – 3.45 (m, 2H), 3.36 (d, *J* = 16.2 Hz, 1H), 3.27 (t, *J* = 7.7 Hz, 2H), 3.14 – 3.04 (m, 2H), 1.48 (ddd, *J* = 9.8, 5.9, 2.6 Hz, 2H), 1.34 – 1.26 (m, 4H), 1.18 (q, *J* = 7.4 Hz, 2H), 0.93 (t, *J* = 7.3 Hz, 3H), 0.84 (t, *J* = 7.3 Hz, 3H), 0.77 (t, *J* = 7.1 Hz, 3H). ^13^C NMR (100 MHz, Chloroform-*d*) δ 170.8, 170.3, 159.2, 147.5, 146.0, 133.7, 131.4, 129.0, 120.8, 113.6, 108.4, 108.1, 100.9, 69.2, 59.7, 57.8, 55.4, 55.3, 51.7, 46.9, 45.7, 45.3, 31.1, 30.0, 20.5, 20.1, 14.1, 13.9. HRMS (ESI-TOF) m/z: [M+Na]^+^ Calcd for C_31_H_43_N_2_O_6_ 539.3121; found 539.3128.

##### (2*R*,3*R*,4*S*)-4-(benzo[d][1,3]dioxol-5-yl)-1-(2-(dibutylamino)-2-oxoethyl)-2-(4-methoxyphenyl)pyrrolidine-3-carboxylic acid (9, ABT-627; Winn et al., 1996)

The product **8** (0.051 g, 0.095 mmol) was dissolved in 4 mL of ethanol; 1 mL of 2.5 N aqueous sodium hydroxide (2.5 mmol) was added, and the resultant solution was stirred overnight at ambient temperature. Solvents were removed in vacuum; the residue was taken up in water and extracted with ether. The aqueous phase was acidified to pH 5 with aqueous 1 N phosphoric acid and extracted with EtOAc. The organic extracts were washed with brine, dried over sodium sulfate, and filtered. The solvents were removed in vacuum to give 26 mg of the title compound **9** (ABT-627) as colorless sticky liquid, yield 54%. ^1^H NMR (400 MHz, Chloroform-*d*) δ 7.32 (d, *J* = 8.3 Hz, 2H), 7.04 (d, *J* = 1.7 Hz, 1H), 6.88 – 6.82 (m, 3H), 6.75 – 6.71 (m, 1H), 5.95 – 5.92 (m, 2H), 3.79 (s, 3H), 3.75 (d, *J* = 9.5 Hz, 1H), 3.60 (ddd, *J* = 9.6, 6.9, 3.0 Hz, 1H), 3.53 – 3.46 (m, 1H), 3.43 – 3.34 (m, 3H), 3.02 (tdd, *J* = 16.7, 11.8, 6.1 Hz, 5H), 2.76 (d, *J* = 13.2 Hz, 1H), 1.48 – 1.43 (m, 2H), 1.26 (q, *J* = 7.3 Hz, 4H), 1.07 (q, *J* = 7.5 Hz, 2H), 0.88 (t, *J* = 7.3 Hz, 3H), 0.82 (t, *J* = 7.3 Hz, 3H). ^13^C NMR (100 MHz, Chloroform-*d*) δ 177.5, 169.7, 159.4, 147.9, 146.1, 139.2, 131.0, 129.5, 120.4, 116.1, 114.0, 108.1, 107.5, 100.8, 72.6, 61.4, 60.3, 55.2, 55.2, 46.9, 45.8, 45.3, 31.0, 29.6, 20.2, 19.9, 13.84, 13.79. HRMS (ESI-TOF) m/z: [M+Na]^+^ Calcd for C_29_H_39_N_2_O_6_ 511.2808; found 511.2807. HPLC analysis: *ee* 67% (major diastereoisomer); 25 cm Chiralpak AD-3 column, *n*-hexane/*i*-PrOH = 20/80 (v/v), flow rate: 0.4 mL/min, 254 nm; *t*_R_(major) = 19.59 min, *t*_R_(minor) = 11.21 min.

## Data Availability Statement

All datasets generated for this study are included in the article/Supplementary Material.

## Author Contributions

SHaj conceived the project. SA, BJ, and SHaz carried out the chemical synthesis and analysis of data. All authors were involved in manuscript writing and revision. Also have made substantial, direct and intellectual contributions to the work, and approved it for publication.

### Conflict of Interest

The authors declare that the research was conducted in the absence of any commercial or financial relationships that could be construed as a potential conflict of interest.
