# Supplementary material for: Organocatalytic Enantioselective Conjugate Addition of Nitromethane to Benzylidene-2-Benzoyl Acetate: Asymmetric Synthesis of ABT – 627, an Endothelin Receptor Antagonist
Source: Front Chem. 2020 Mar 5;8:135. doi: 10.3389/fchem.2020.00135 (PMC7066299; doi:10.3389/fchem.2020.00135)
Supplement: Supplementary file 1 [file Data_Sheet_1.pdf]

Supplementary Material for

Organocatalytic enantioselective conjugate addition of nitromethane to benzylidine-2-benzoyl acetate: Asymmetric synthesis of ABT – 627, an endothelin receptor antagonist

Saumen Hajra[* 1], Sk Mohammad Aziz[2,3], Bibekananda Jana[1,2], Sunit Hazra[2,4]

[1]Centre of Biomedical Research, Sanjay Gandhi Post-Graduate Institute of Medical Sciences Campus, Raebareli Road, Lucknow 226014, India
[2]Department of Chemistry, Indian Institute of Technology Kharagpur, Kharagpur 721302, India
[3]Present address: Department of Chemistry, Narajole Raj College, Narajole, West Midnapore 721211, India
[4]Present address: Department of Applied Chemistry, Graduate School of Engineering, Osaka University, Suita, Osaka – 5650871, Japan

Table of Contents

List of contents	Page
Title, authors' name, address and tables	S1
NMR copies of compounds	S2-S19
HPLC copies of compounds	S20-S35

^1H NMR and ^{13}C NMR Spectra of all Compounds:

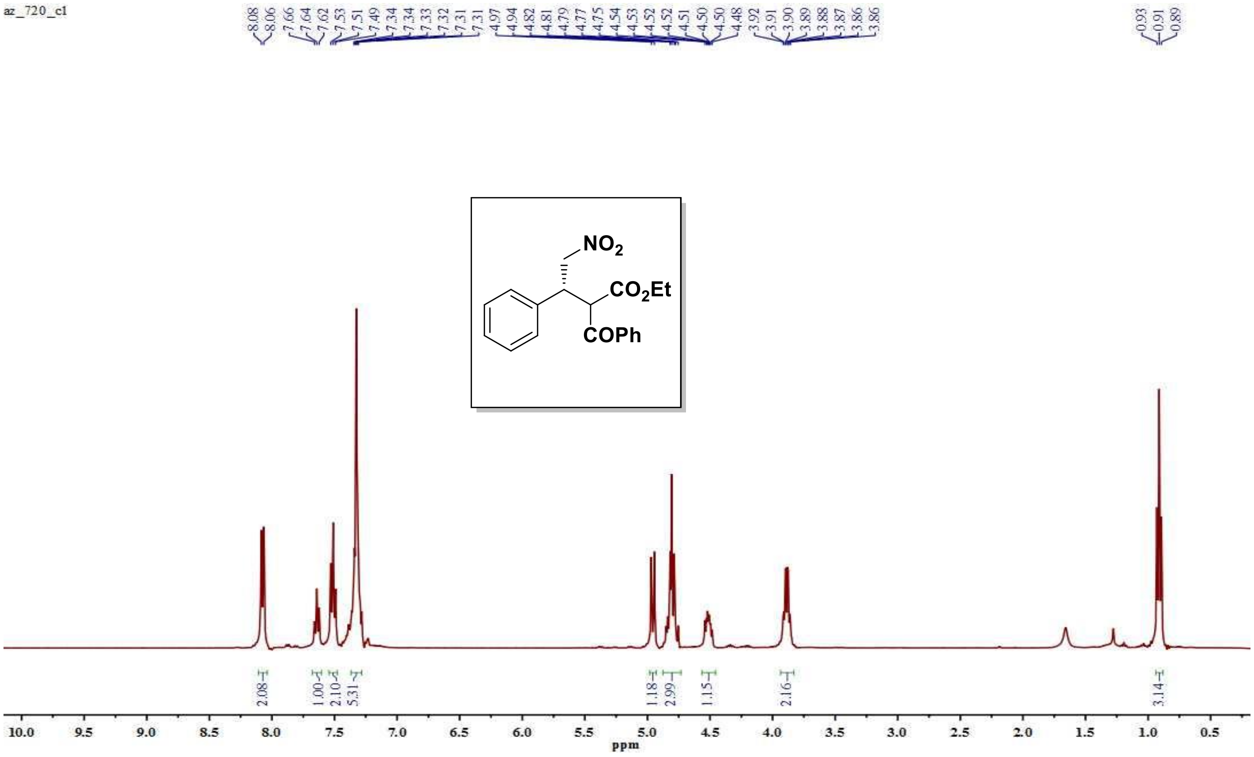

^1H NMR Spectrum of Compound **2a** (400 MHz, $CDCl_3$)

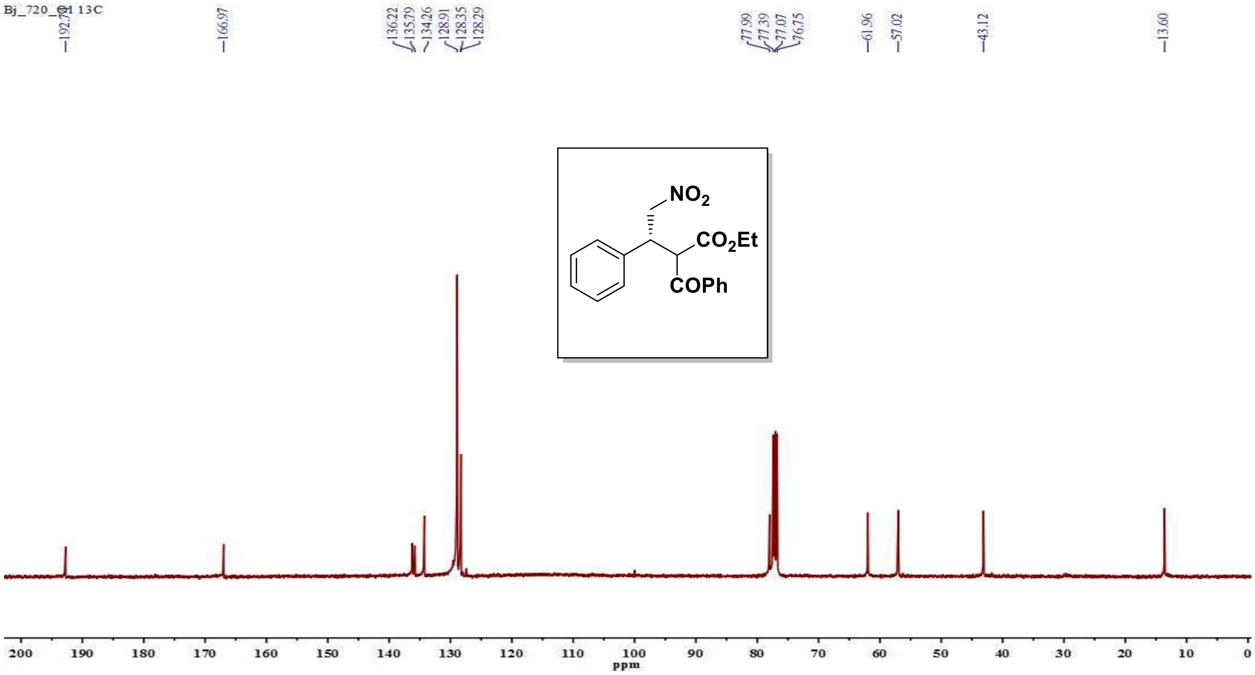

^{13}C NMR Spectrum of Compound **2a** (100 MHz, $CDCl_3$)

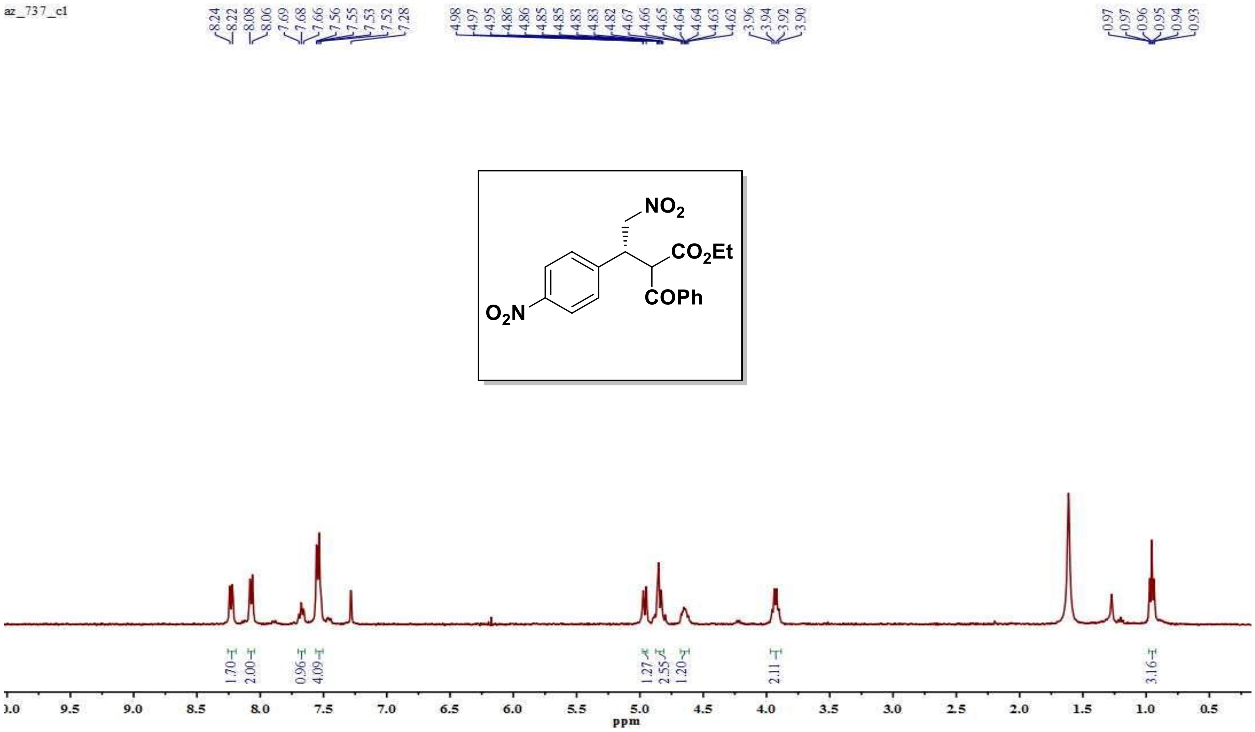

^{1}H NMR Spectrum of Compound **2b** (400 MHz, $CDCl_3$)

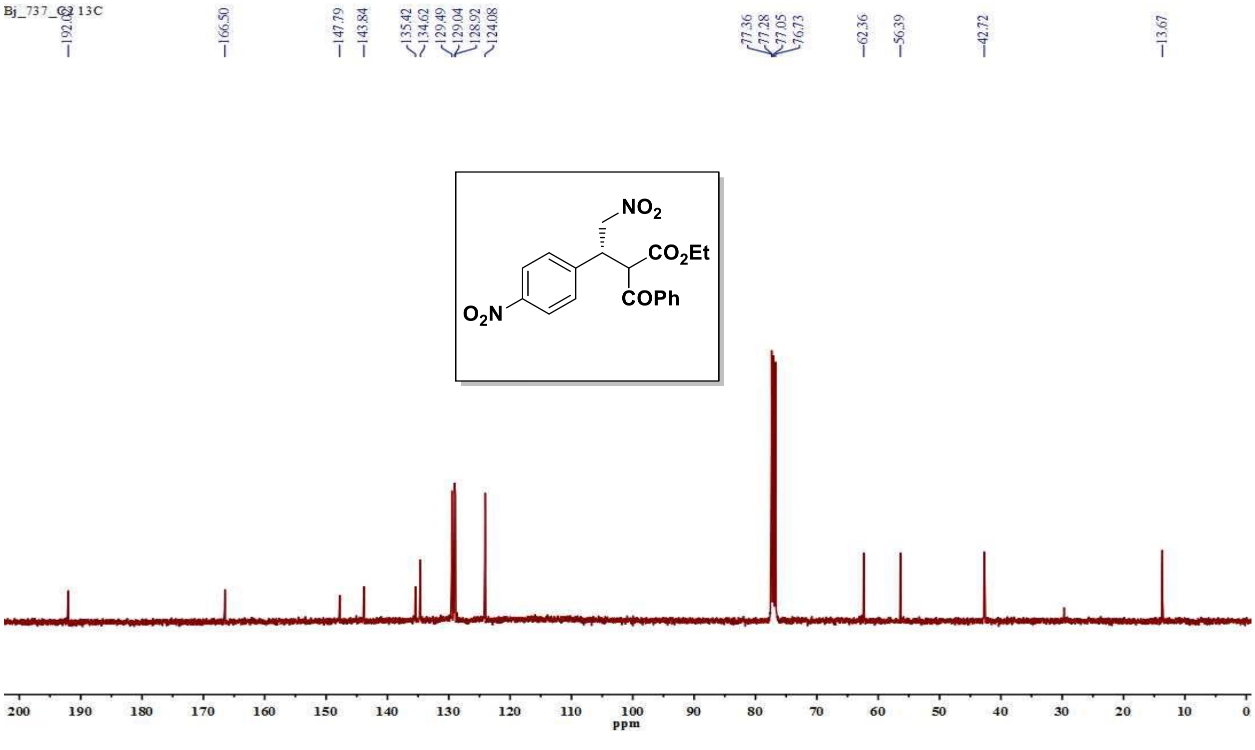

^{13}C NMR Spectrum of Compound **2b** (100 MHz, $CDCl_3$)

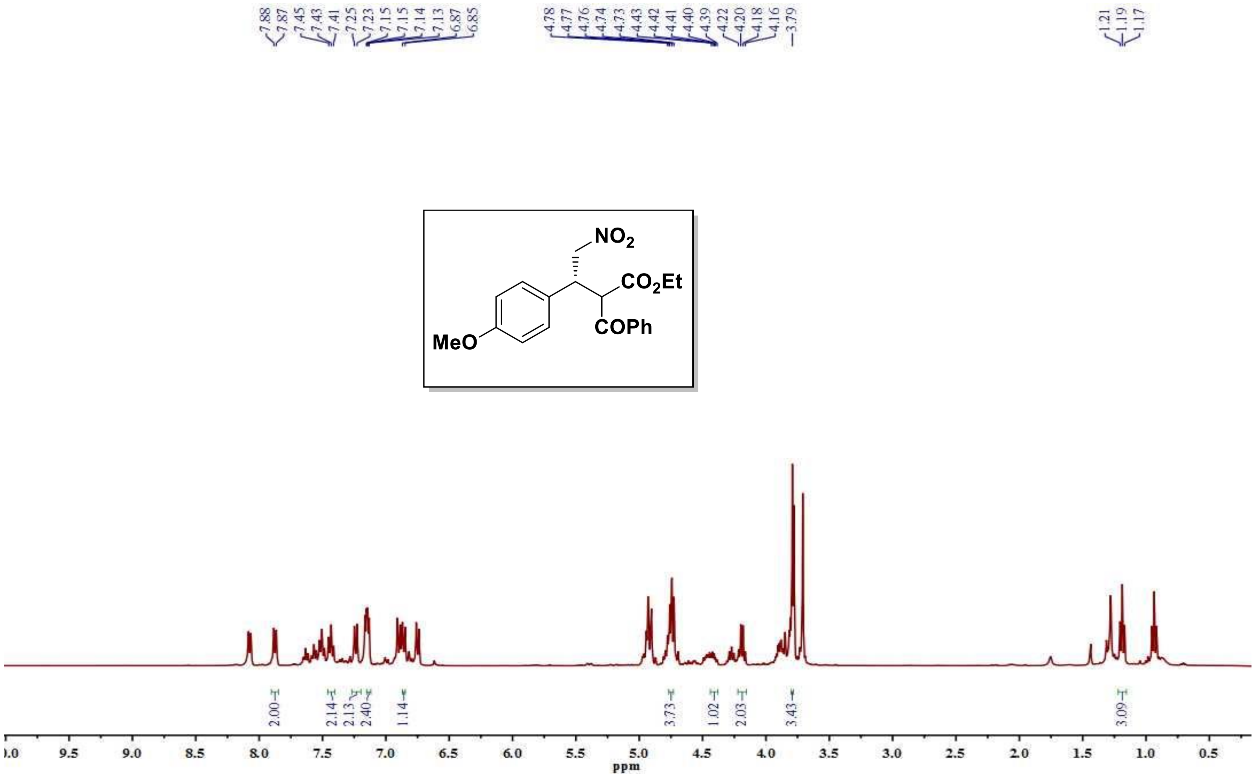

^{1}H NMR Spectrum of Compound **2c** (400 MHz, $CDCl_3$)

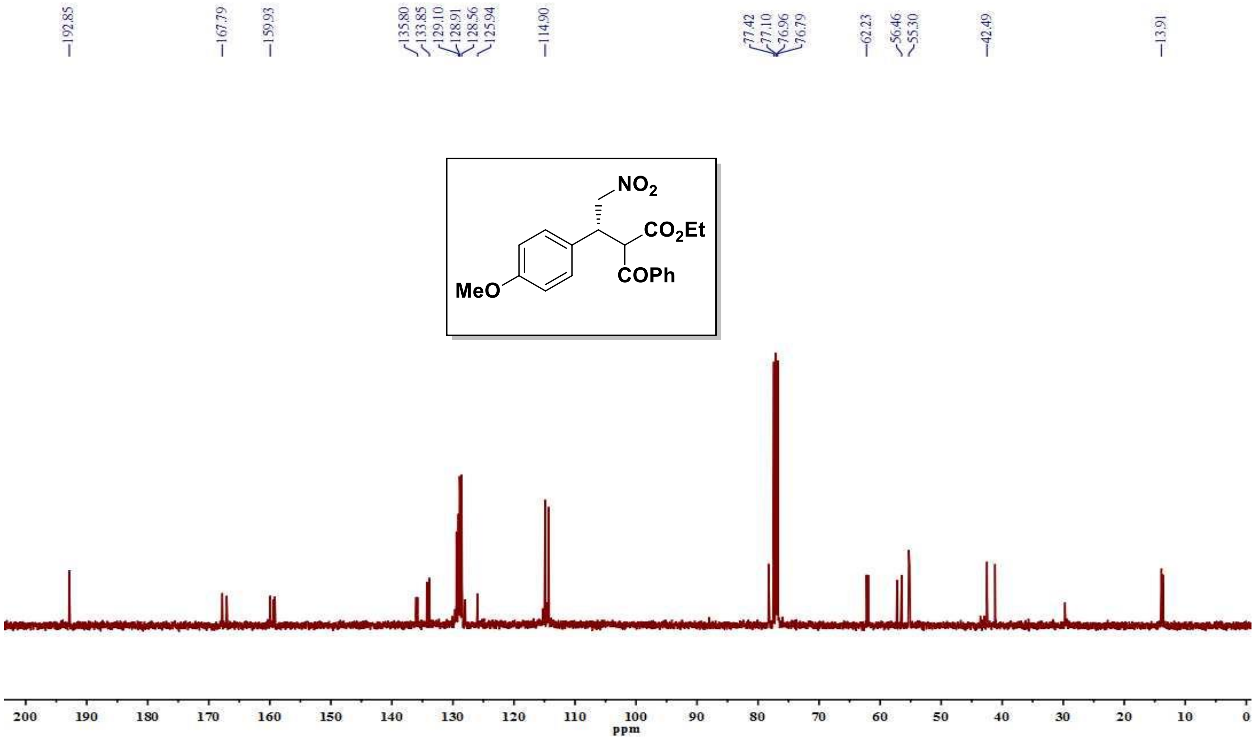

^{13}C NMR Spectrum of Compound **2c** (100 MHz, $CDCl_3$)

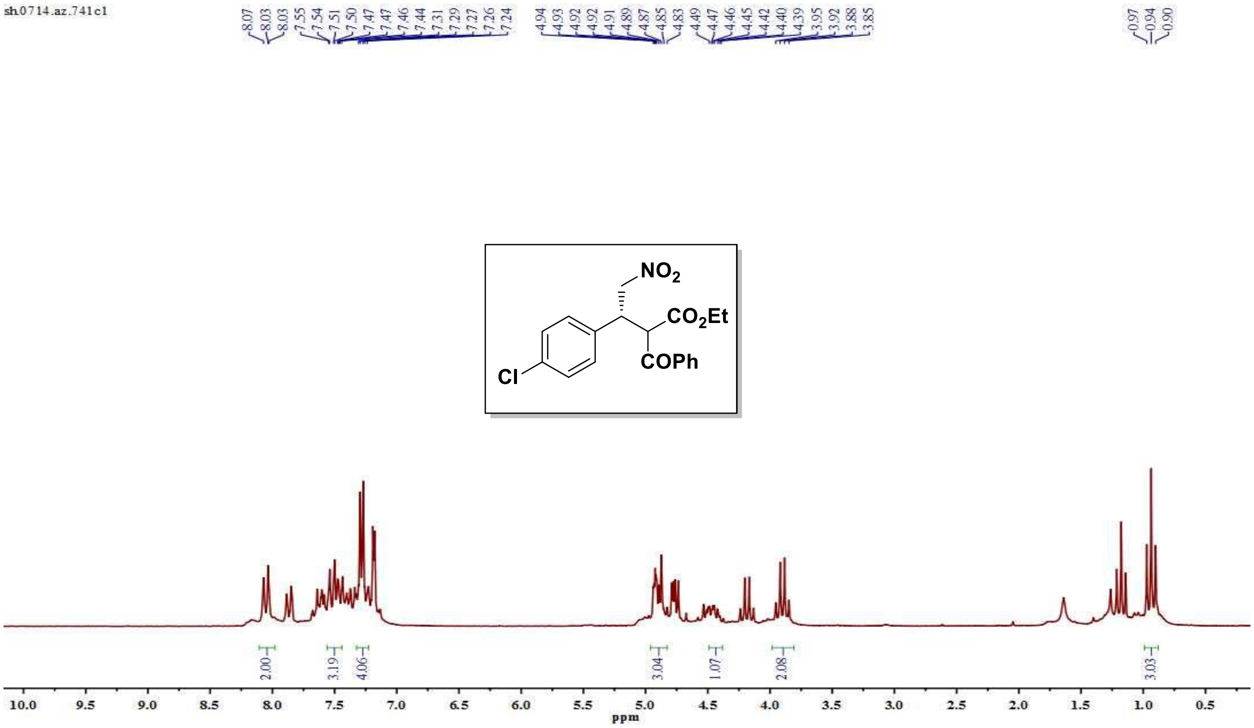

^{1}H NMR Spectrum of Compound **2d** (Major Diastereoisomer) (200 MHz, $CDCl_3$)

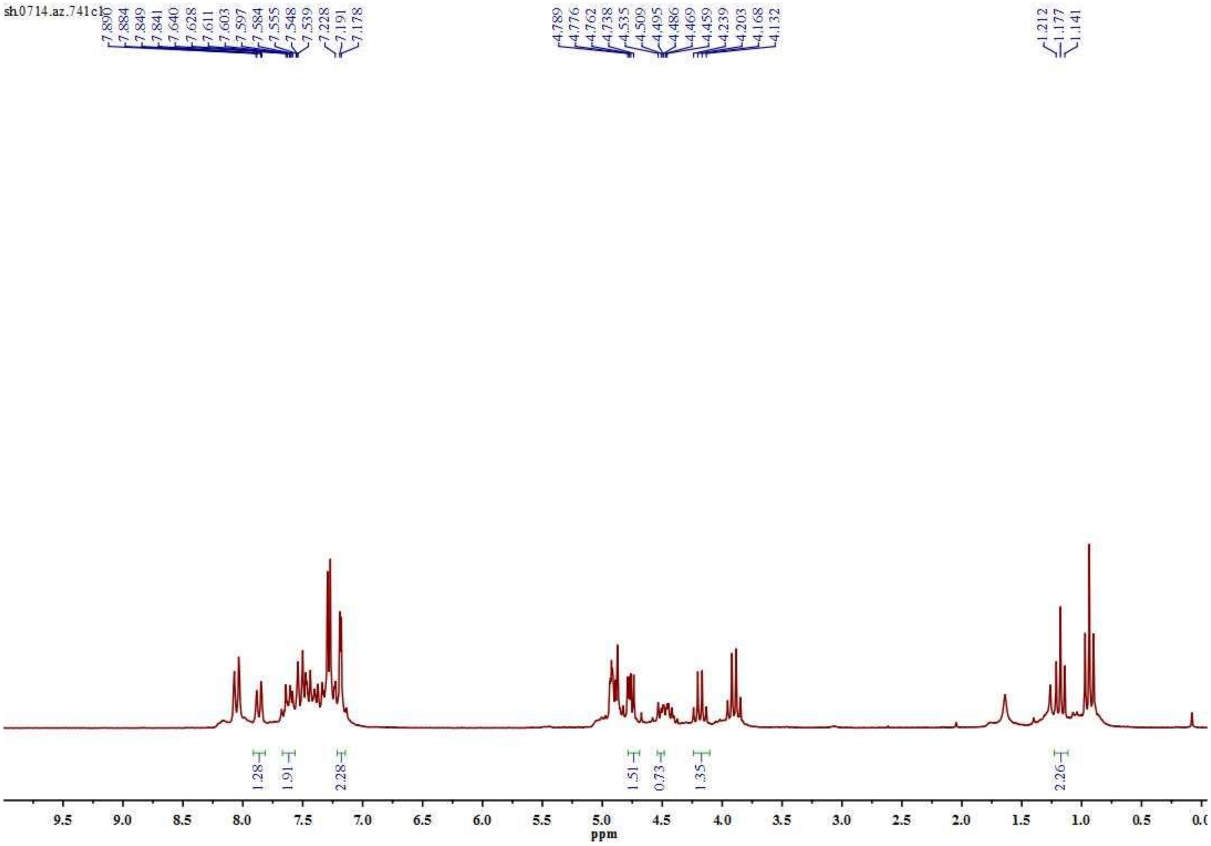

^{1}H NMR Spectrum of Compound **2d** (Minor Diastereoisomer) (200 MHz, $CDCl_3$)

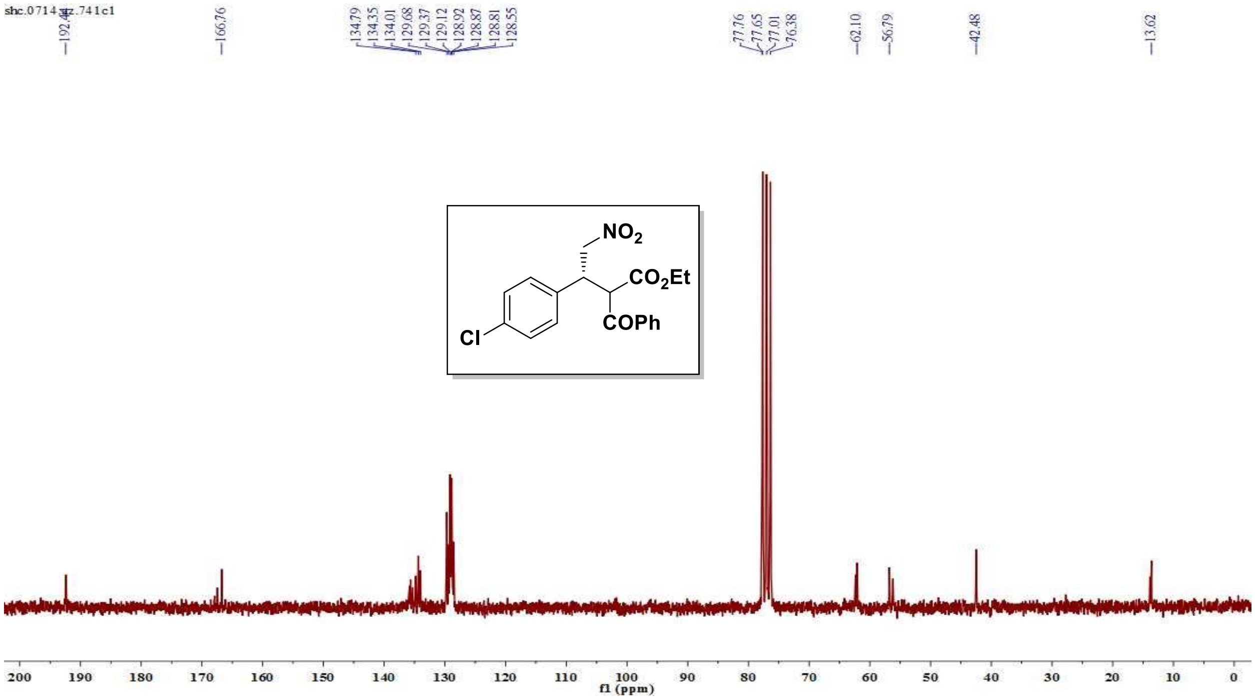

^{13}C NMR Spectrum of Compound **2d** (Major Diastereoisomer) (50 MHz, $CDCl_3$)

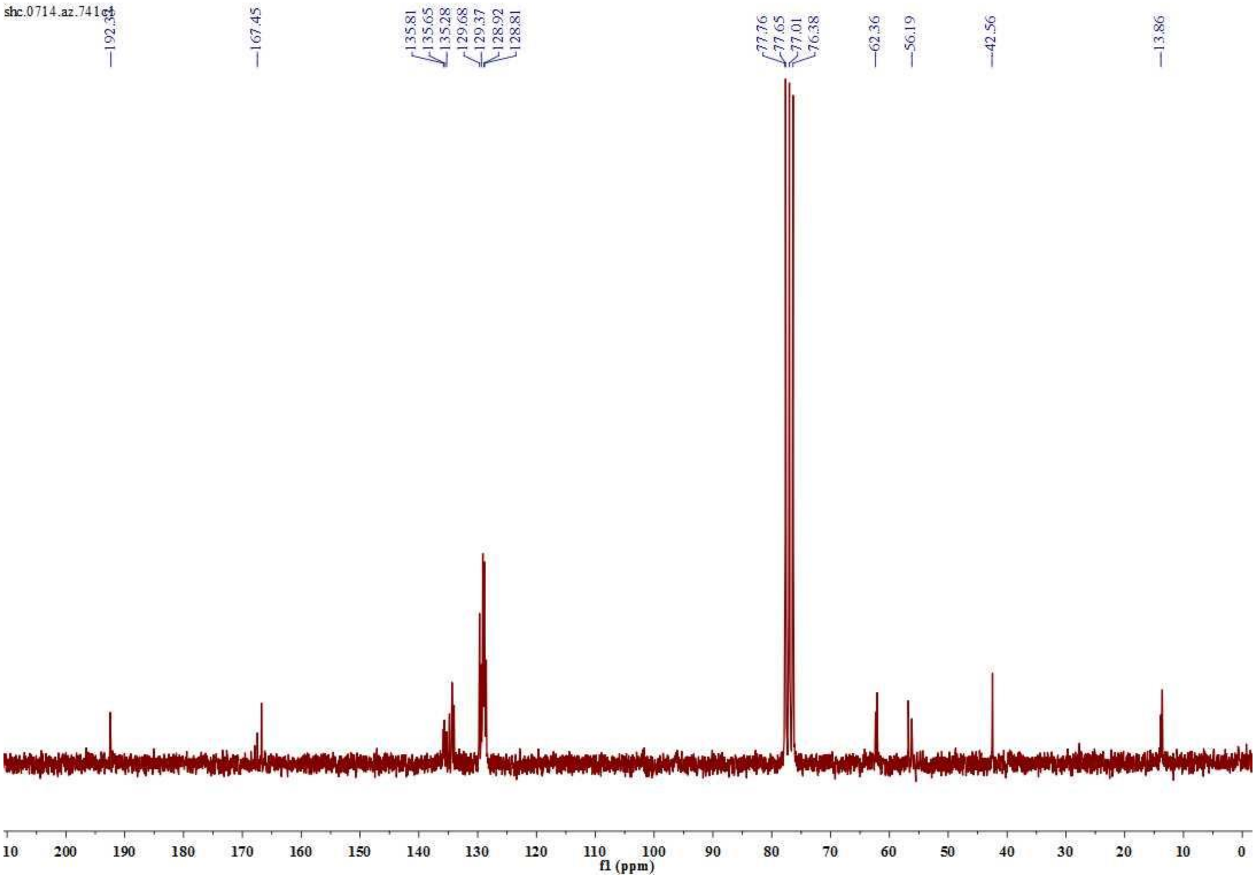

^{13}C NMR Spectrum of Compound **2d** (Minor Diastereoisomer) (50 MHz, $CDCl_3$)

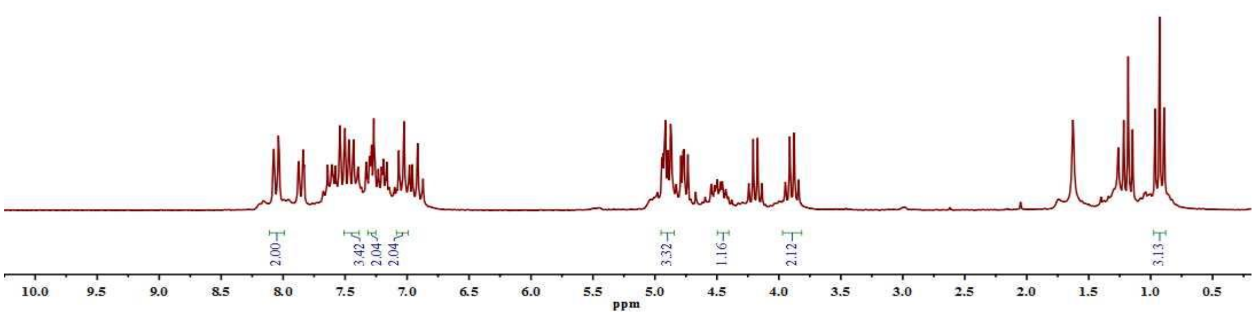

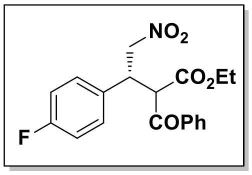

^{1}H NMR Spectrum of Compound **2e** (Major Diastereoisomer) (200 MHz, $CDCl_3$)

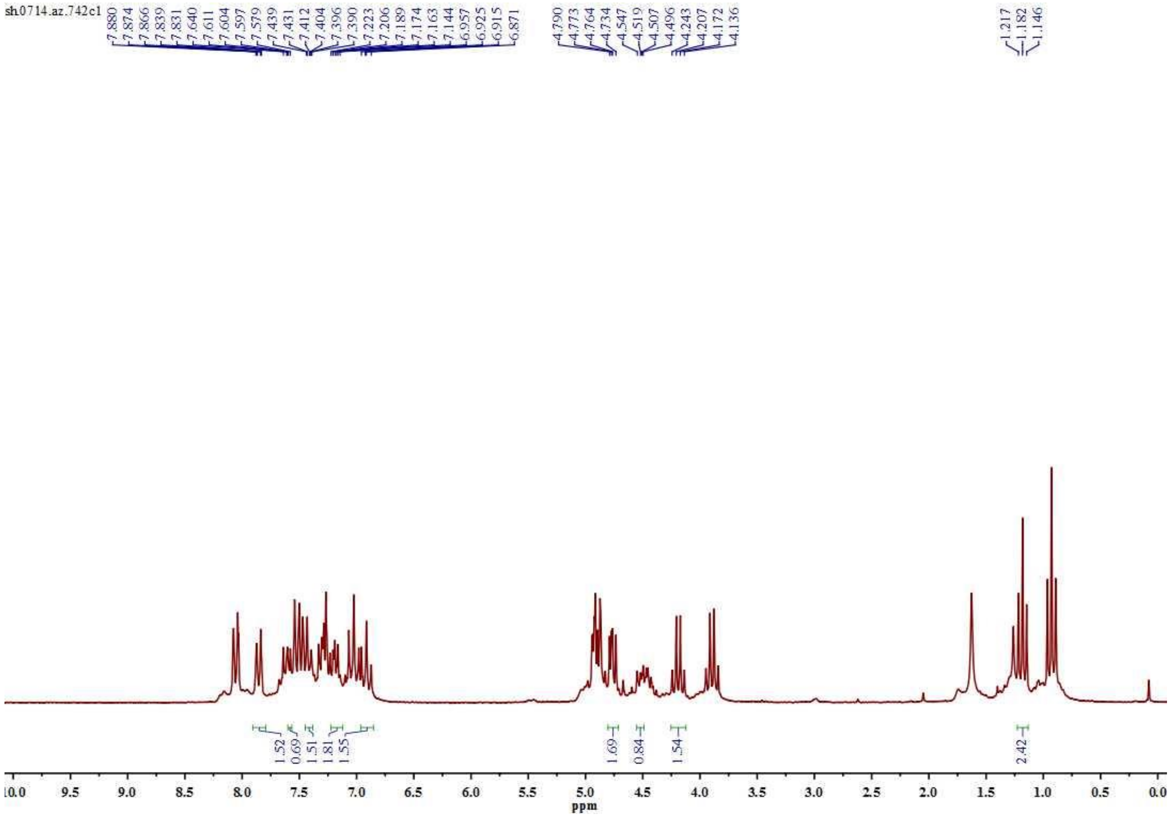

^{1}H NMR Spectrum of Compound **2e** (Minor Diastereoisomer) (200 MHz, $CDCl_3$)

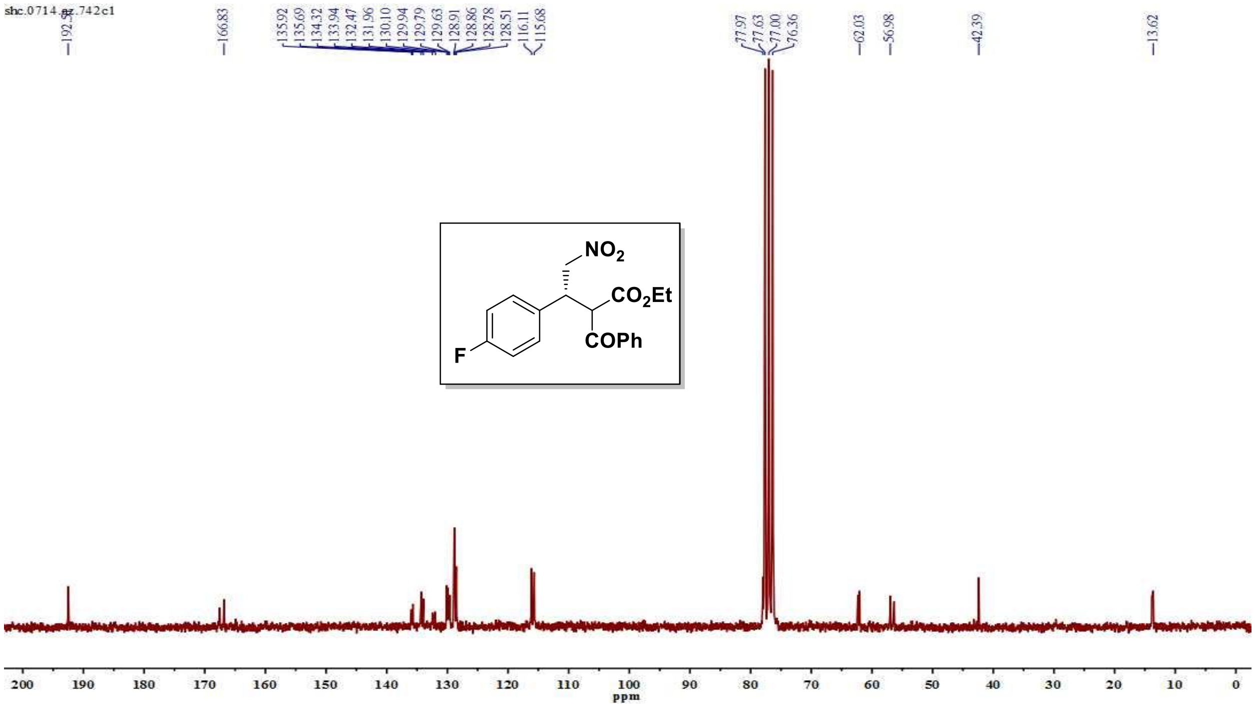

^{13}C NMR Spectrum of Compound **2e** (Major Diastereoisomer) (50 MHz, $CDCl_3$)

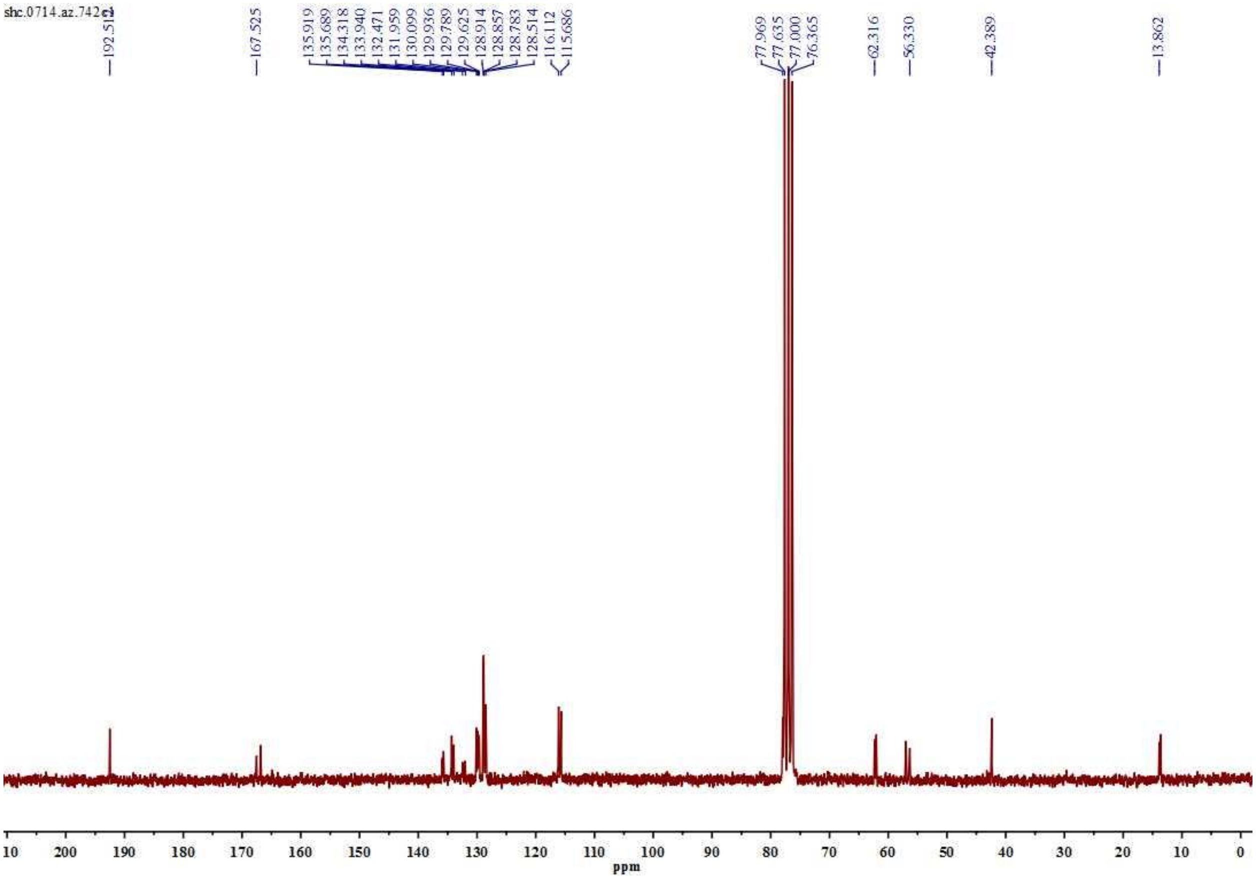

^{13}C NMR Spectrum of Compound **2e** (Minor Diastereoisomer) (50 MHz, $CDCl_3$)

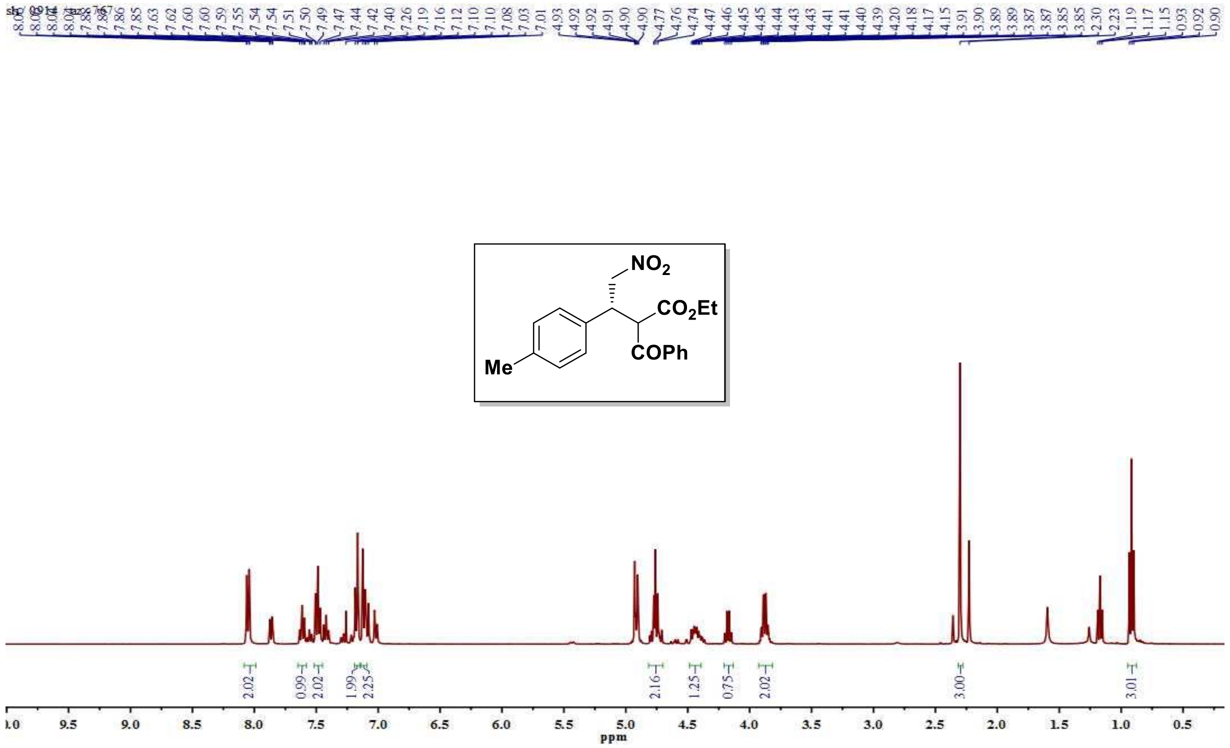

^{1}H NMR Spectrum of Compound **2f** (Major Diastereoisomer) (400 MHz, $CDCl_3$)

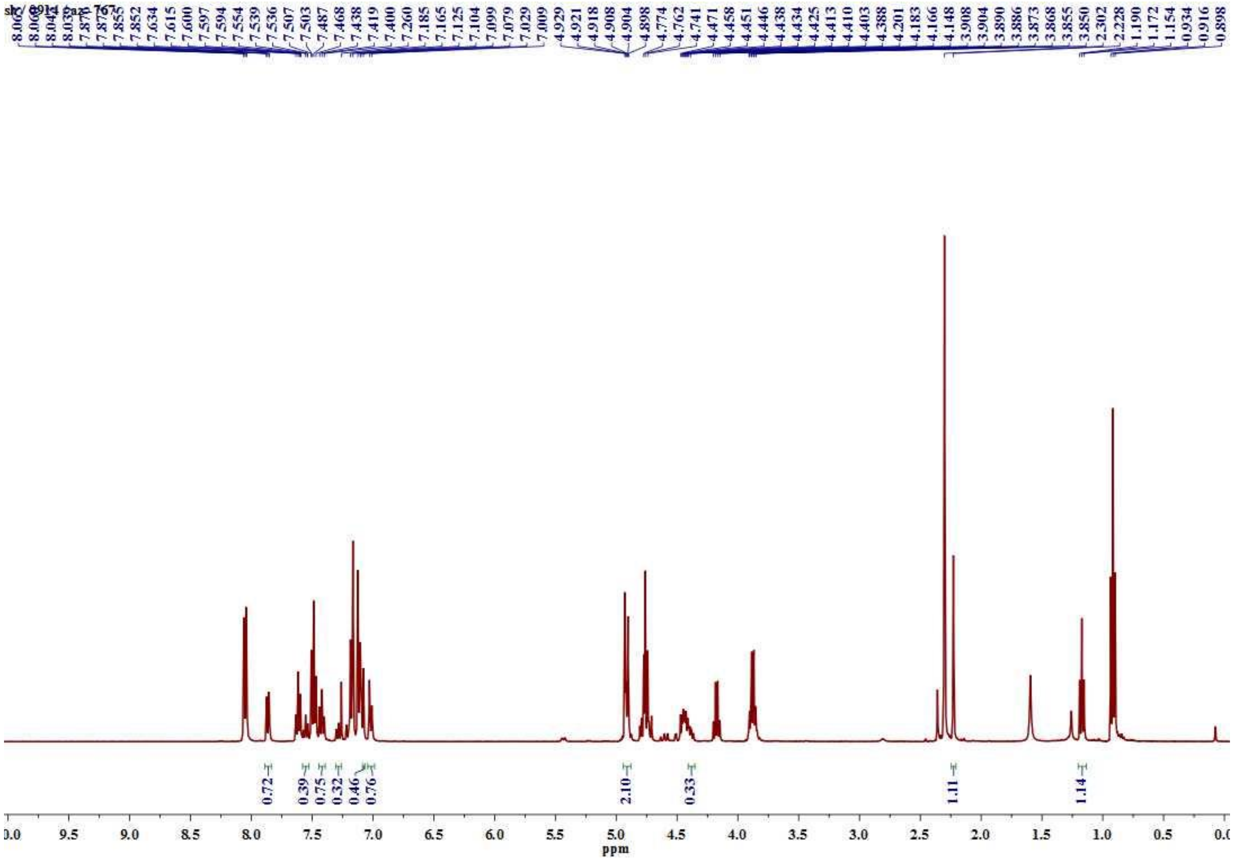

^{1}H NMR Spectrum of Compound **2f** (Minor Diastereoisomer) (400 MHz, $CDCl_3$)

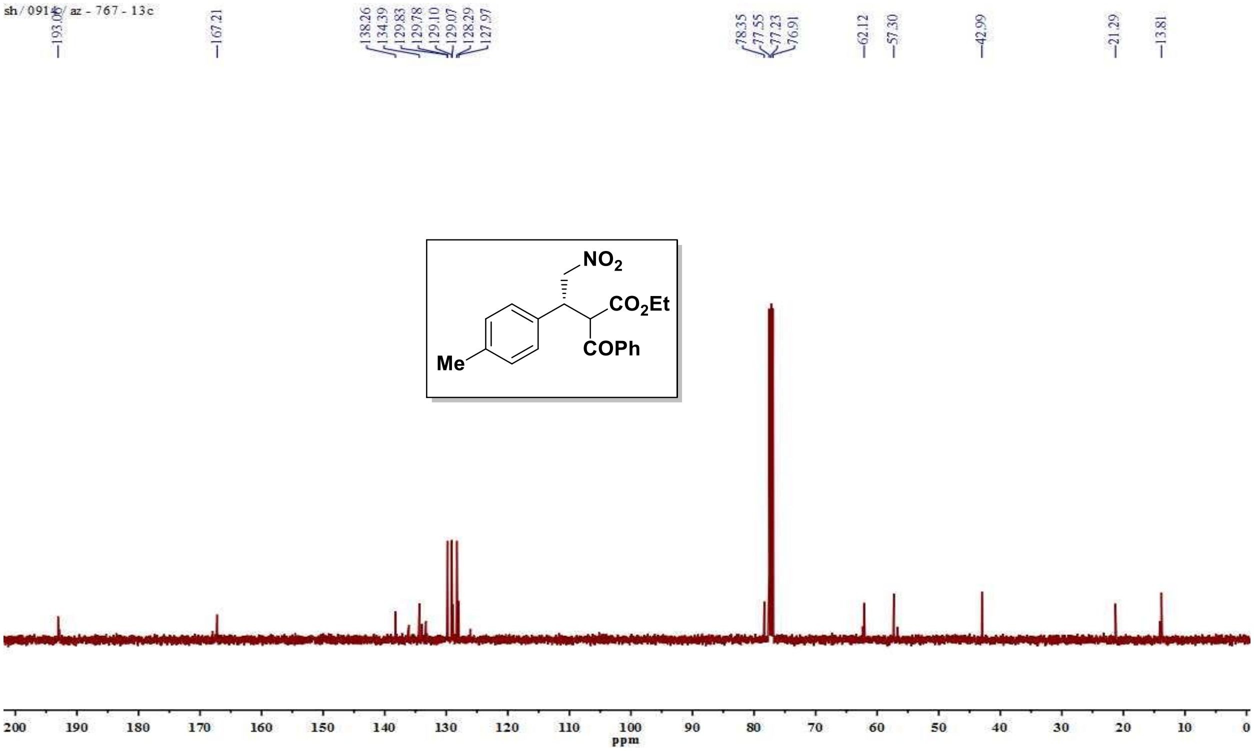

^{13}C NMR Spectrum of Compound **2f** (Major Diastereoisomer) (100 MHz, $CDCl_3$)

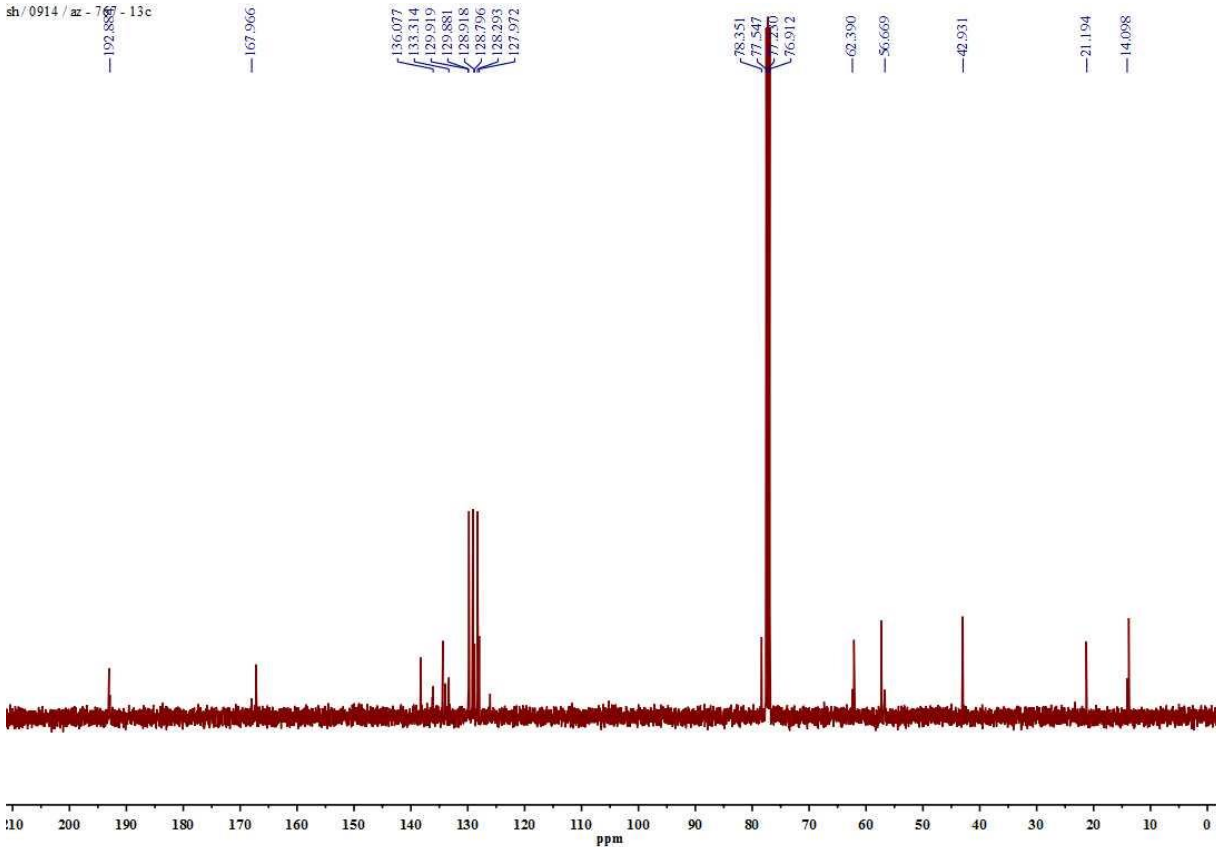

^{13}C NMR Spectrum of Compound **2f** (Minor Diastereoisomer) (100 MHz, $CDCl_3$)

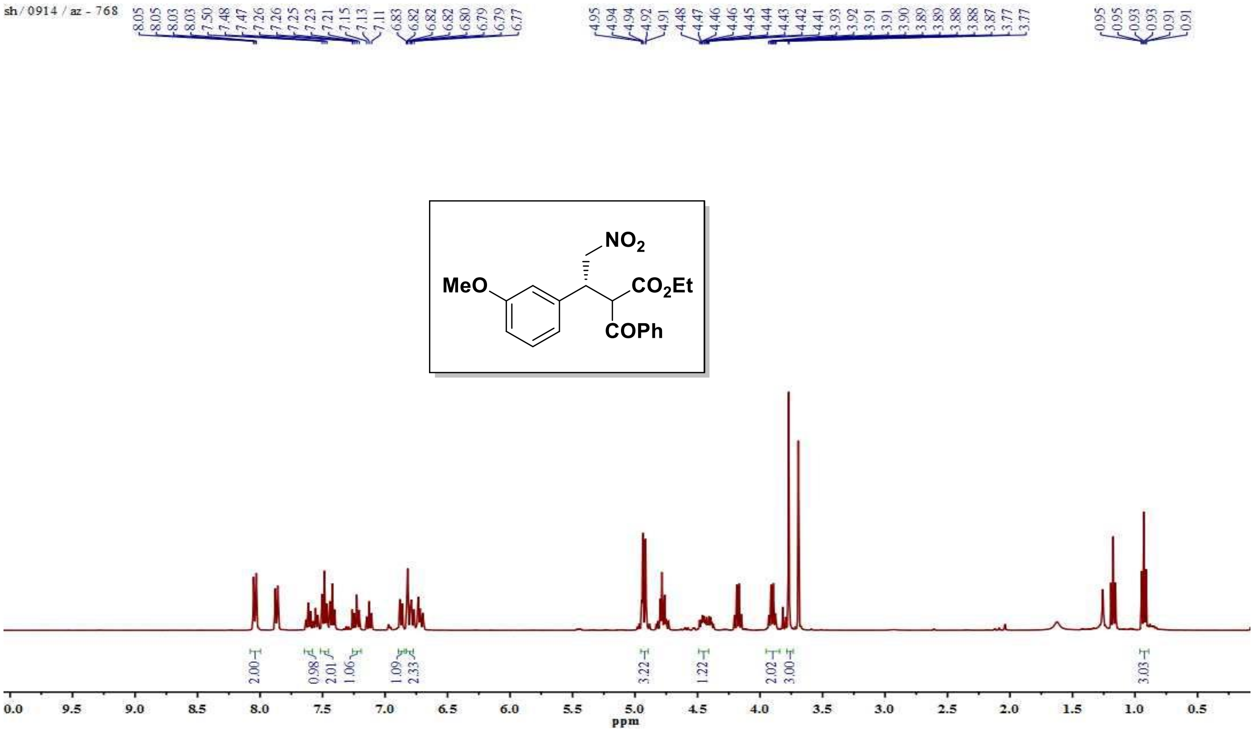

^{1}H NMR Spectrum of Compound **2g** (Major Diastereoisomer) (400 MHz, $CDCl_3$)

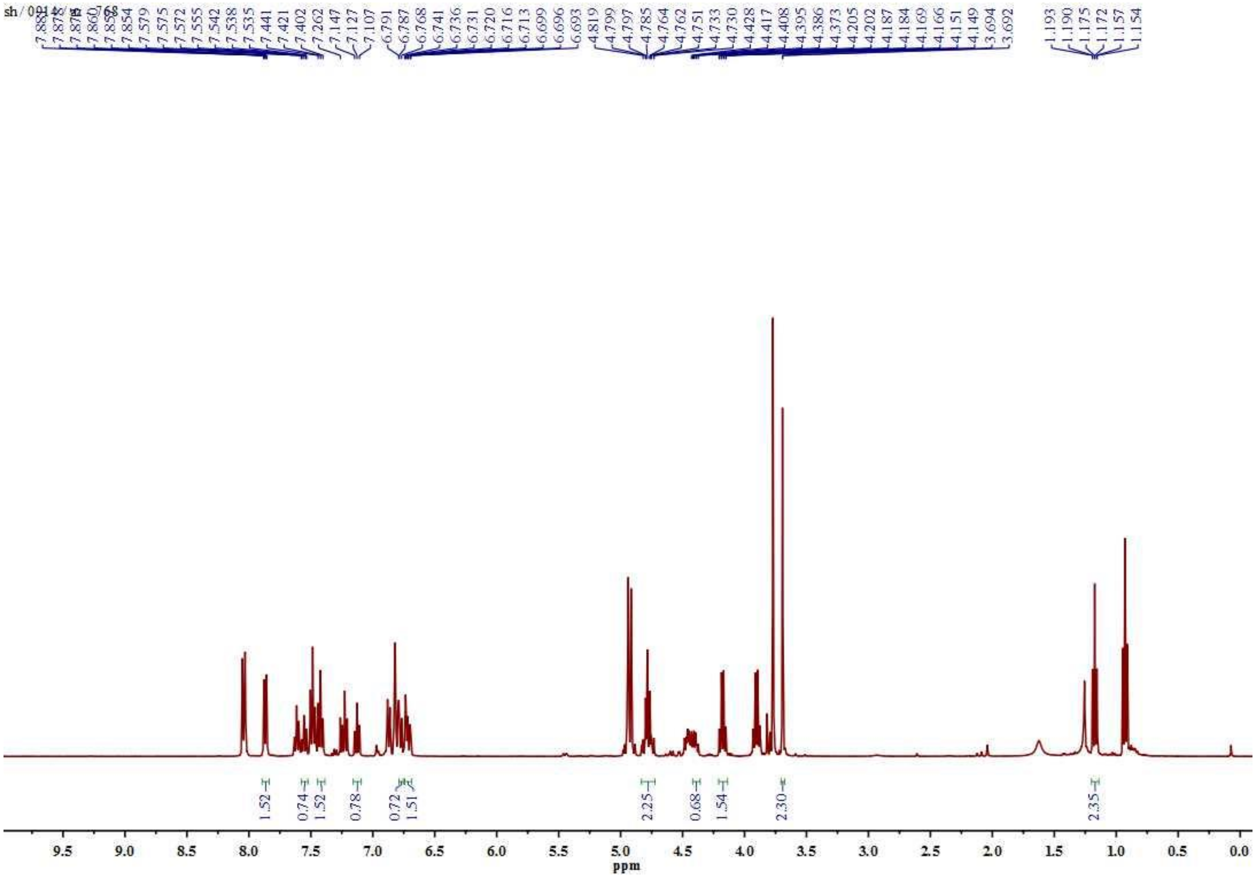

^{1}H NMR Spectrum of Compound **2g** (Minor Diastereoisomer) (400 MHz, $CDCl_3$)

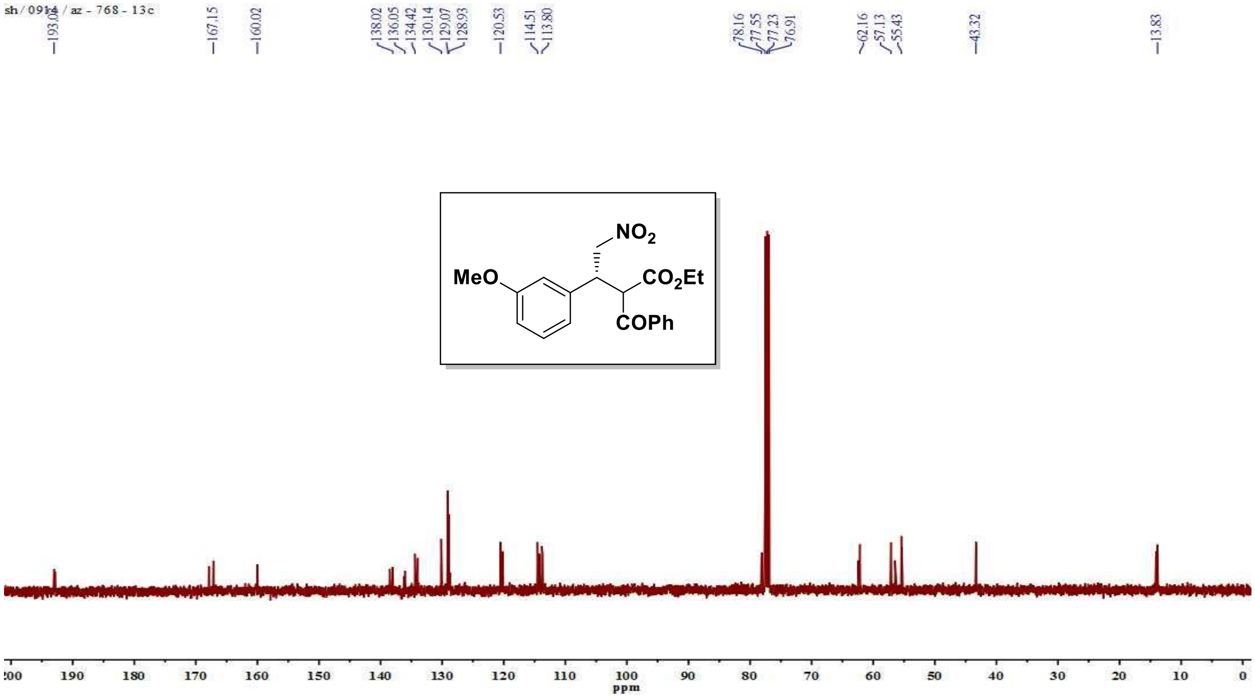

^{13}C NMR Spectrum of Compound **2g** (Major Diastereoisomer) (100 MHz, $CDCl_3$)

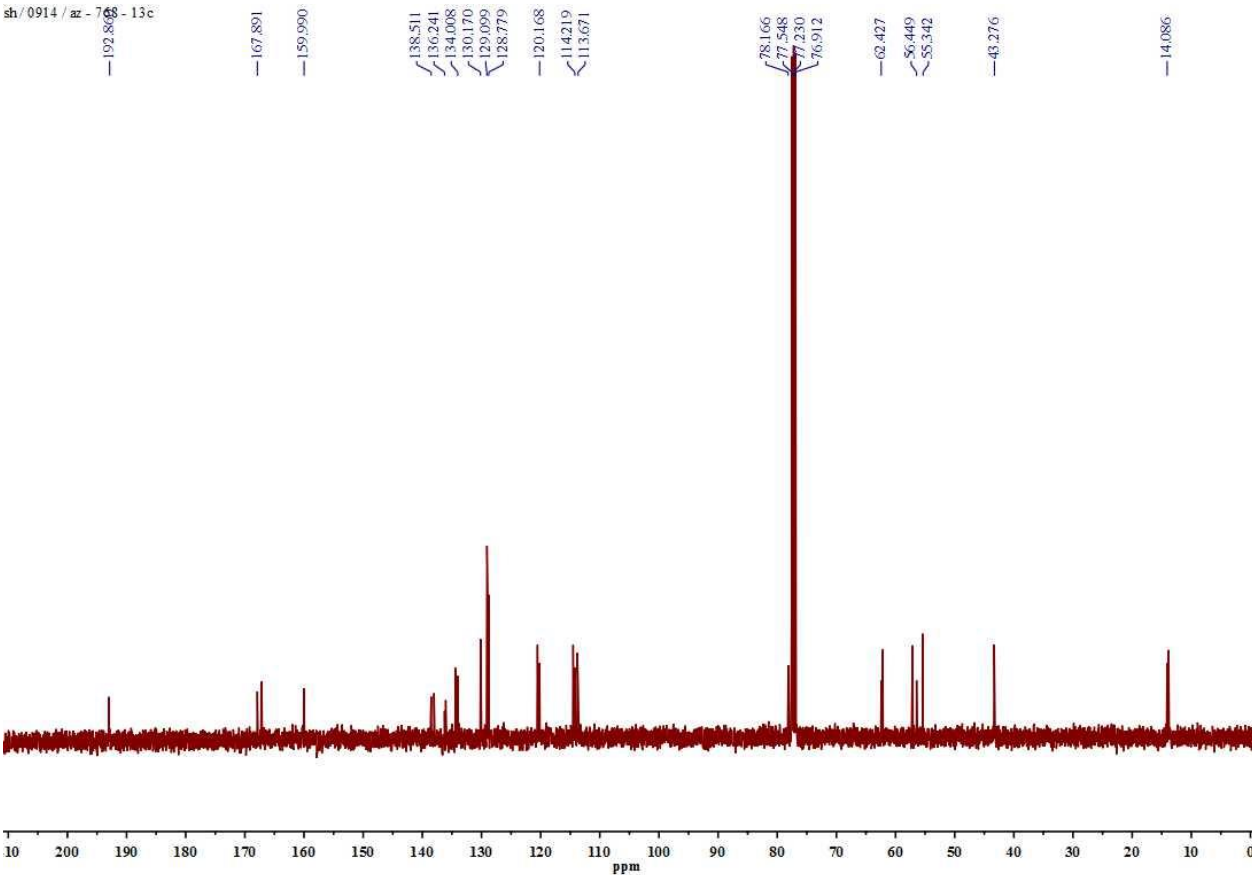

^{13}C NMR Spectrum of Compound **2g** (Minor Diastereoisomer) (100 MHz, $CDCl_3$)

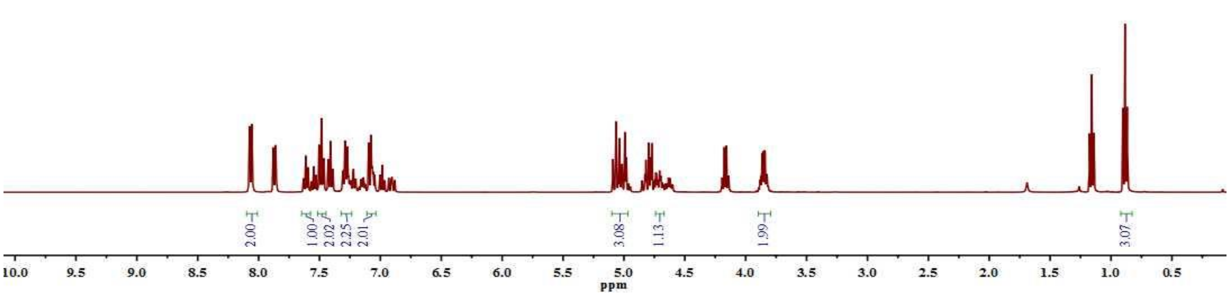

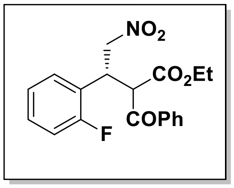

^{1}H NMR Spectrum of Compound **2h** (Major Diastereoisomer) (400 MHz, $CDCl_3$)

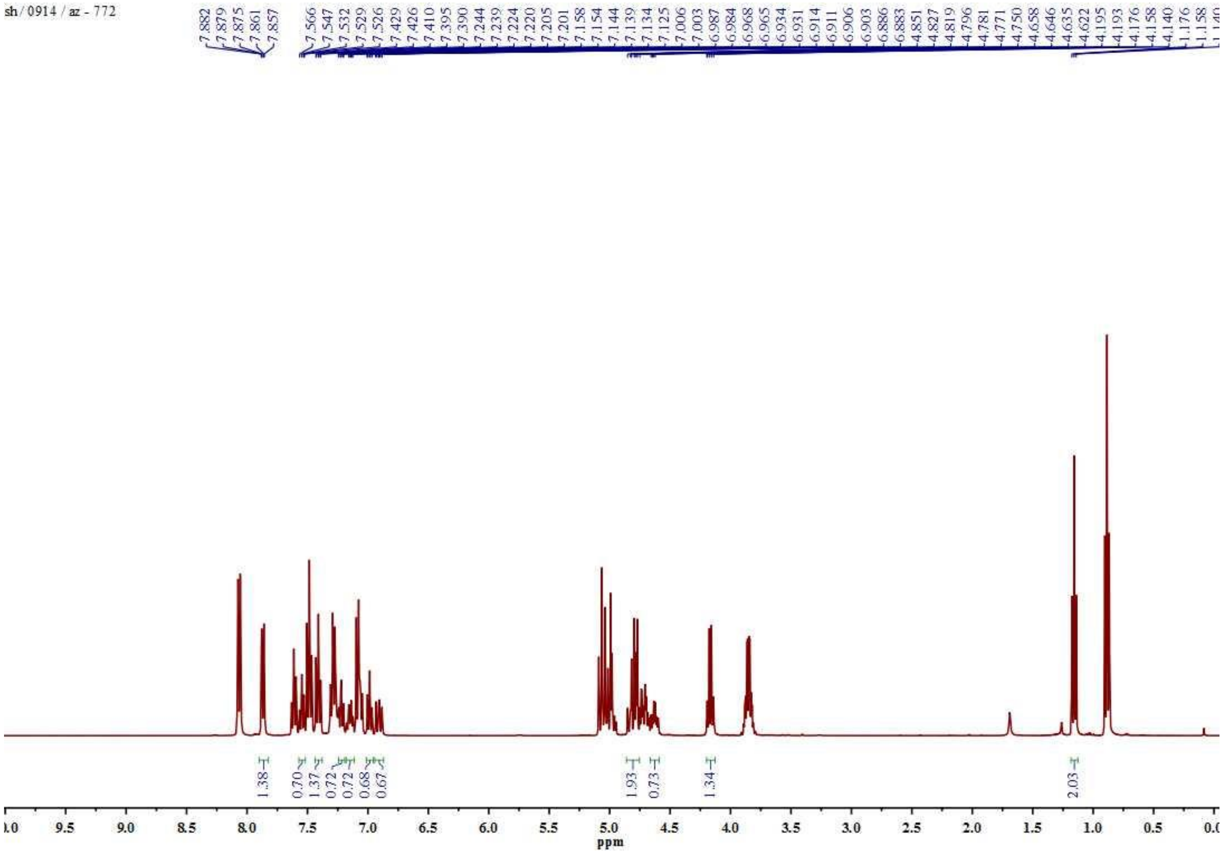

^{1}H NMR Spectrum of Compound **2h** (Minor Diastereoisomer) (400 MHz, $CDCl_3$)

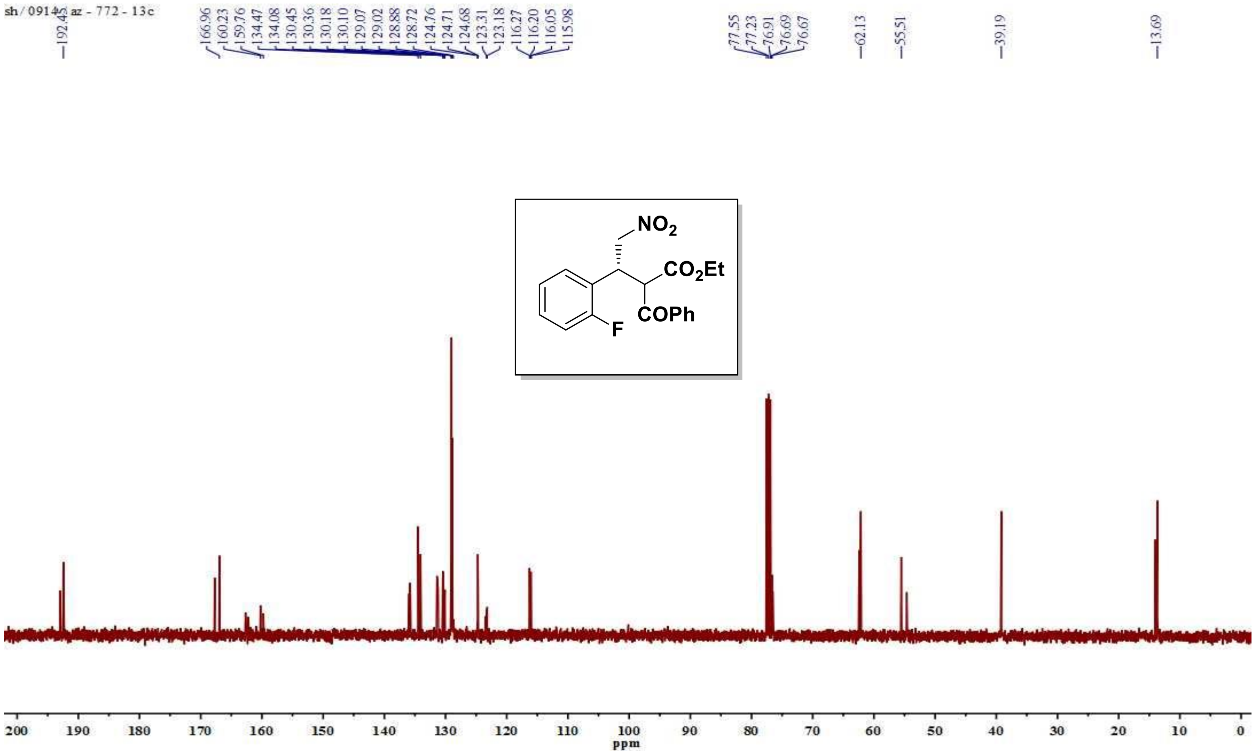

^{13}C NMR Spectrum of Compound **2h** (Major Diastereoisomer) (100 MHz, $CDCl_3$)

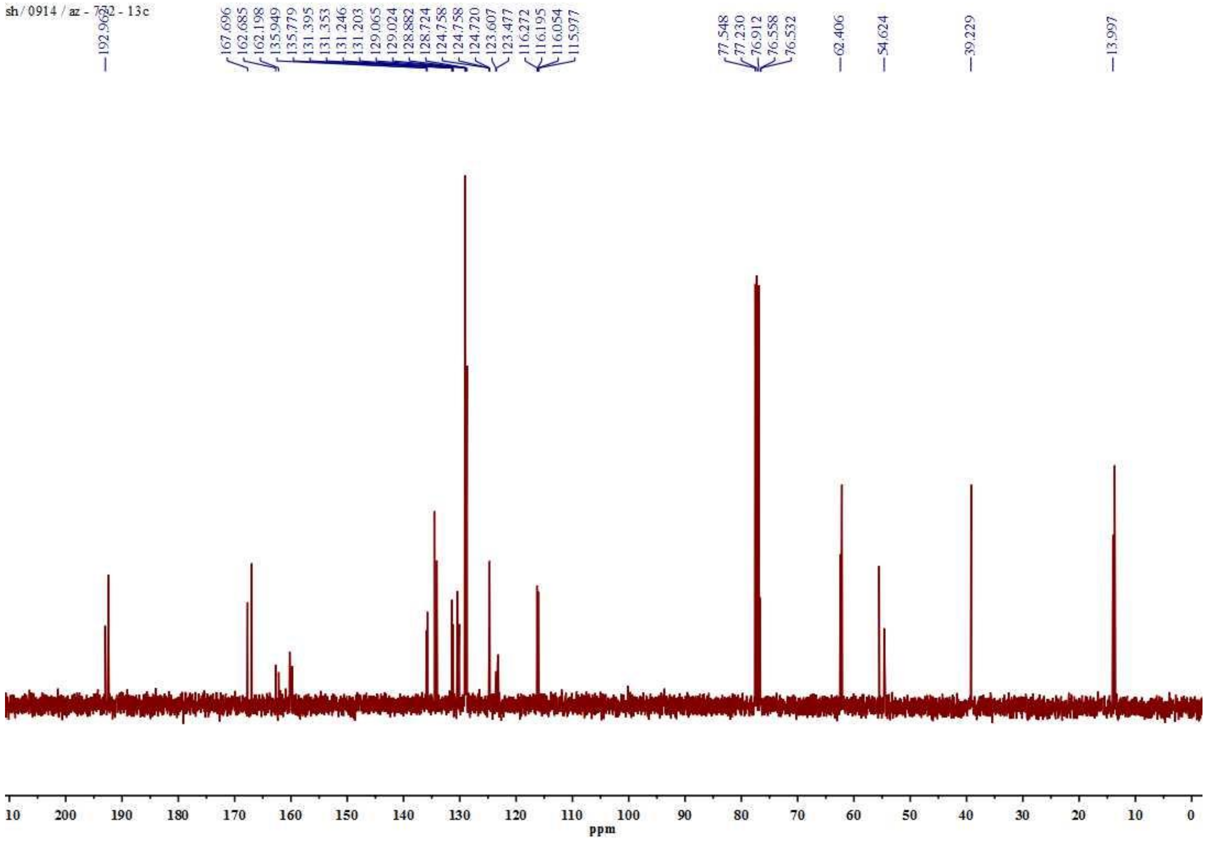

^{13}C NMR Spectrum of Compound **2h** (Minor Diastereoisomer) (100 MHz, $CDCl_3$)

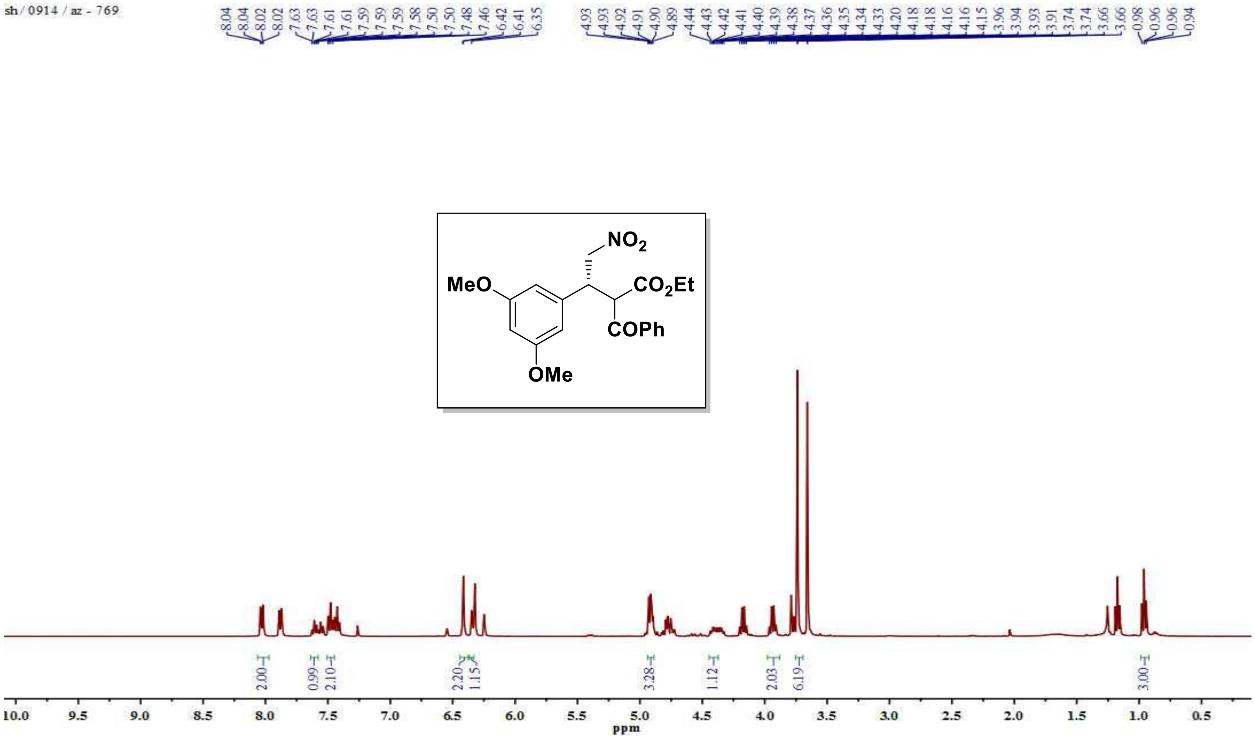

^{1}H NMR Spectrum of Compound **2i** (Major Diastereoisomer) (400 MHz, $CDCl_3$)

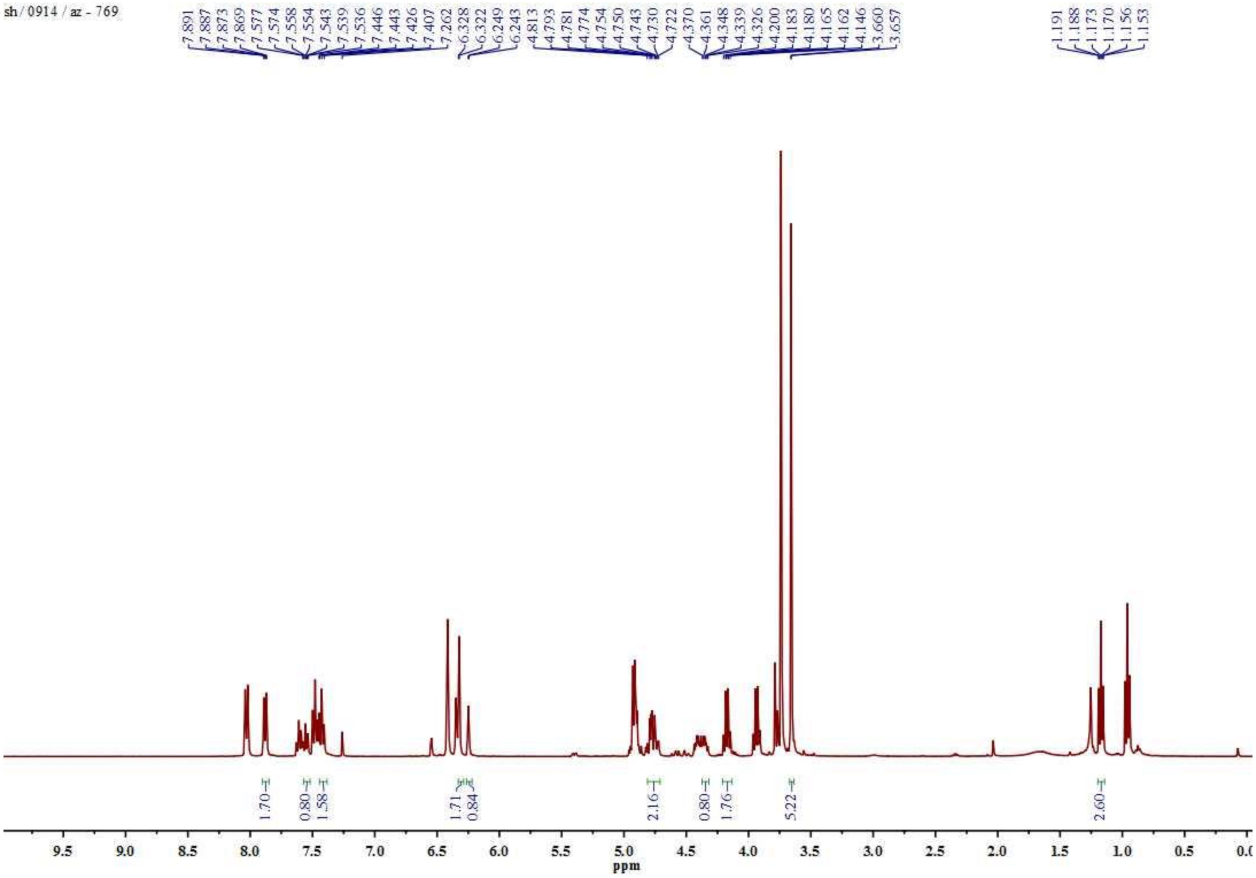

^{1}H NMR Spectrum of Compound **2i** (Minor Diastereoisomer) (400 MHz, $CDCl_3$)

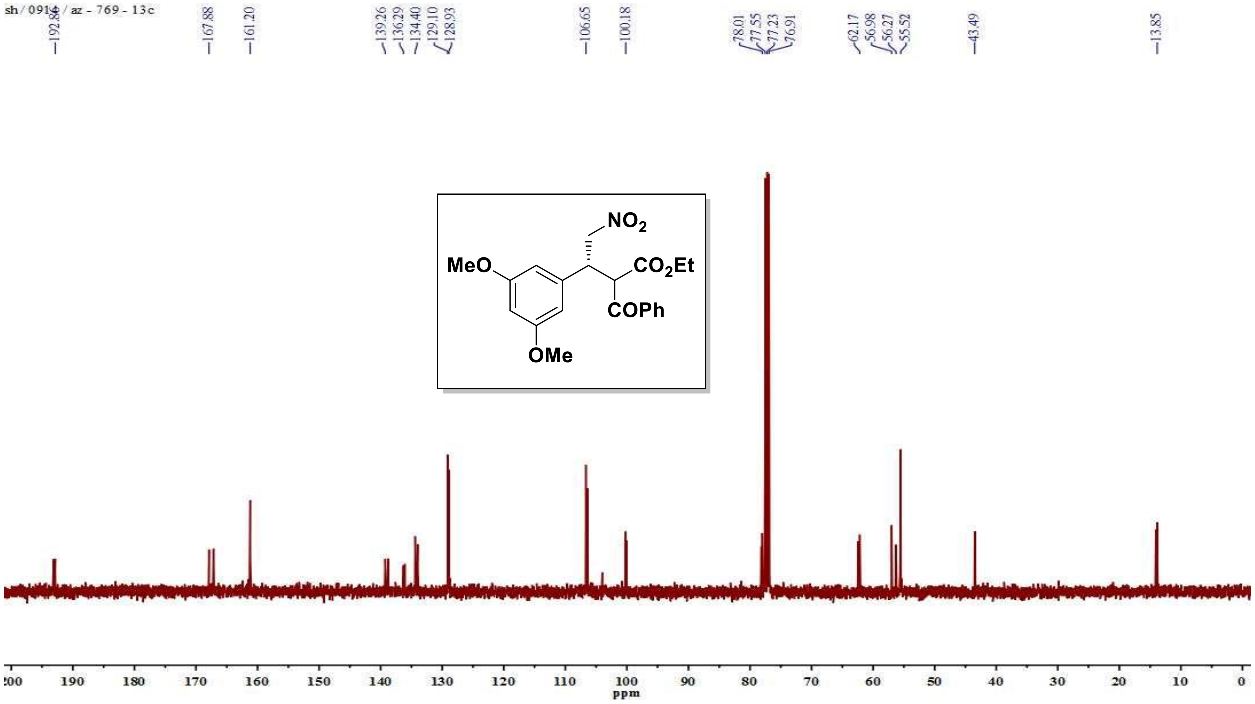

^{13}C NMR Spectrum of Compound **2i** (100 MHz, $CDCl_3$)

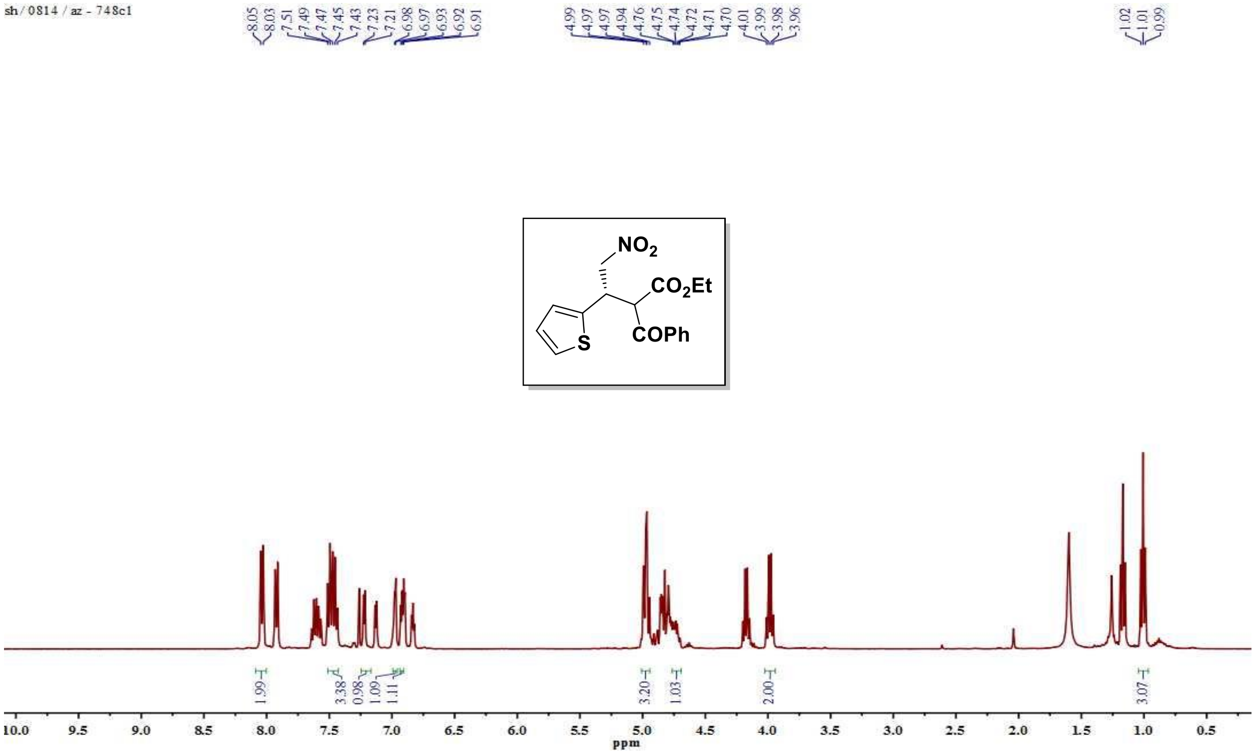

^{1}H NMR Spectrum of Compound **2j** (Major Diastereoisomer) (400 MHz, $CDCl_3$)

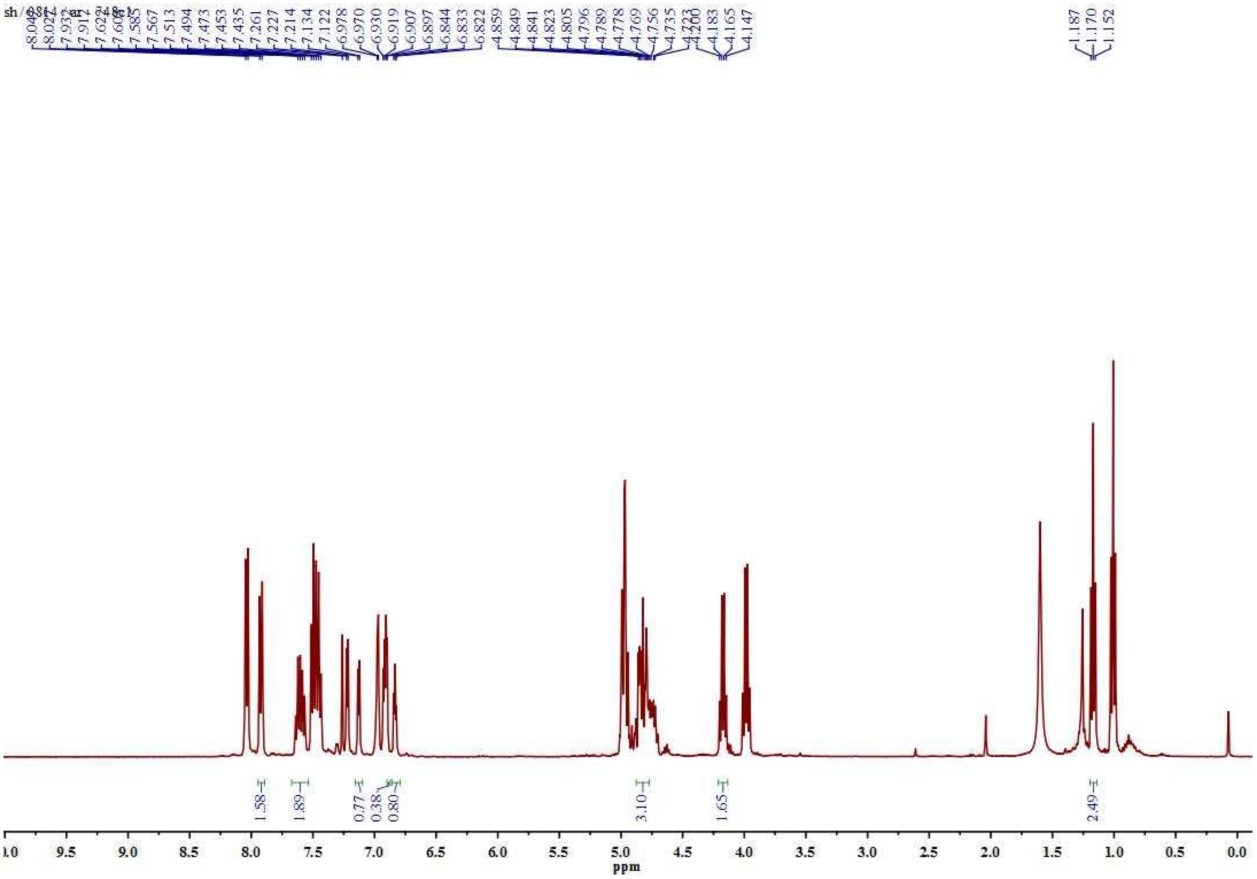

^{1}H NMR Spectrum of Compound **2j** (Minor Diastereoisomer) (400 MHz, $CDCl_3$)

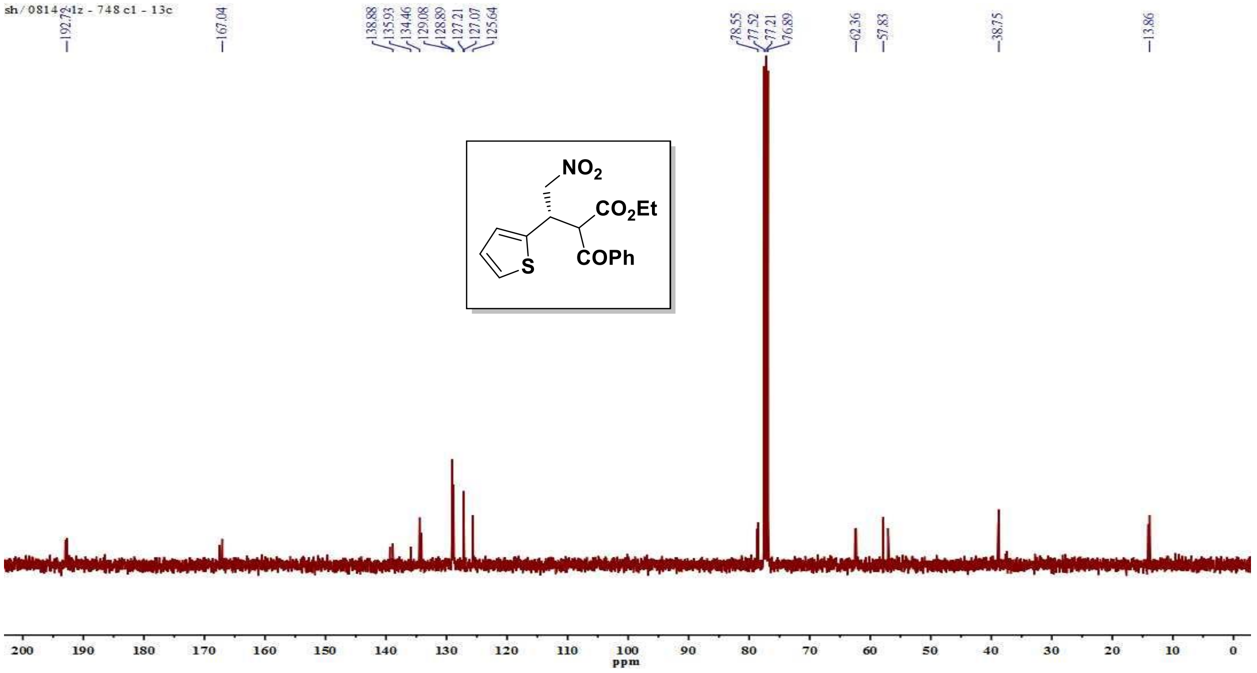

^{13}C NMR Spectrum of Compound **2j** (Major Diastereoisomer) (100 MHz, $CDCl_3$)

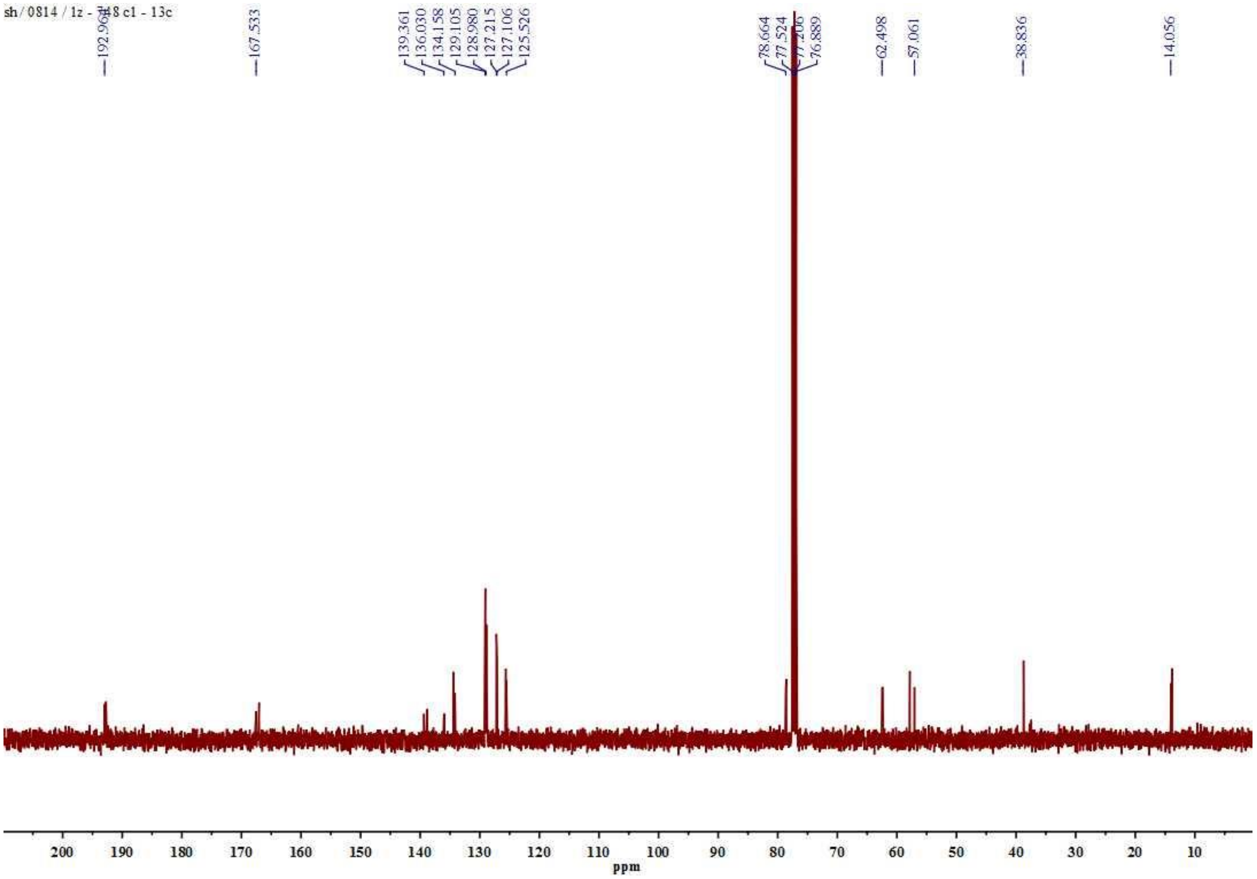

^{13}C NMR Spectrum of Compound **2j** (Minor Diastereoisomer) (100 MHz, $CDCl_3$)

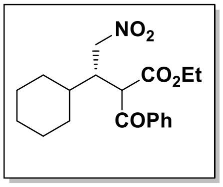

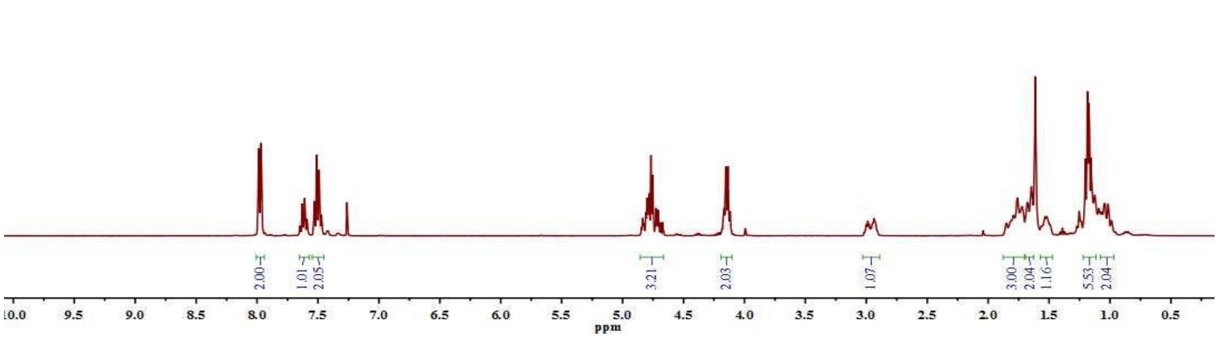

^{1}H NMR Spectrum of Compound **2k** (400 MHz, $CDCl_3$)

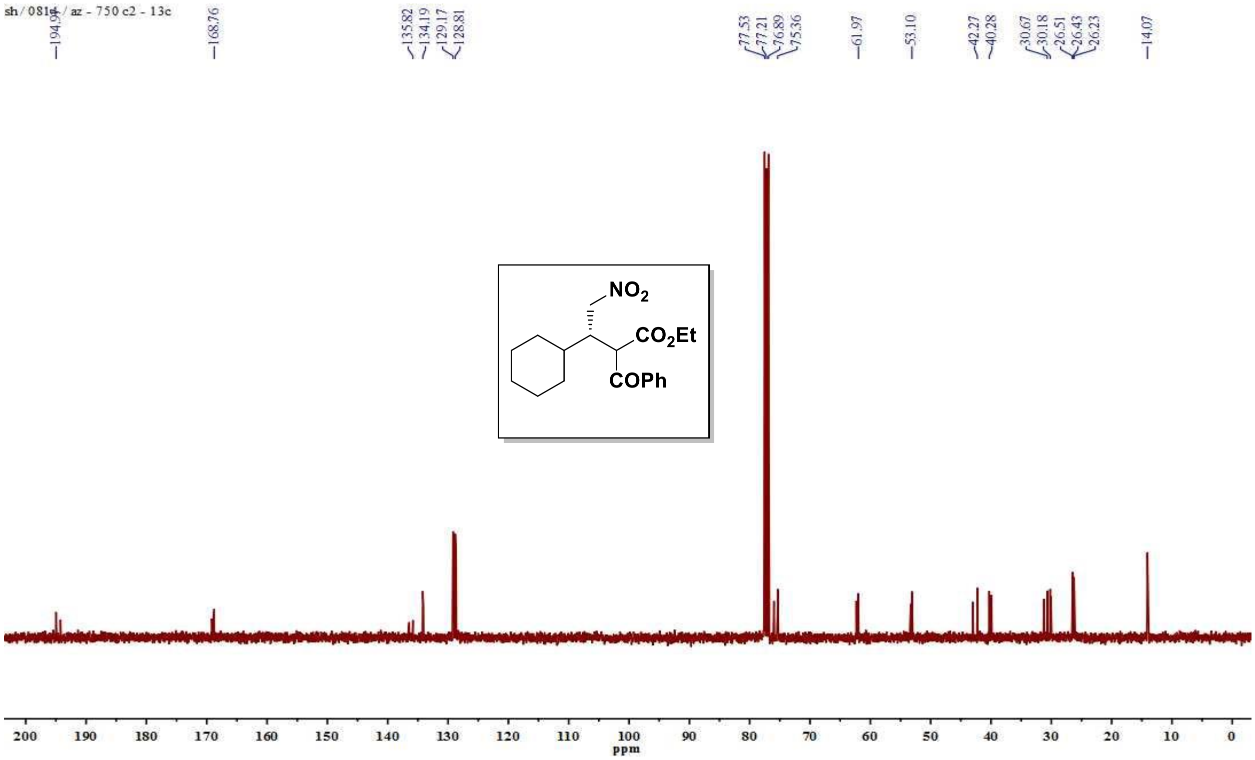

^{13}C NMR Spectrum of Compound **2k** (100 MHz, $CDCl_3$)

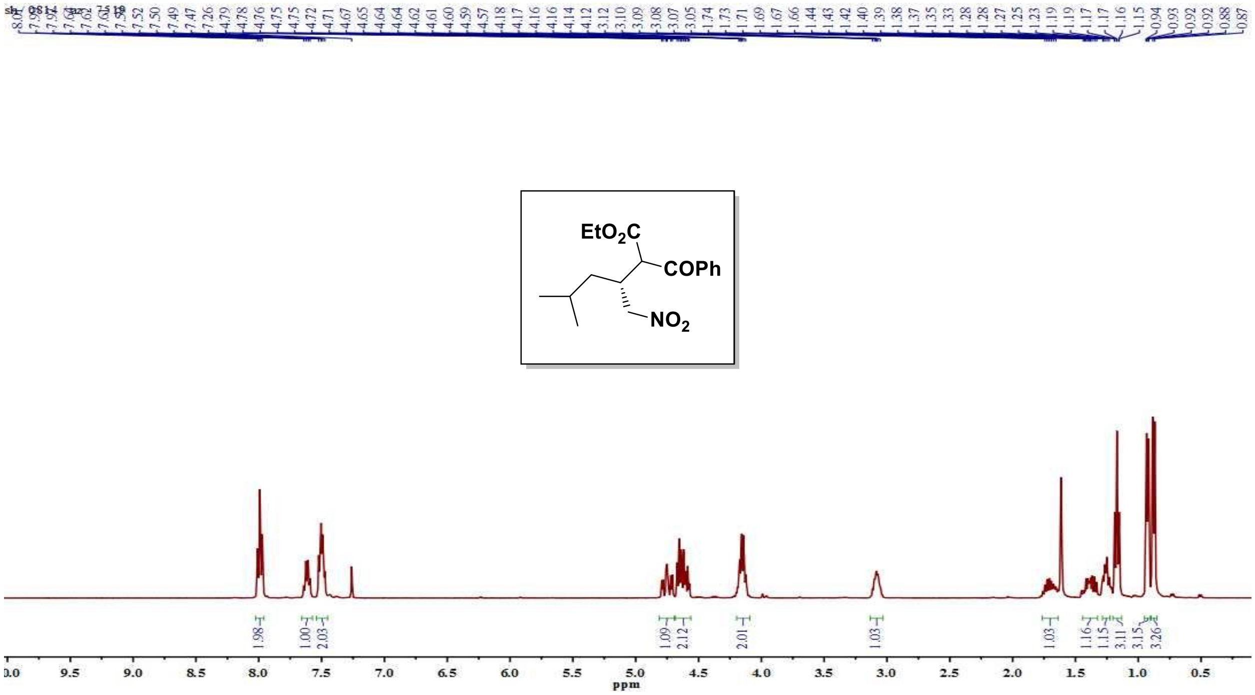

^{1}H NMR Spectrum of Compound **2l** (400 MHz, $CDCl_3$)

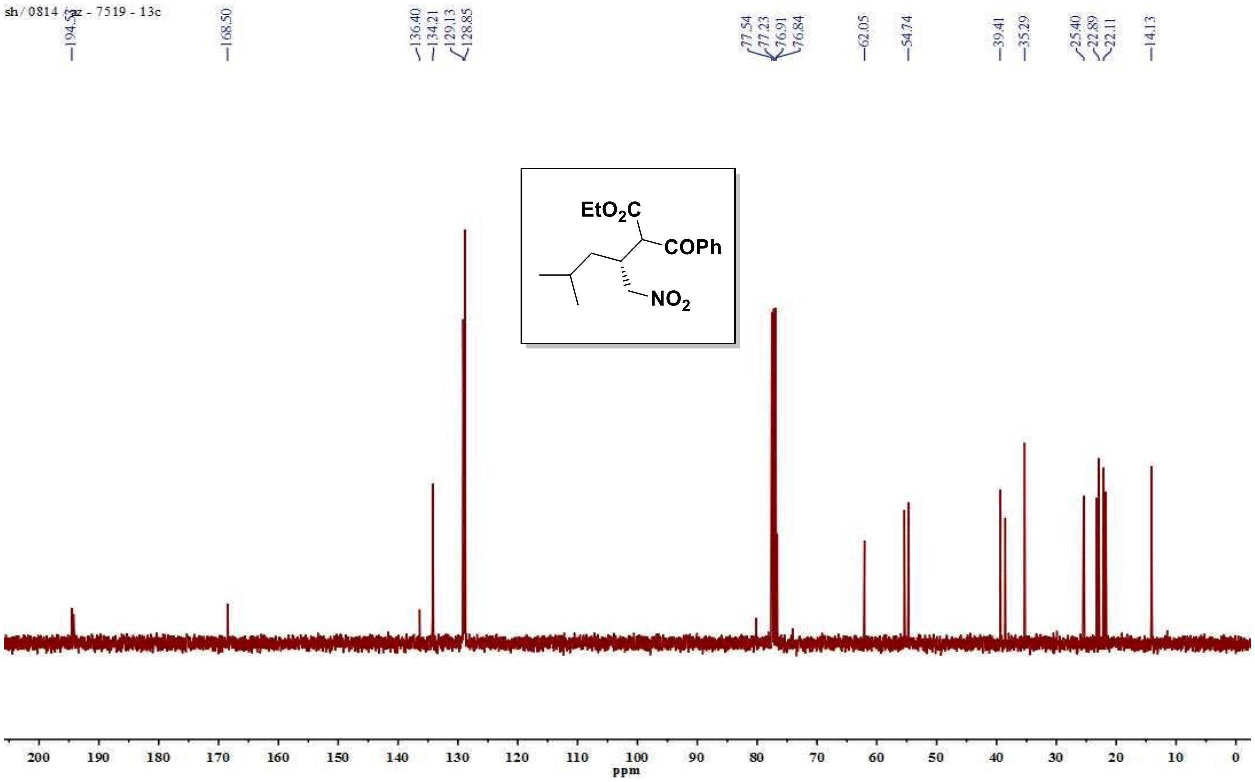

^{13}C NMR Spectrum of Compound **2l** (100 MHz, $CDCl_3$)

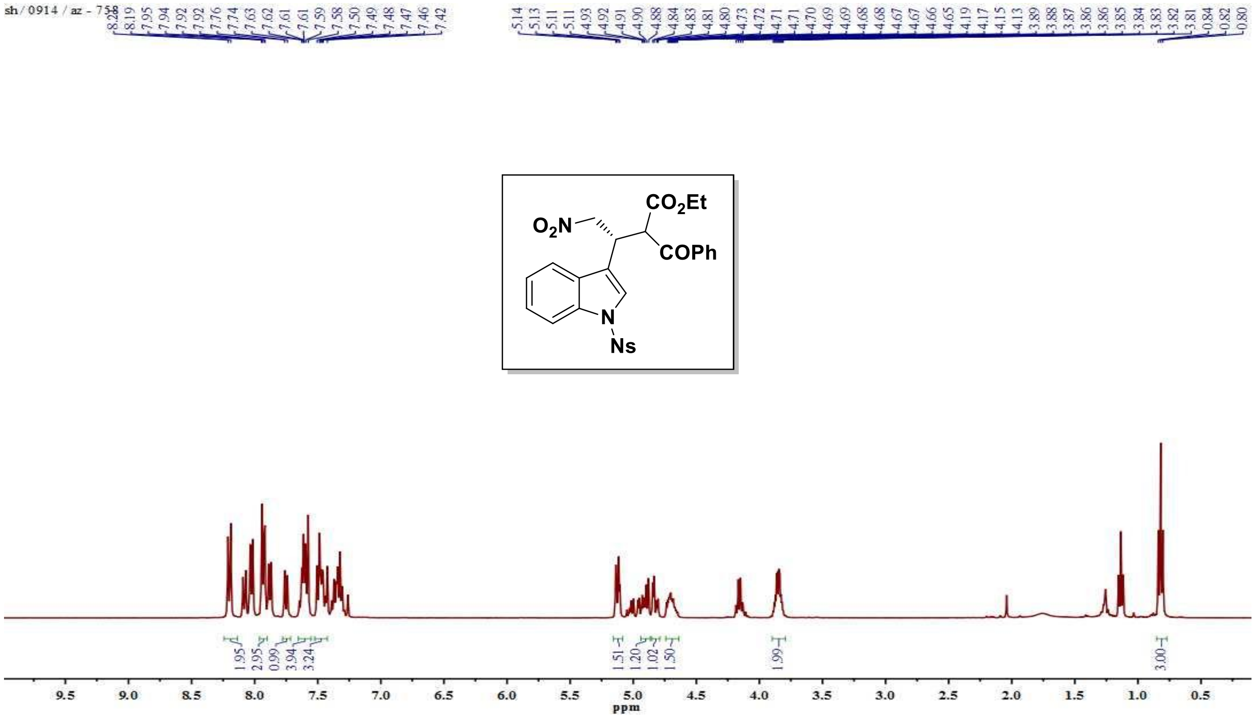

^{1}H NMR Spectrum of Compound **2m** (Major Diastereoisomer) (400 MHz, $CDCl_3$)

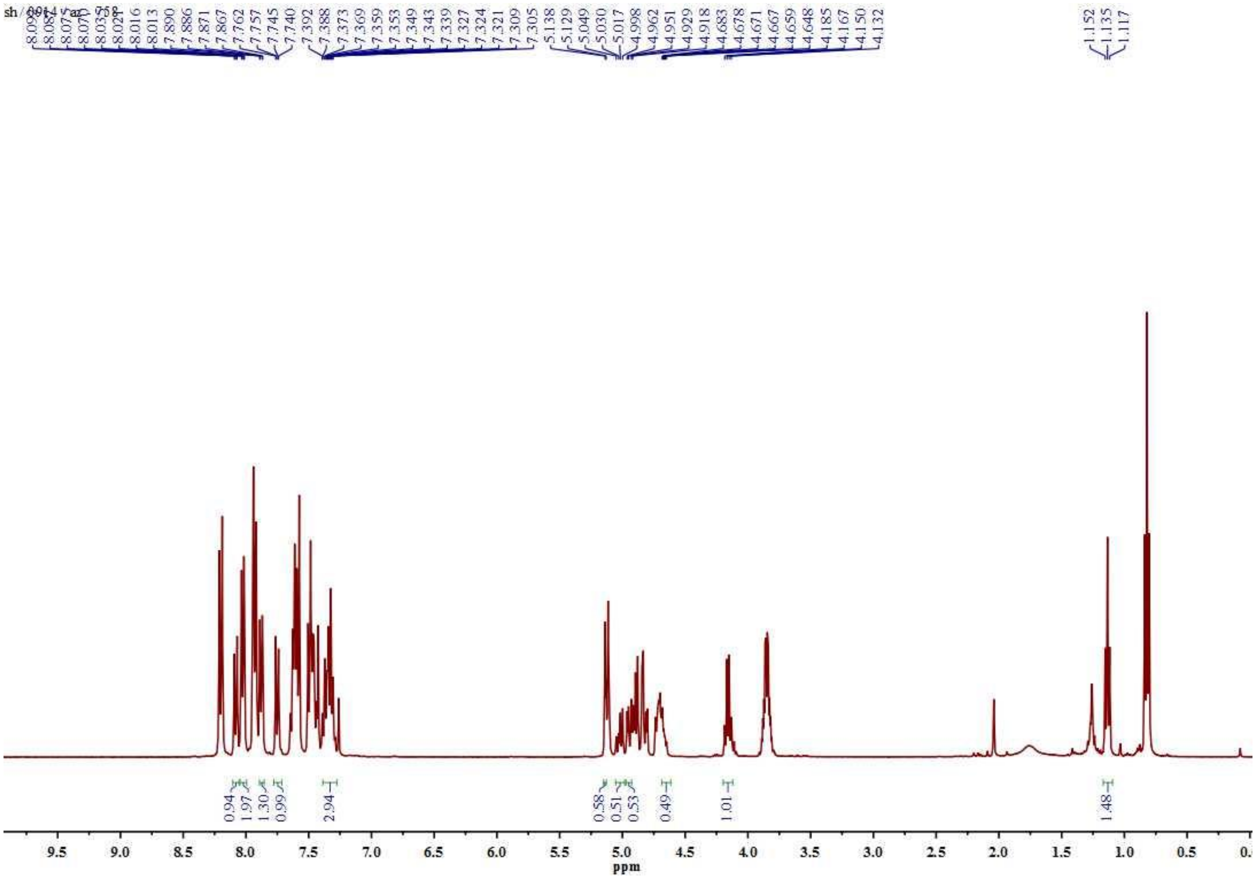

^{1}H NMR Spectrum of Compound **2m** (Minor Diastereoisomer) (400 MHz, $CDCl_3$)

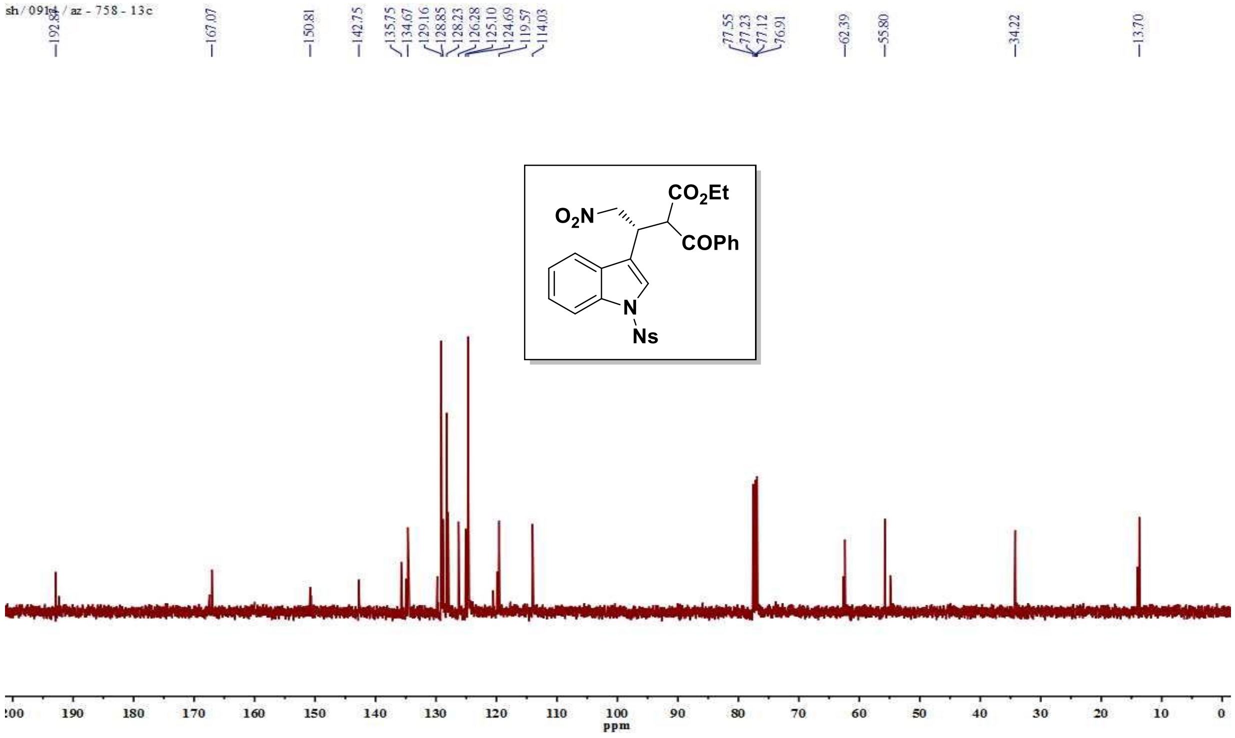

^{13}C NMR Spectrum of Compound **2m** (Major Diastereoisomer) (100 MHz, $CDCl_3$)

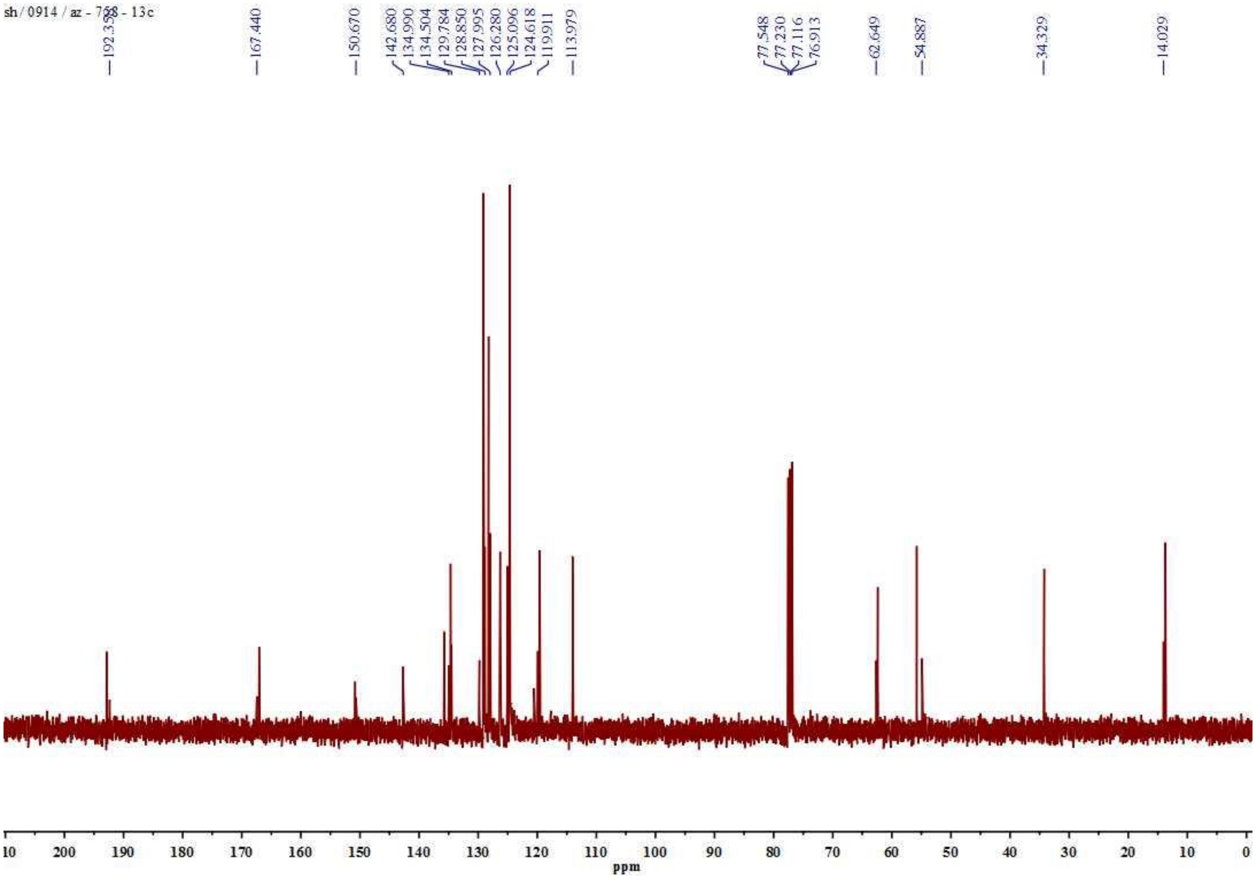

^{13}C NMR Spectrum of Compound **2m** (Minor Diastereoisomer) (100 MHz, $CDCl_3$)

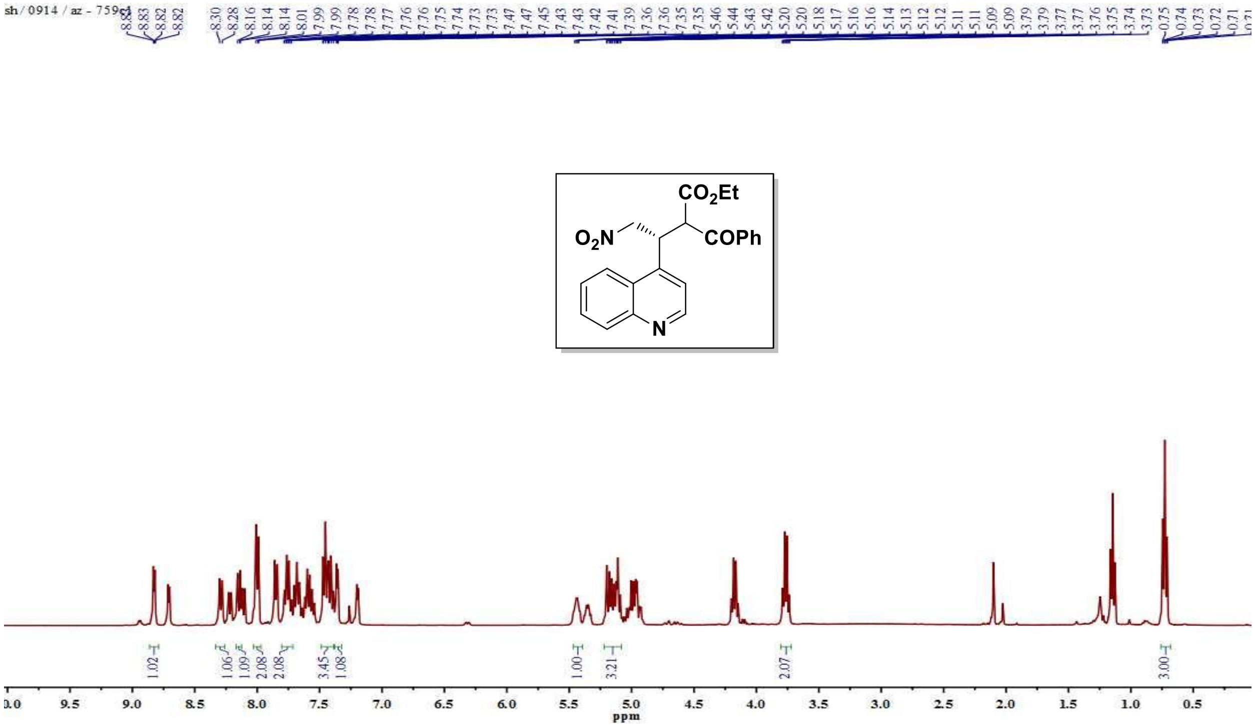

^{1}H NMR Spectrum of Compound **2n** (Major Diastereoisomer) (400 MHz, $CDCl_3$)

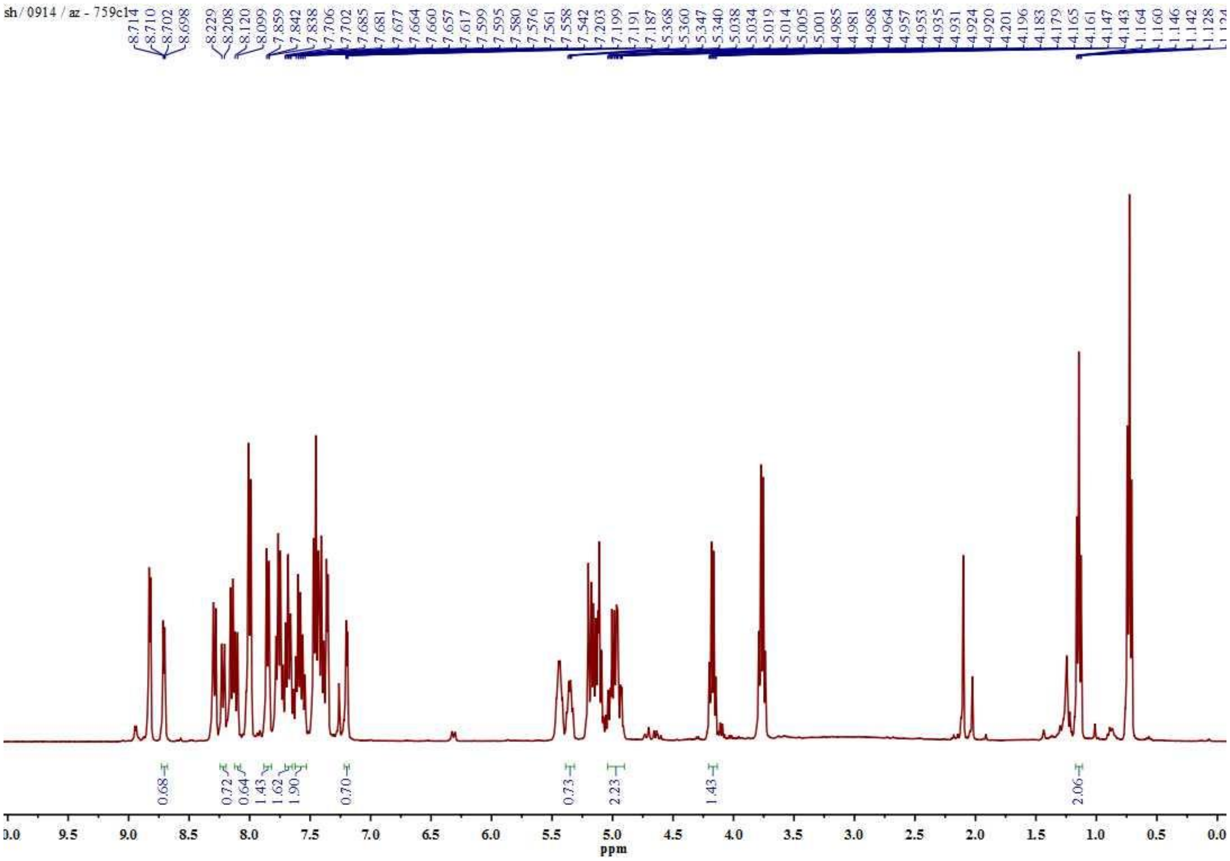

^{1}H NMR Spectrum of Compound **2n** (Minor Diastereoisomer) (400 MHz, $CDCl_3$)

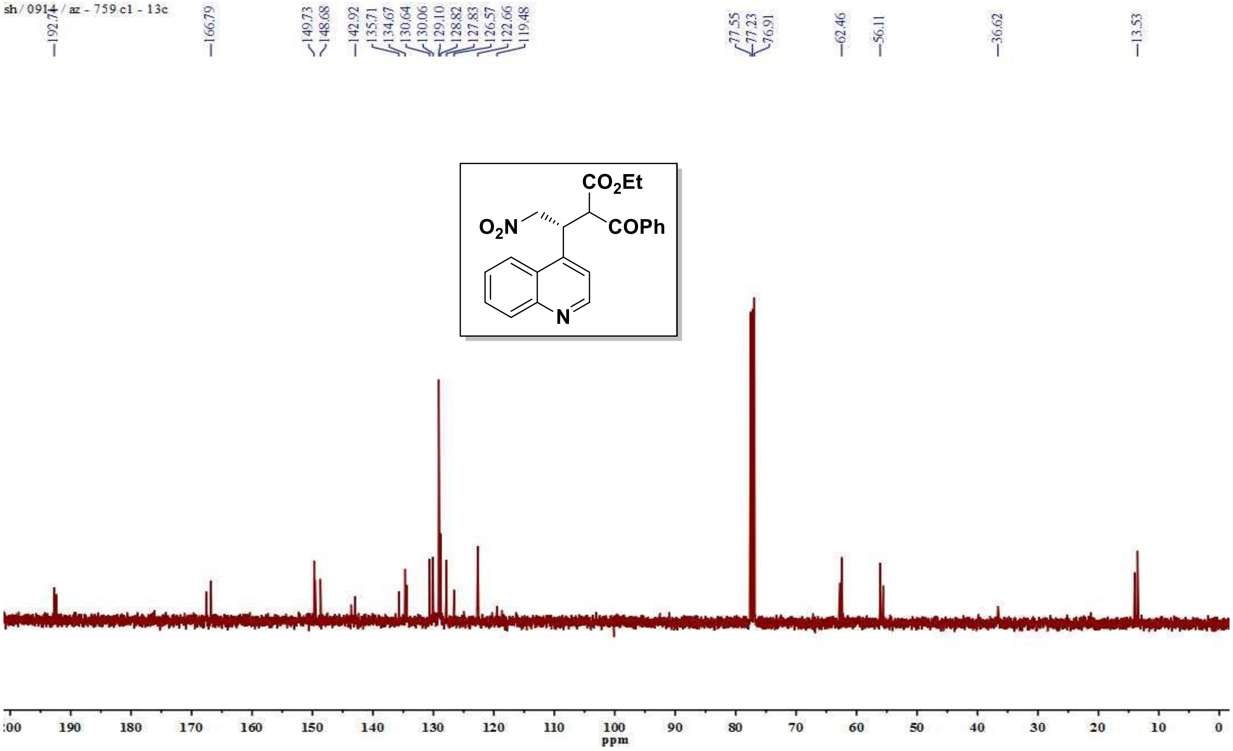

^{13}C NMR Spectrum of Compound **2n** (Major Diastereoisomer) (100 MHz, $CDCl_3$)

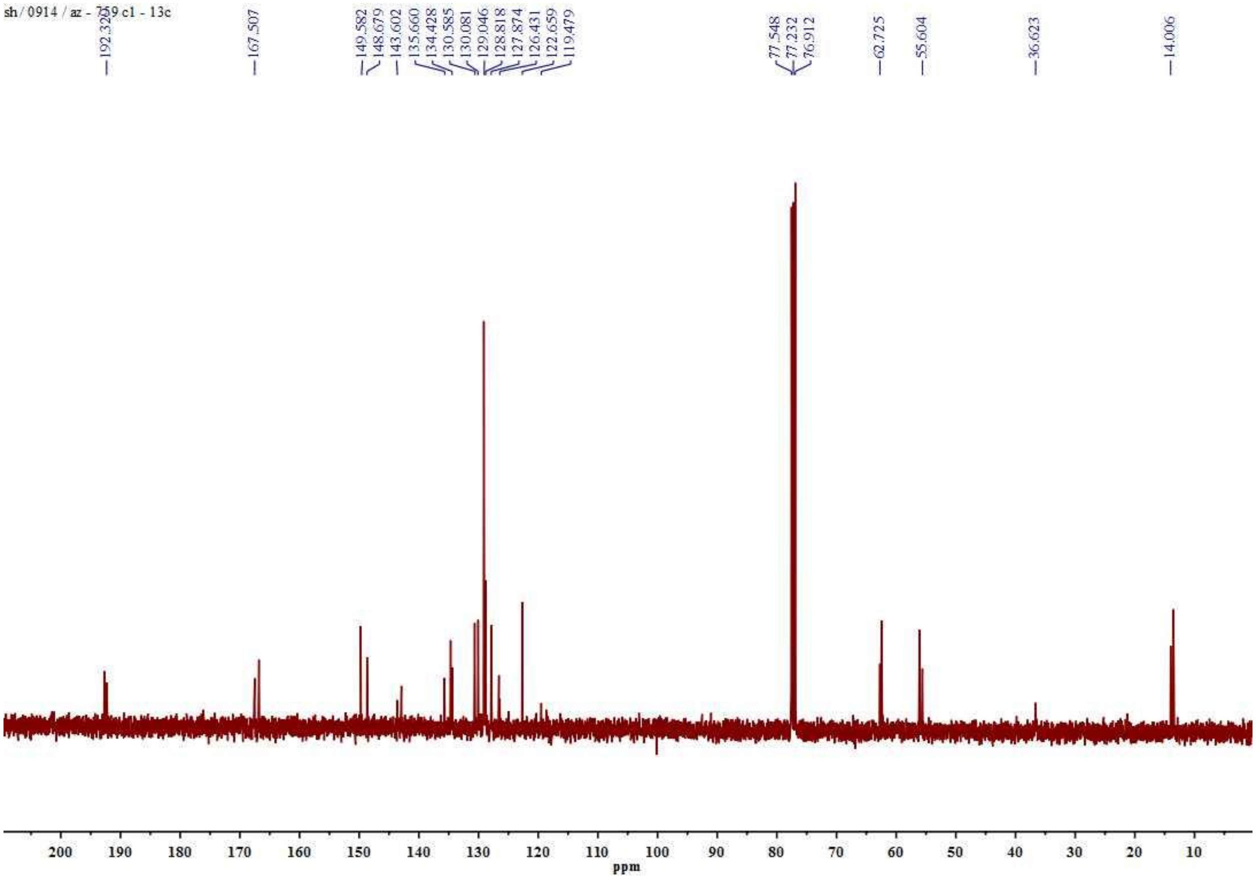

^{13}C NMR Spectrum of Compound **2n** (Minor Diastereoisomer) (100 MHz, $CDCl_3$)

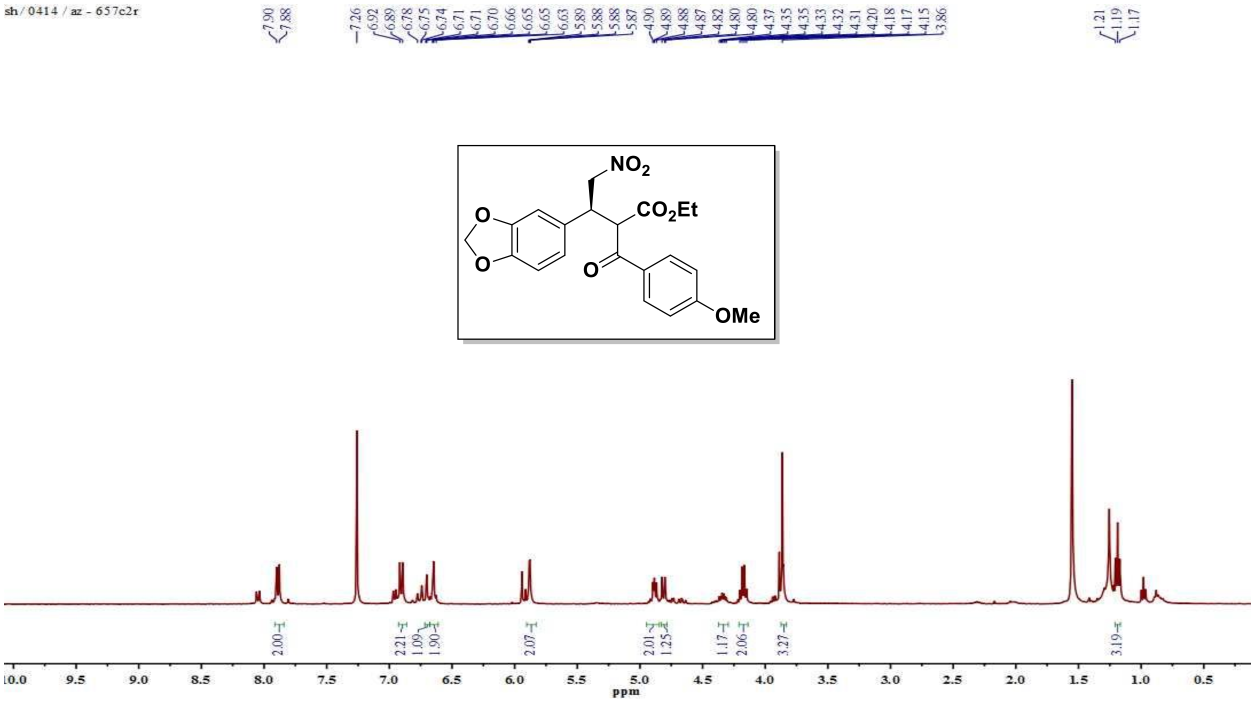

^{1}H NMR Spectrum of Compound **2o** (Major Diastereoisomer) (400 MHz, $CDCl_3$)

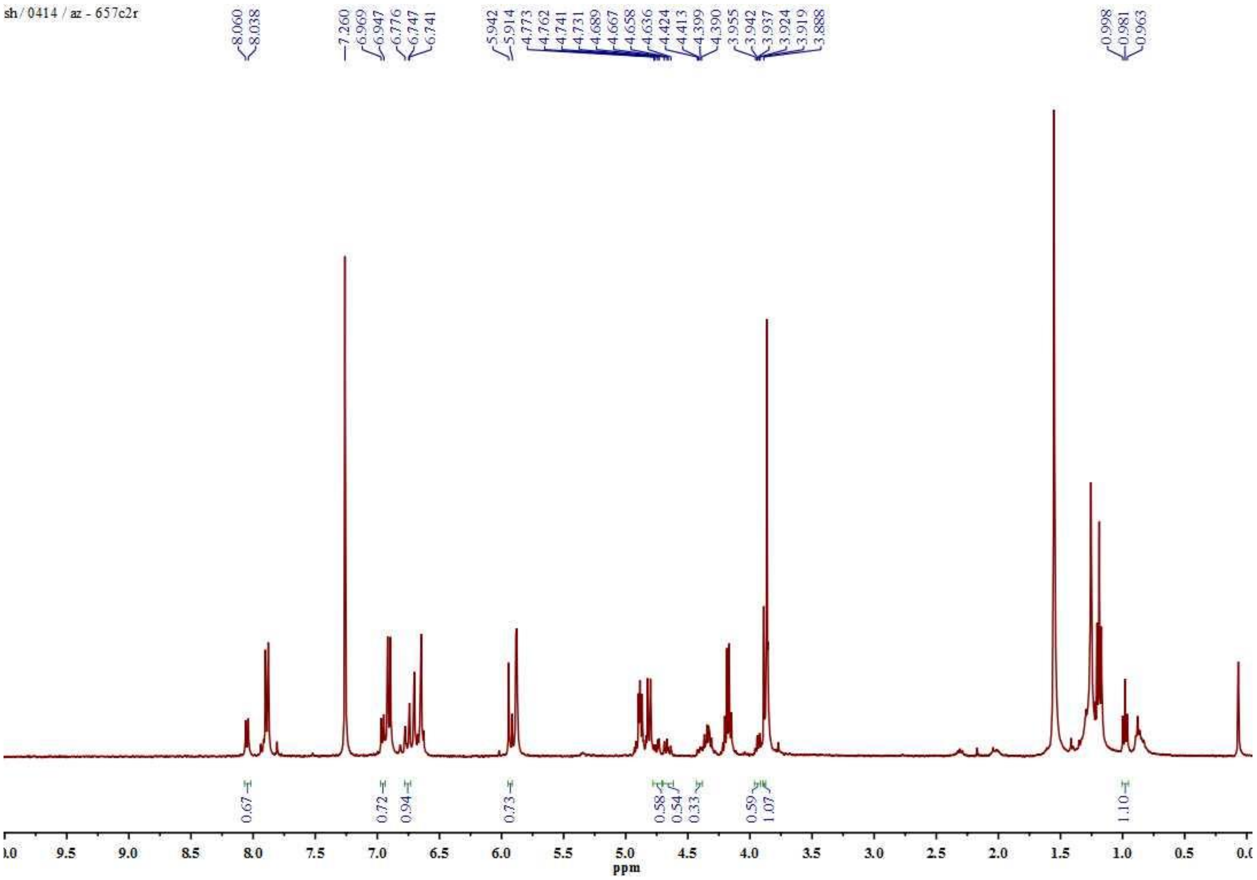

^{1}H NMR Spectrum of Compound **2o** (Minor Diastereoisomer) (400 MHz, $CDCl_3$)

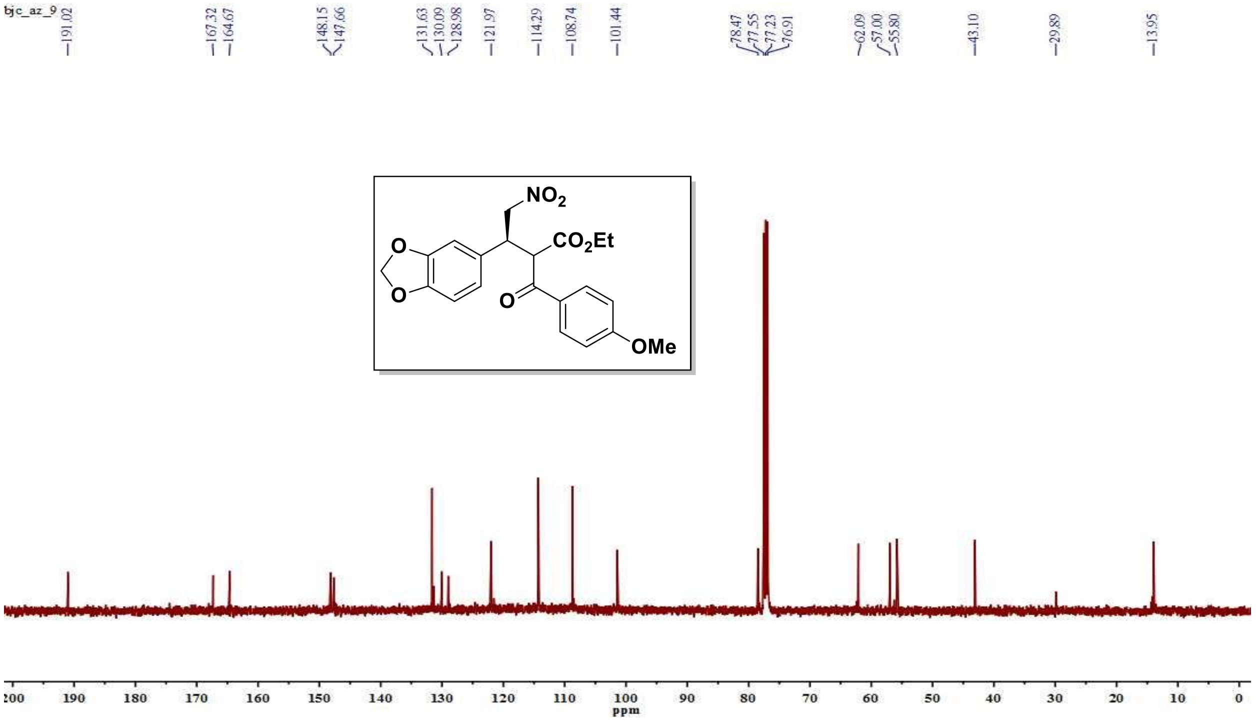

^{13}C NMR Spectrum of Compound **2o** (Major Diastereoisomer) (100 MHz, $CDCl_3$)

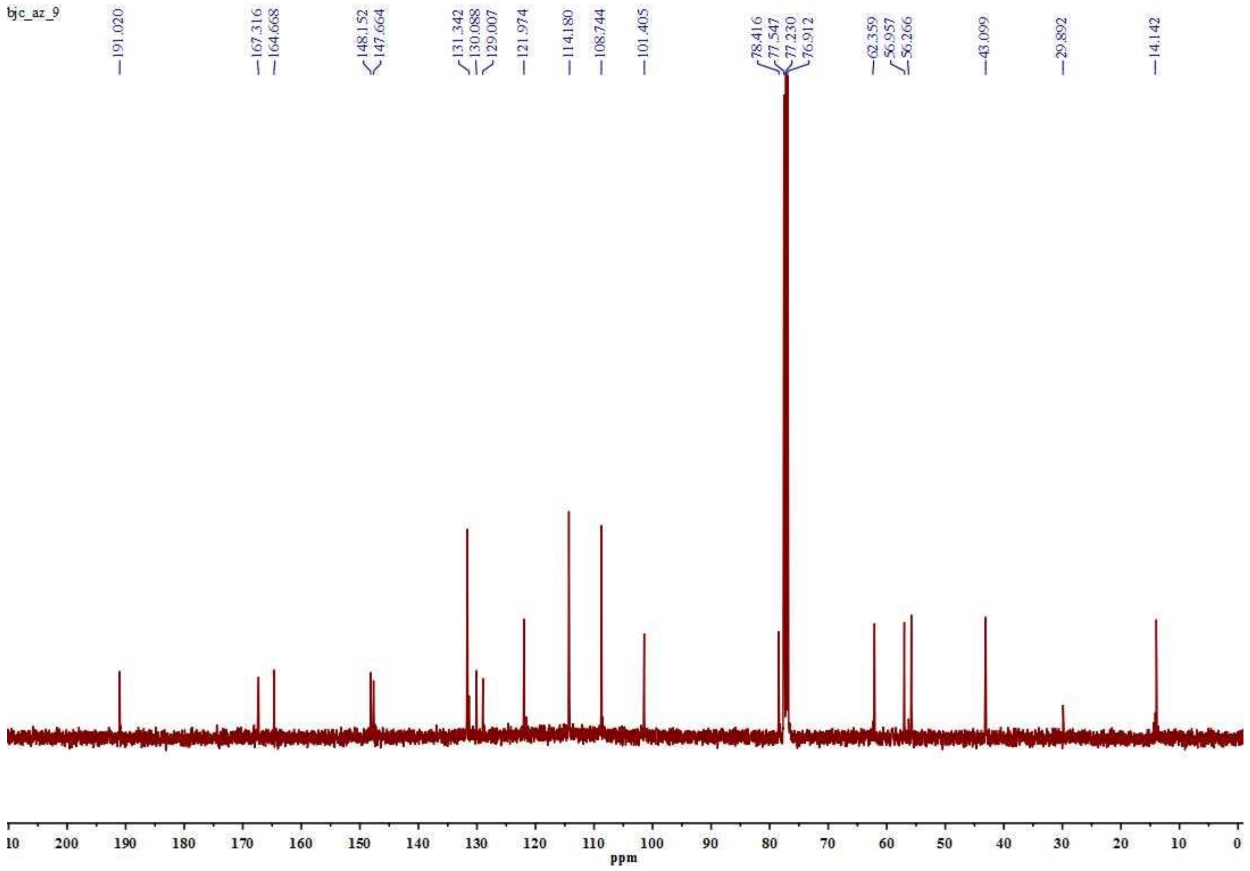

^{13}C NMR Spectrum of Compound **2o** (Minor Diastereoisomer) (100 MHz, $CDCl_3$)

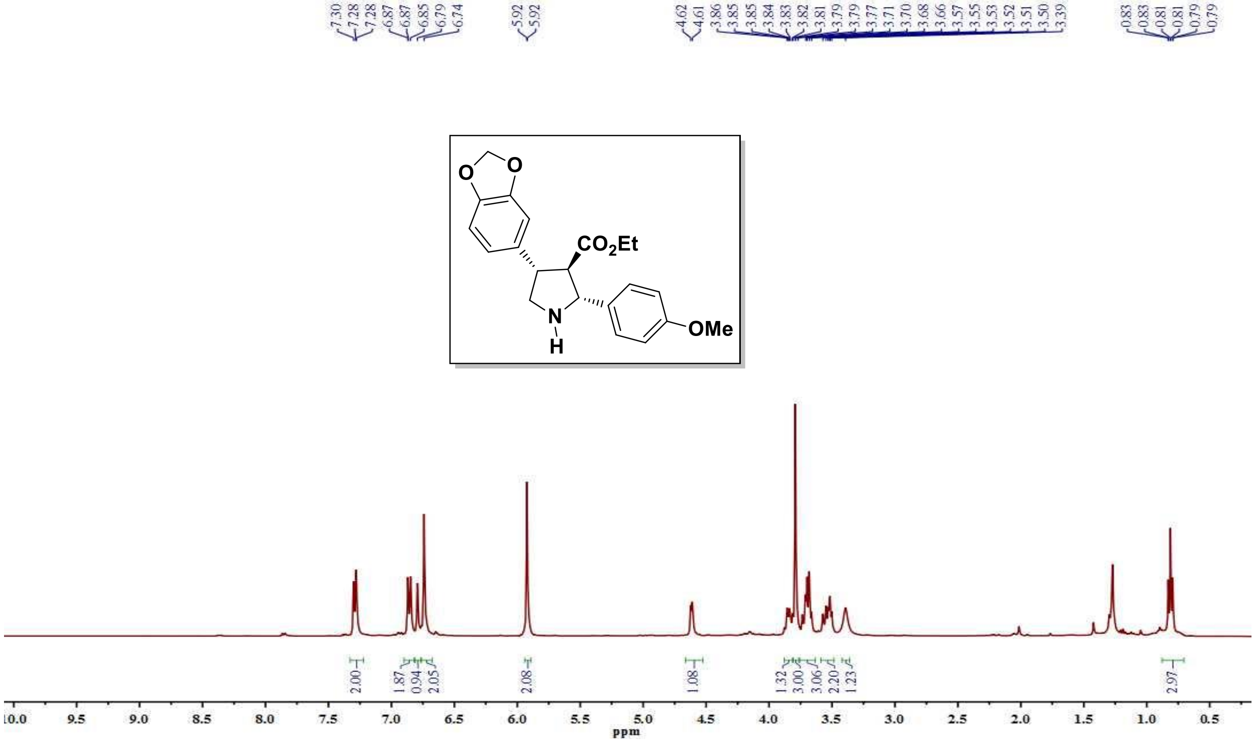

^{1}H NMR Spectrum of Compound **7** (400 MHz, $CDCl_3$)

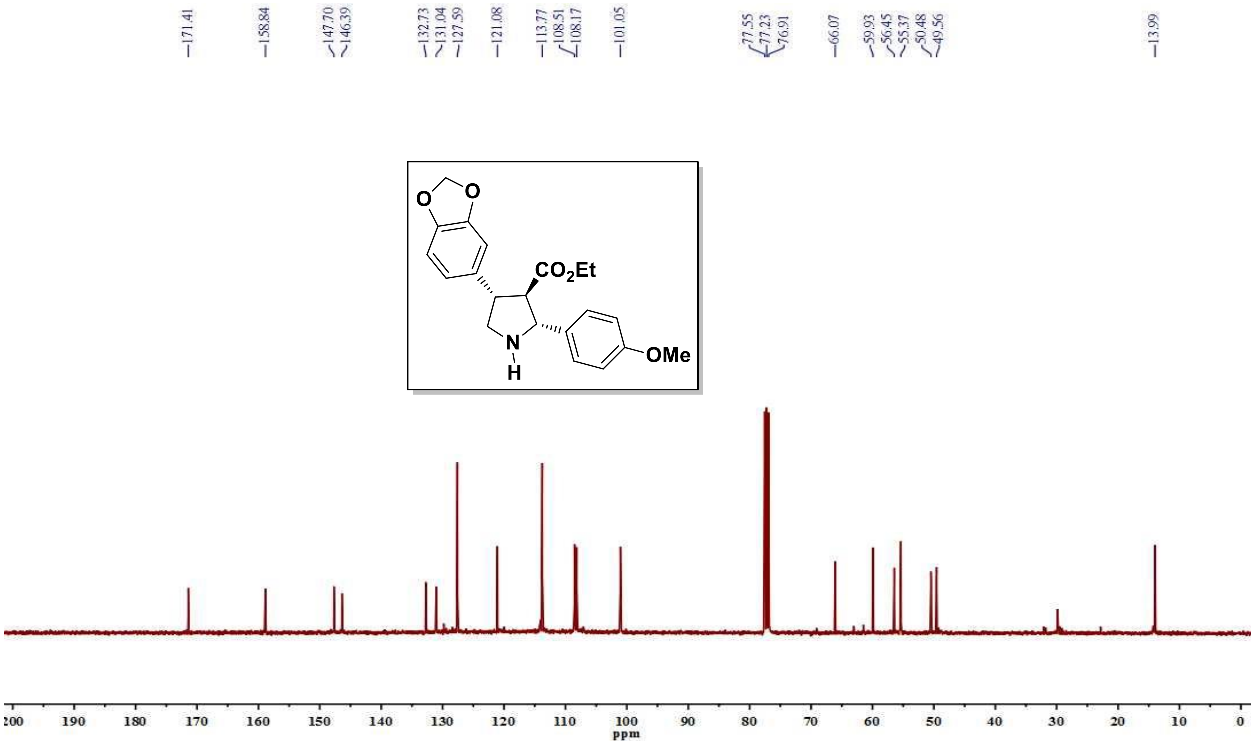

^{13}C NMR Spectrum of Compound **7** (100 MHz, $CDCl_3$)

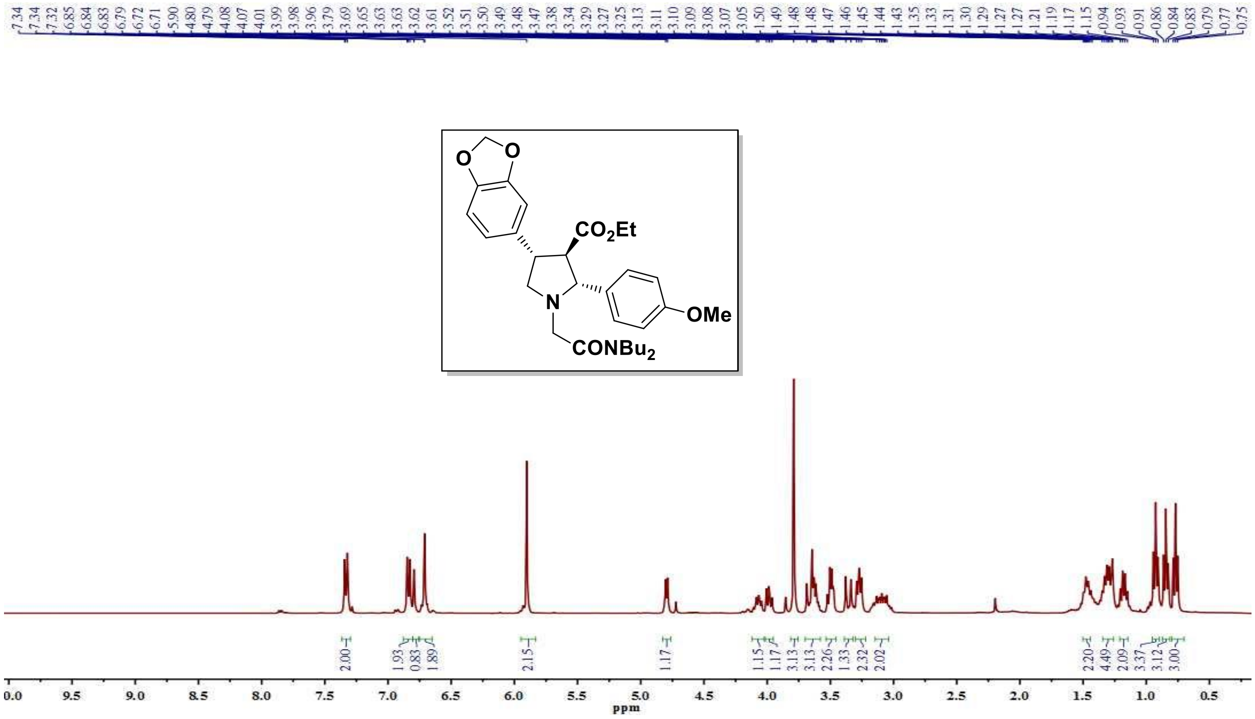

^{1}H NMR Spectrum of Compound **8** (400 MHz, $CDCl_3$)

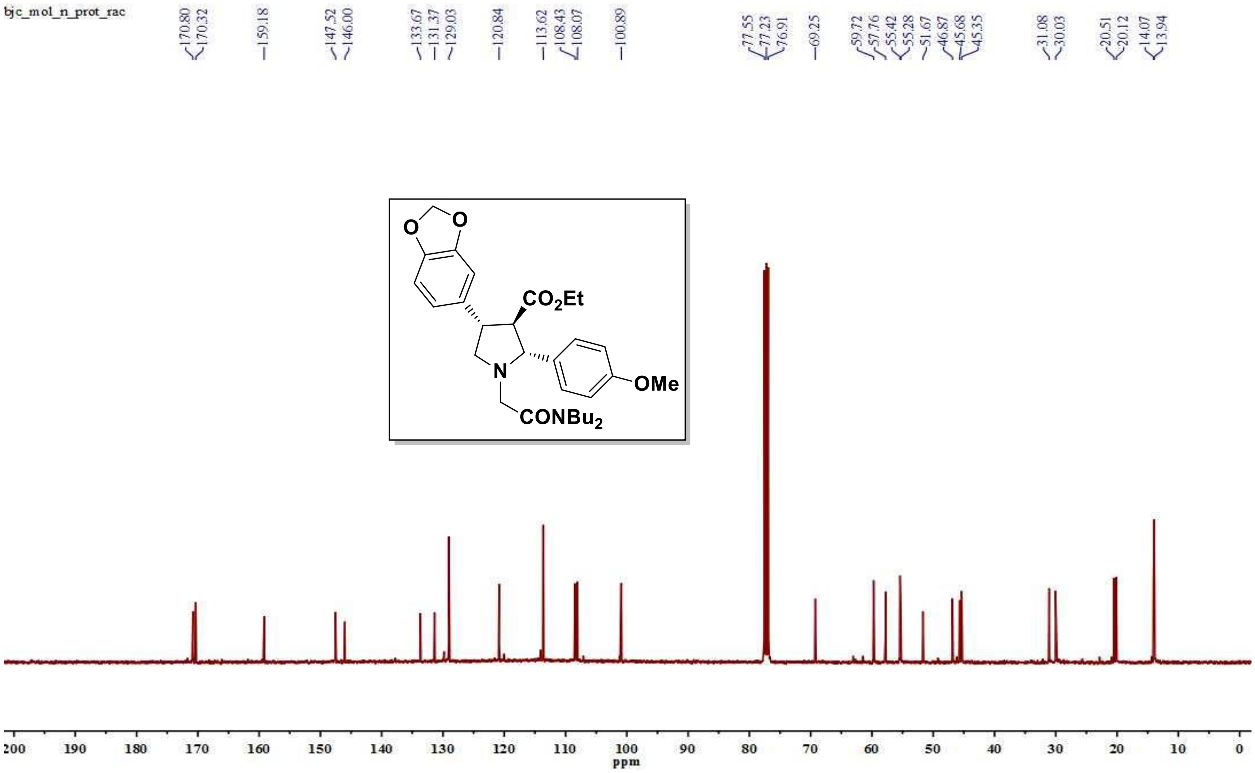

^{13}C NMR Spectrum of Compound **8** (100 MHz, $CDCl_3$)

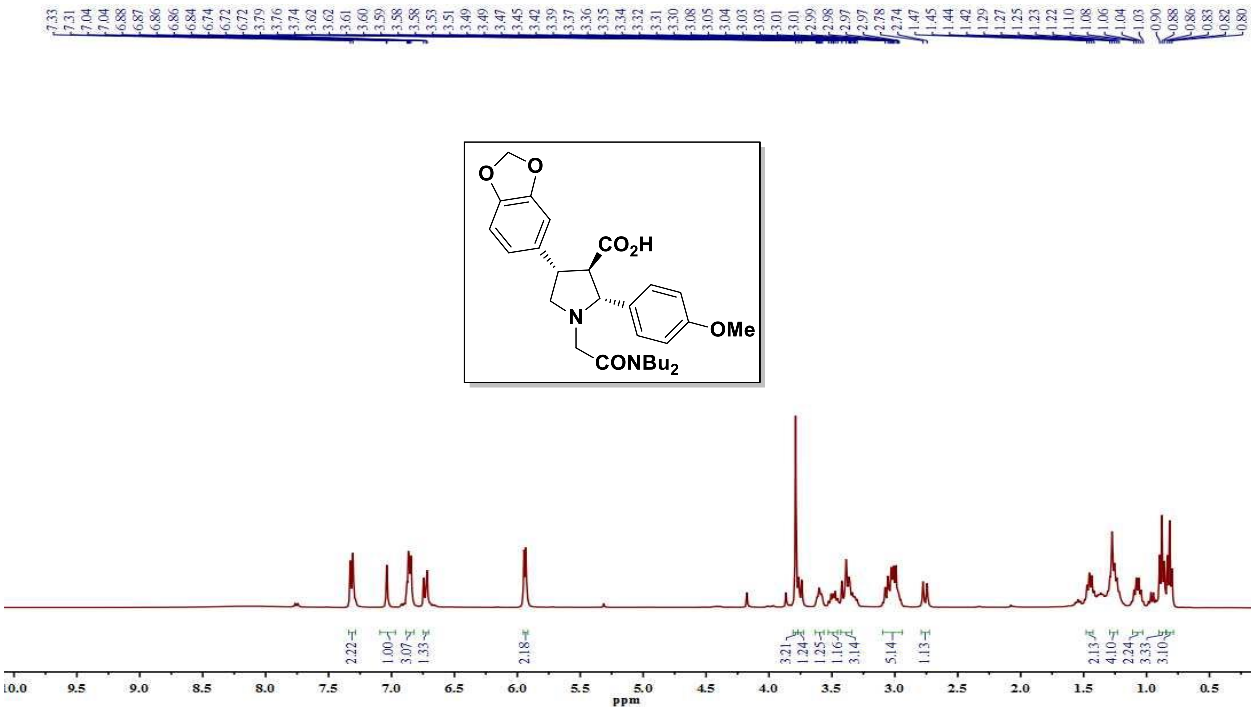

^{1}H NMR Spectrum of Compound **9 (ABT-627)** (400 MHz, $CDCl_3$)

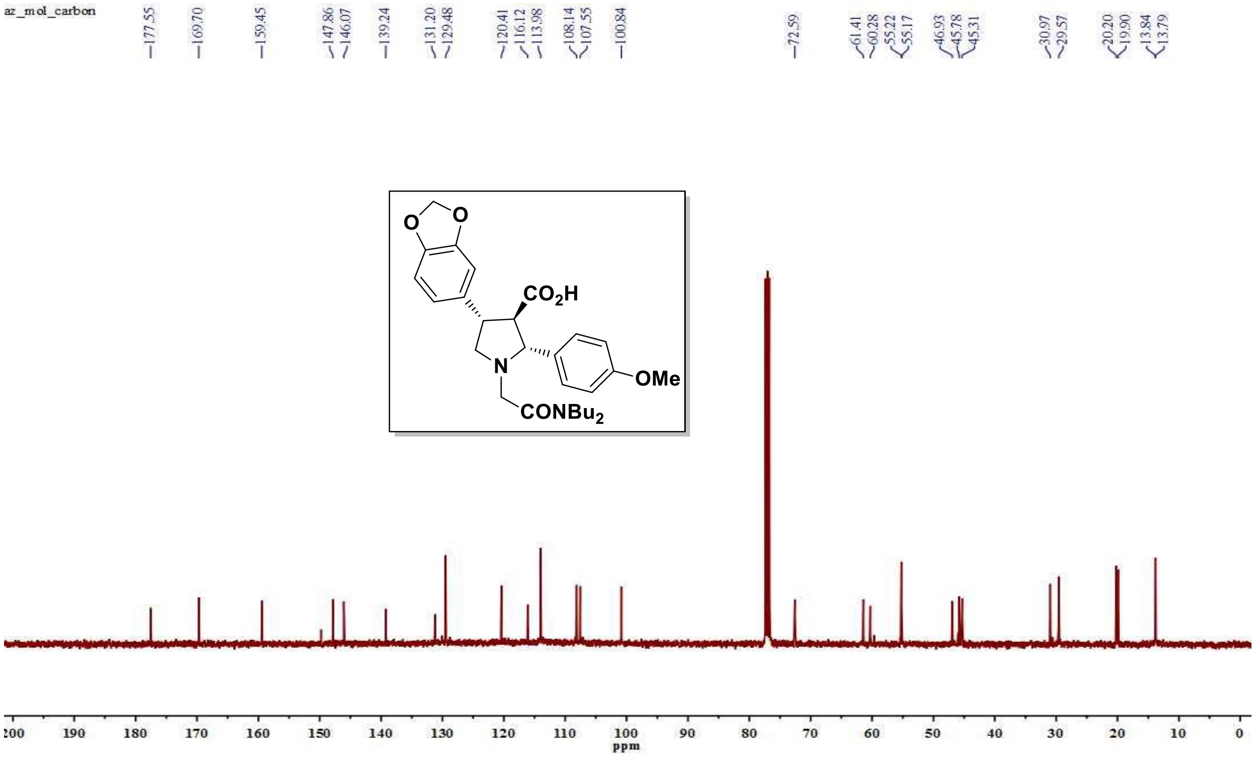

^{13}C NMR Spectrum of Compound **9 (ABT-627)** (100 MHz, $CDCl_3$)

HPLC Chromatogram

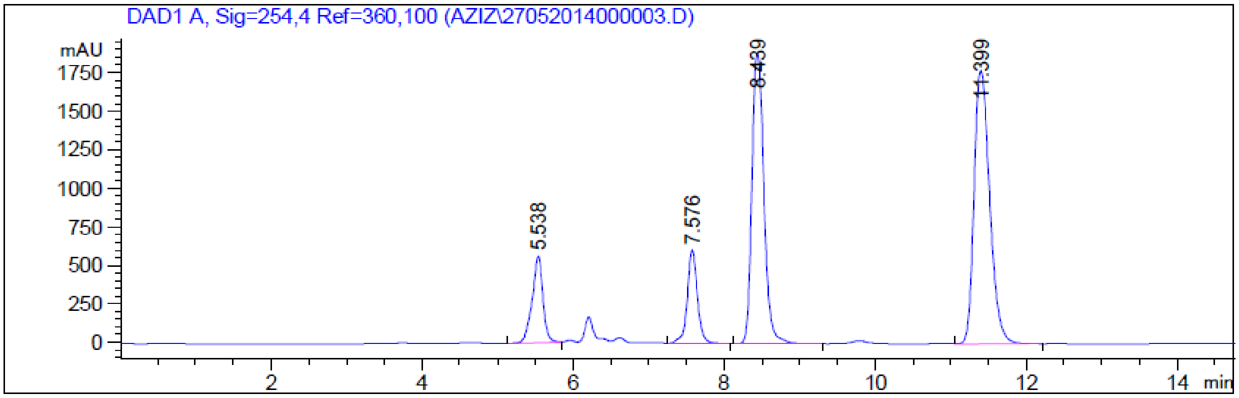

HPLC Chromatogram of Compound **2a** (catalyst **F**)

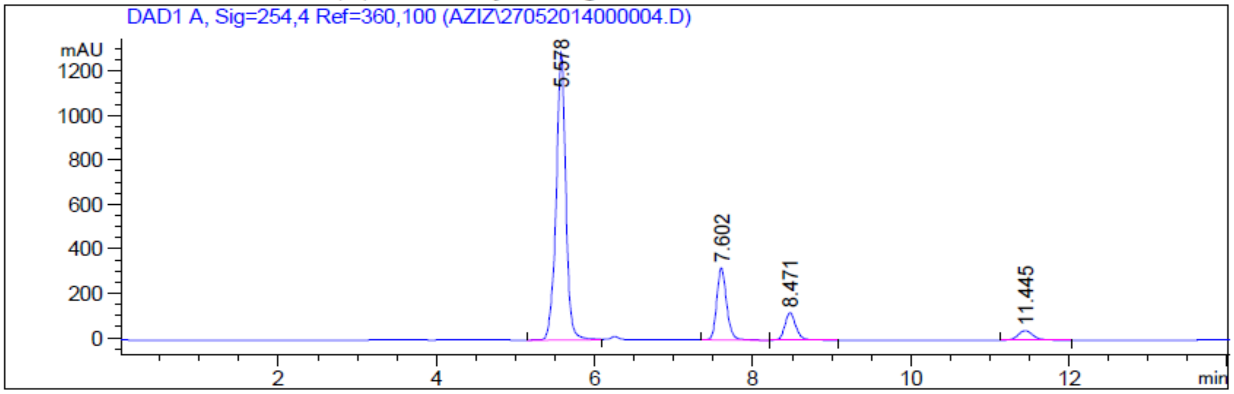

===

Area Percent Report

===

Sorted By : Signal
Multiplier : 1.0000
Dilution : 1.0000
Use Multiplier & Dilution Factor with ISTDs

Signal 1: DAD1 A, Sig=254,4 Ref=360,100

Peak #	RetTime [min]	Type	Width [min]	Area [mAU*s]	Height [mAU]	Area %
1	5.578	BV	0.1271	1.09664e4	1291.38562	70.6129
2	7.602	VB	0.1330	2814.12988	325.04605	18.1203
3	8.471	BB	0.1469	1189.12573	122.90531	7.6568
4	11.445	BB	0.1992	560.62067	43.25634	3.6099

HPLC Chromatogram of Compound **2a** (catalyst **H**)

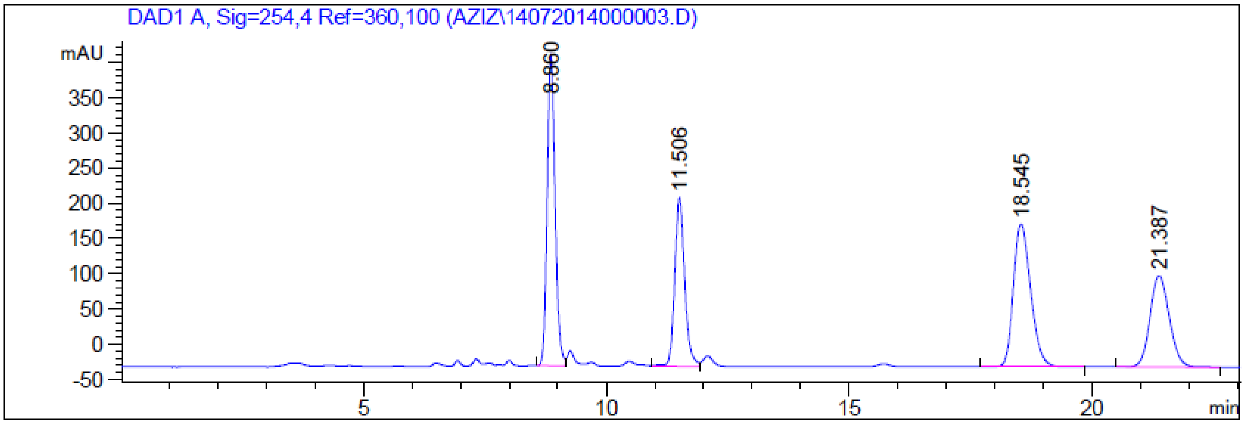

HPLC Chromatogram of Compound **2b** (catalyst **F**)

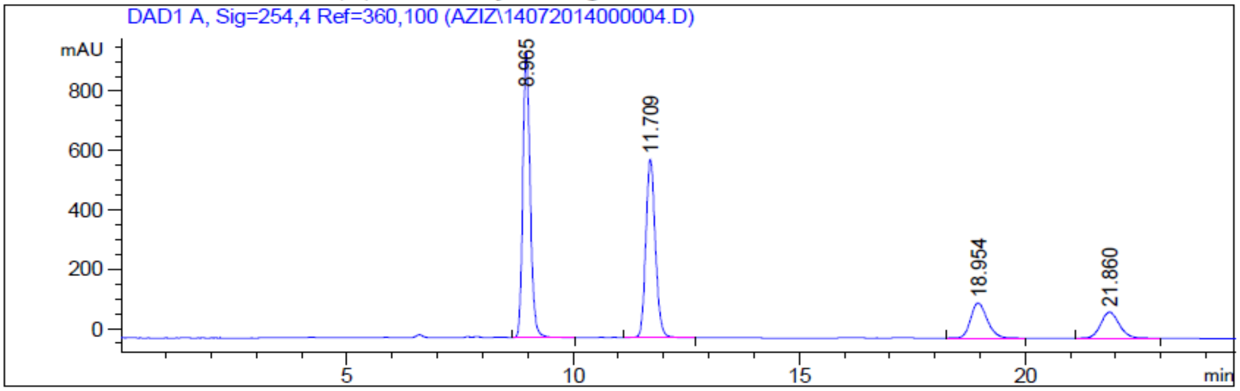

===

Area Percent Report

===

Sorted By : Signal
Multiplier : 1.0000
Dilution : 1.0000
Use Multiplier & Dilution Factor with ISTDs

Signal 1: DAD1 A, Sig=254,4 Ref=360,100

Peak #	RetTime [min]	Type	Width [min]	Area [mAU*s]	Height [mAU]	Area %
1	8.965	VB	0.1748	1.09495e4	961.63416	43.3251
2	11.709	BB	0.2257	8857.30371	601.97253	35.0467
3	18.954	BB	0.3936	2993.21436	118.13834	11.8436
4	21.860	BB	0.4395	2472.86450	87.56722	9.7847

HPLC Chromatogram of Compound **2b** (catalyst **H**)

Additional Info : Peak(s) manually integrated

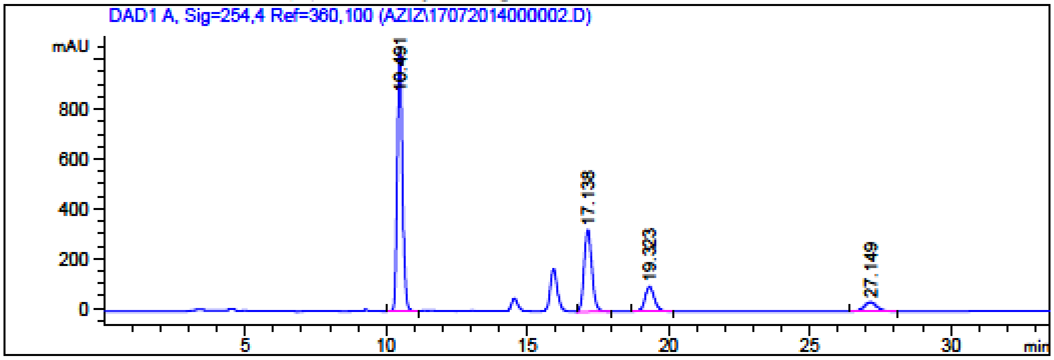

===

Area Percent Report

===

Sorted By : Signal
Multiplier : 1.0000
Dilution : 1.0000
Use Multiplier & Dilution Factor with ISTDs

Signal 1: DAD1 A, Sig=254,4 Ref=360,100

Peak #	RetTime [min]	Type	Width [min]	Area [mAU*s]	Height [mAU]	Area %
1	10.491	BB	0.1978	1.34707e4	1049.05823	58.4579
2	17.138	BB	0.2966	6259.89355	328.07233	27.1657
3	19.323	BB	0.3443	2210.84521	99.09370	9.5943
4	27.149	BB	0.4696	1101.96289	35.95162	4.7821

HPLC Chromatogram of Compound **2c** (catalyst **F**)

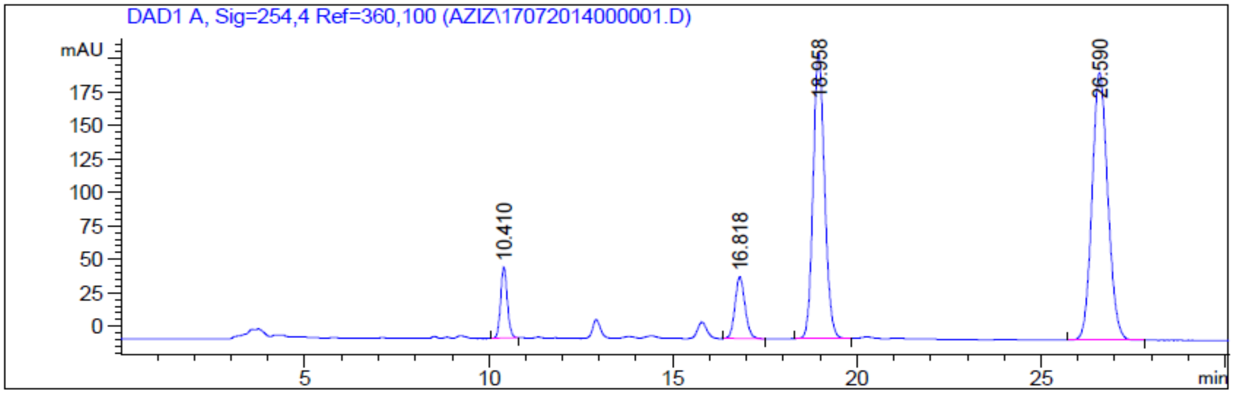

==

Area Percent Report

==

Sorted By : Signal
Multiplier : 1.0000
Dilution : 1.0000
Use Multiplier & Dilution Factor with ISTDs

Signal 1: DAD1 A, Sig=254,4 Ref=360,100

Peak #	RetTime [min]	Type	Width [min]	Area [mAU*s]	Height [mAU]	Area %
1	10.410	BB	0.1957	672.51135	53.13678	5.5600
2	16.818	BB	0.2886	870.13617	46.01580	7.1938
3	18.958	BB	0.3341	4607.40625	213.23586	38.0915
4	26.590	BB	0.4594	5945.58057	199.64818	49.1548

HPLC Chromatogram of Compound **2c** (catalyst **H**)

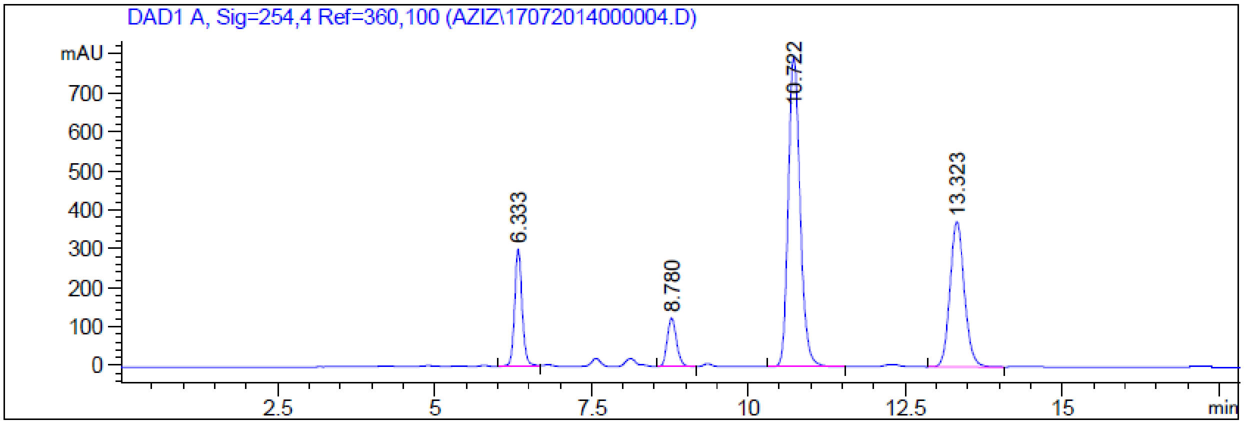

HPLC Chromatogram of Compound **2d** (catalyst **F**)

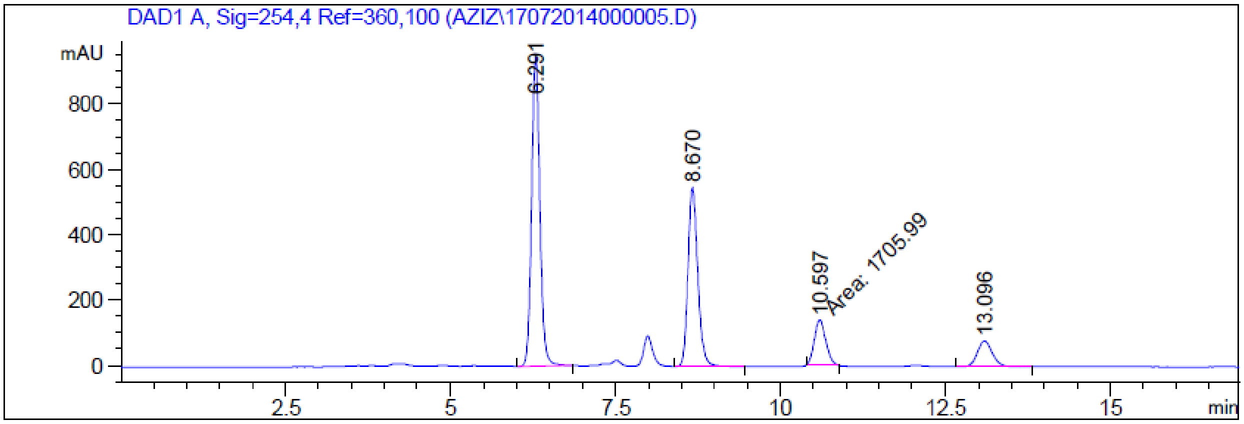


```
=====================================================================
                         Area Percent Report
=====================================================================

Sorted By                 :      Signal
Multiplier                :      1.0000
Dilution                  :      1.0000
Use Multiplier & Dilution Factor with ISTDs


Signal 1: DAD1 A, Sig=254,4 Ref=360,100
```

Peak #	RetTime [min]	Type	Width [min]	Area [mAU*s]	Height [mAU]	Area %
1	6.291	BB	0.1291	8047.50488	947.17084	48.2571
2	8.670	BB	0.1593	5670.91504	545.29535	34.0058
3	10.597	MM	0.2056	1705.99304	138.32527	10.2300
4	13.096	BB	0.2446	1251.89551	79.12386	7.5070

HPLC Chromatogram of Compound **2d** (catalyst **H**)

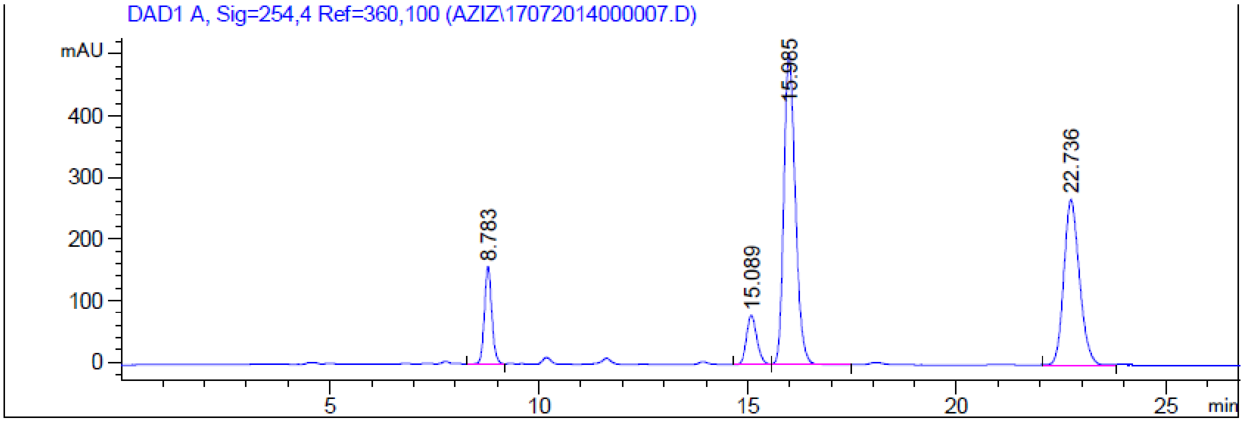

HPLC Chromatogram of Compound **2e** (catalyst **F**)

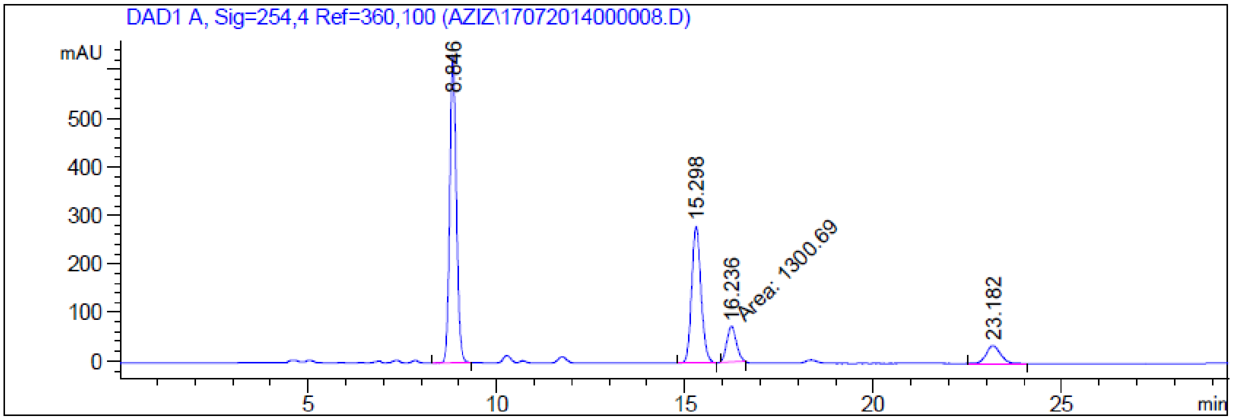

==

Area Percent Report

==

Sorted By : Signal
Multiplier : 1.0000
Dilution : 1.0000
Use Multiplier & Dilution Factor with ISTDs

Signal 1: DAD1 A, Sig=254,4 Ref=360,100

Peak #	RetTime [min]	Type	Width [min]	Area [mAU*s]	Height [mAU]	Area %
1	8.846	BB	0.1864	7602.93799	631.89838	51.3179
2	15.298	BV	0.2703	4946.63770	282.51614	33.3886
3	16.236	MM	0.2955	1300.69226	73.35119	8.7793
4	23.182	BB	0.4060	965.09387	36.79242	6.5141

HPLC Chromatogram of Compound **2e** (catalyst **H**)

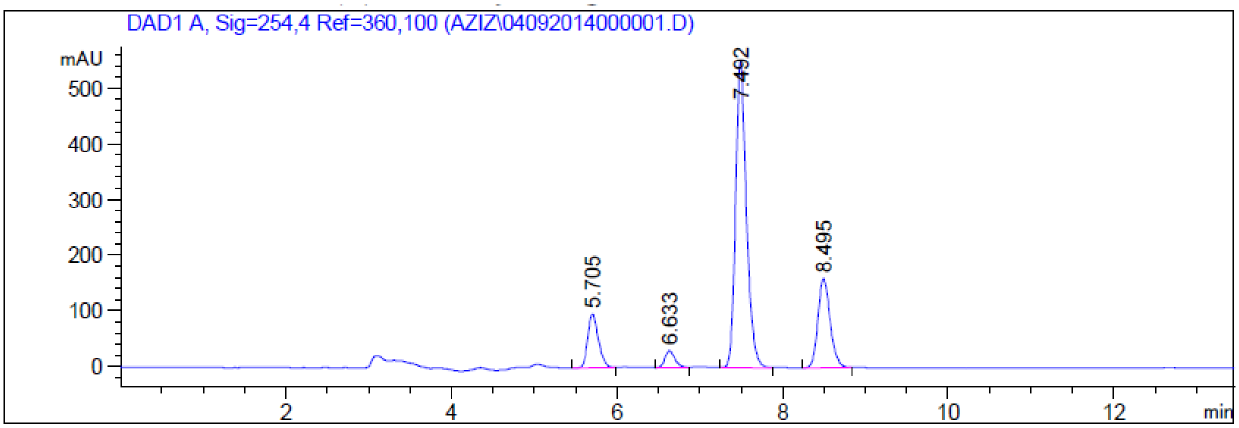

HPLC Chromatogram of Compound **2f** (catalyst **F**)

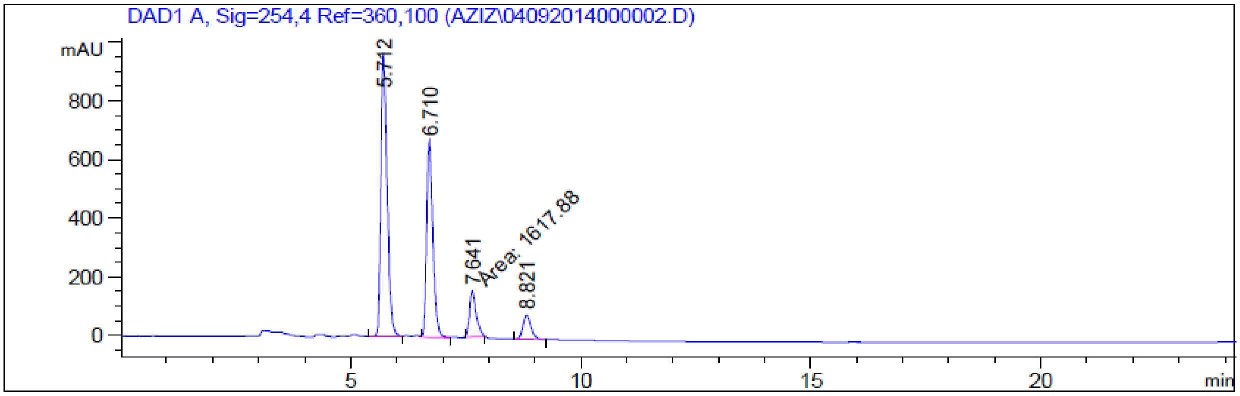


```
=====================================================================
                         Area Percent Report
=====================================================================

Sorted By             :      Signal
Multiplier            :      1.0000
Dilution              :      1.0000
Use Multiplier & Dilution Factor with ISTDs


Signal 1: DAD1 A, Sig=254,4 Ref=360,100
```

Peak #	RetTime [min]	Type	Width [min]	Area [mAU*s]	Height [mAU]	Area %
1	5.712	VB	0.1468	9366.77930	968.91626	51.2805
2	6.710	VV	0.1442	6306.74854	667.74414	34.5277
3	7.641	MM	0.1731	1617.88049	155.77136	8.8574
4	8.821	BB	0.1797	974.35840	82.57642	5.3343

HPLC Chromatogram of Compound **2f** (catalyst **H**)

Additional Info : Peak(s) manually integrated

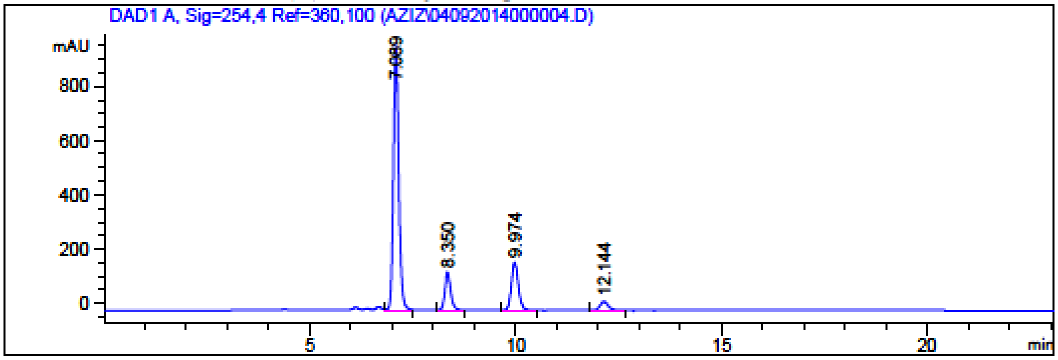

===

Area Percent Report

===

Sorted By : Signal
Multiplier : 1.0000
Dilution : 1.0000
Use Multiplier & Dilution Factor with ISTDs

Signal 1: DAD1 A, Sig=254,4 Ref=360,100

Peak #	RetTime [min]	Type	Width [min]	Area [mAU*s]	Height [mAU]	Area %
1	7.089	VB	0.1375	8706.03711	962.40027	68.7754
2	8.350	BV	0.1519	1422.05957	140.69531	11.2339
3	9.974	BB	0.1785	2078.02295	177.58360	16.4158
4	12.144	BB	0.2121	452.52609	32.56121	3.5748

HPLC Chromatogram of Compound **2g** (catalyst **F**)

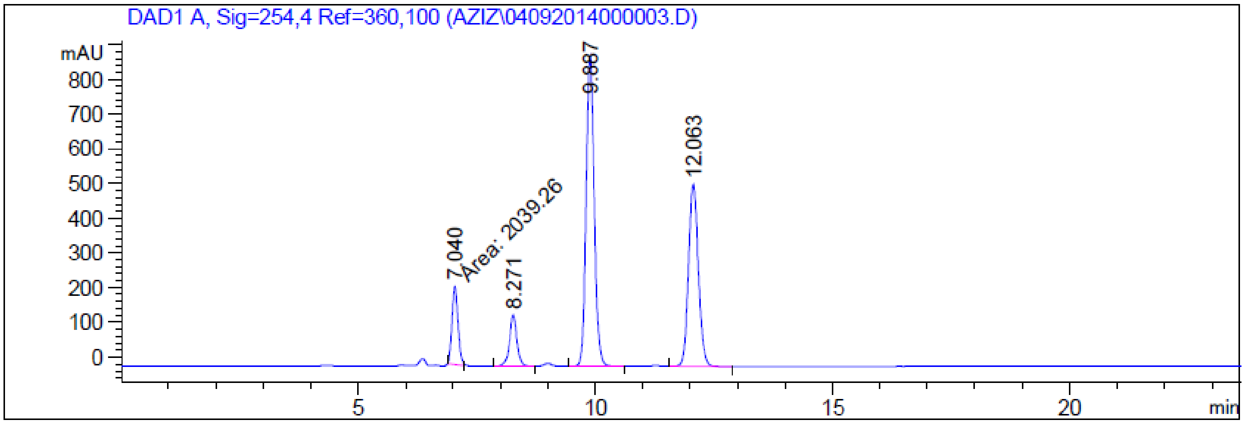

==

Area Percent Report

==

Sorted By : Signal
Multiplier : 1.0000
Dilution : 1.0000
Use Multiplier & Dilution Factor with ISTDs

Signal 1: DAD1 A, Sig=254,4 Ref=360,100

Peak #	RetTime [min]	Type	Width [min]	Area [mAU*s]	Height [mAU]	Area %
1	7.040	MM	0.1501	2039.25610	226.44124	9.3439
2	8.271	BB	0.1679	1634.19470	146.76547	7.4879
3	9.887	BB	0.1836	1.06843e4	892.76276	48.9553
4	12.063	BB	0.2184	7466.78711	523.46533	34.2129

HPLC Chromatogram of Compound **2g** (catalyst **H**)

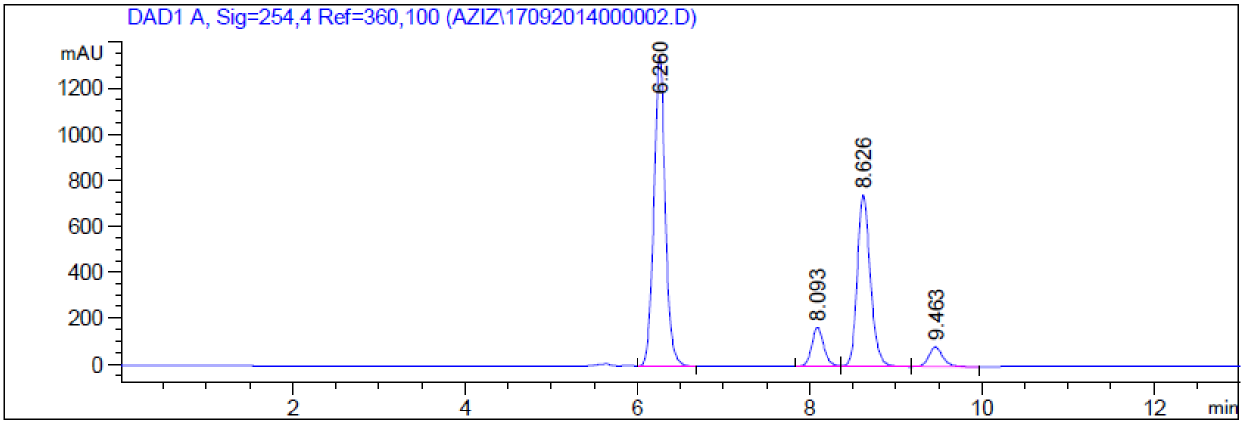

HPLC Chromatogram of Compound **2h** (catalyst **F**)

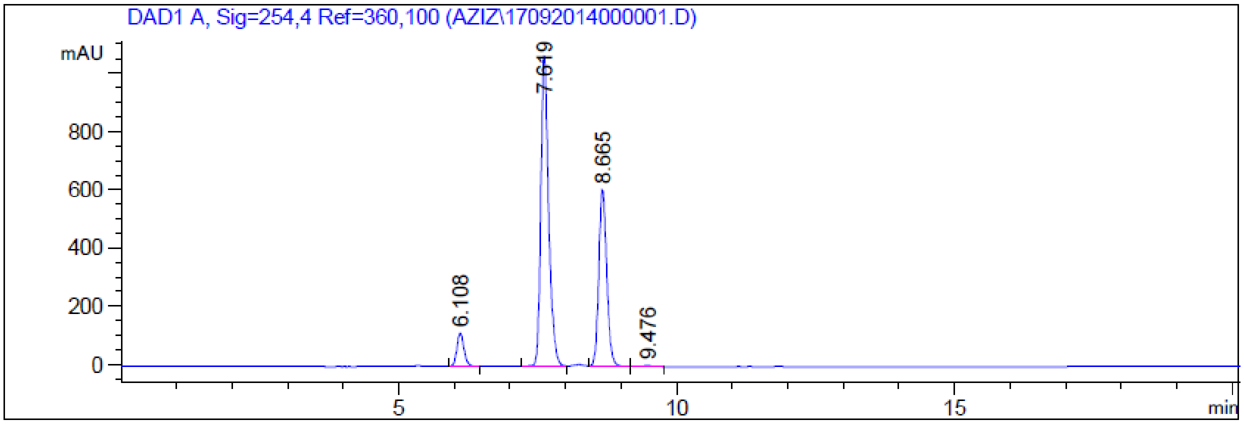


```
==================================================================
                       Area Percent Report
==================================================================

Sorted By             :      Signal
Multiplier            :      1.0000
Dilution              :      1.0000
Use Multiplier & Dilution Factor with ISTDs


Signal 1: DAD1 A, Sig=254,4 Ref=360,100
```

Peak #	RetTime [min]	Type	Width [min]	Area [mAU*s]	Height [mAU]	Area %
1	6.108	BB	0.1309	990.18909	114.54664	5.7304
2	7.619	BV	0.1443	1.02623e4	1065.87573	59.3903
3	8.665	VB	0.1513	5996.02197	606.57910	34.7003
4	9.476	BB	0.1761	30.92213	2.65132	0.1790

HPLC Chromatogram of Compound **2h** (catalyst **H**)

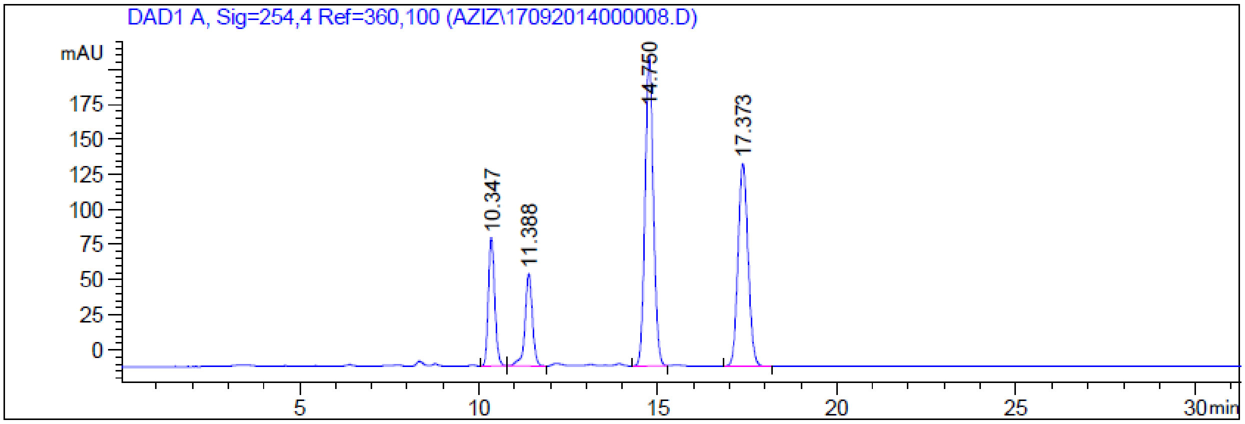

HPLC Chromatogram of Compound **2i** (catalyst **F**)

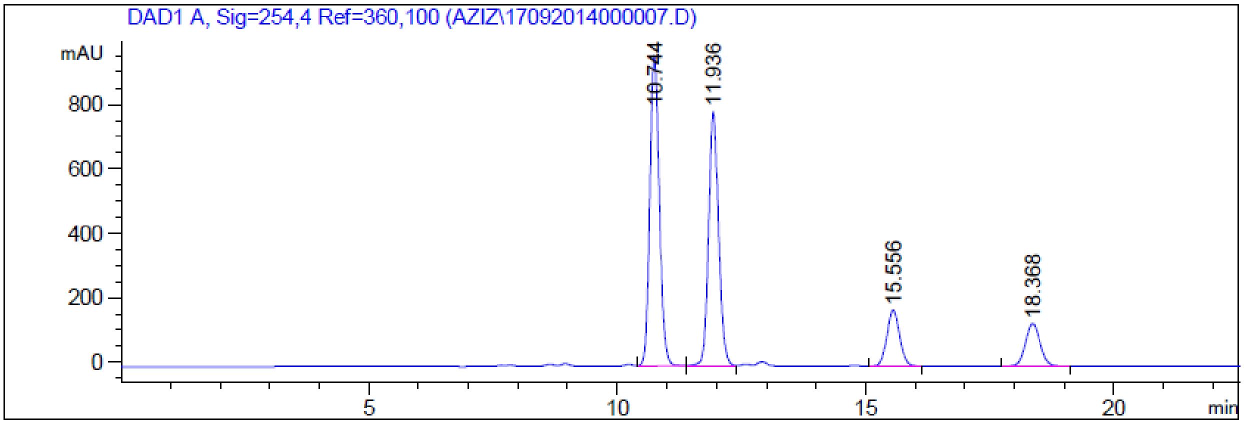


```
==========================================================================
                           Area Percent Report
==========================================================================

Sorted By             :      Signal
Multiplier            :      1.0000
Dilution              :      1.0000
Use Multiplier & Dilution Factor with ISTDs


Signal 1: DAD1 A, Sig=254,4 Ref=360,100

Peak RetTime Type  Width     Area      Height     Area
  #   [min]        [min]   [mAU*s]     [mAU]        %
----|-------|----|-------|----------|----------|--------|
   1  10.744 VB    0.2086 1.28922e4  960.40515  43.1507
   2  11.936 BB    0.2205 1.12272e4  786.91205  37.5780
   3  15.556 BB    0.2726 3071.67090  173.46368  10.2810
   4  18.368 BB    0.3151 2686.03979  131.06013   8.9903
```

HPLC Chromatogram of Compound **2i** (catalyst **H**)

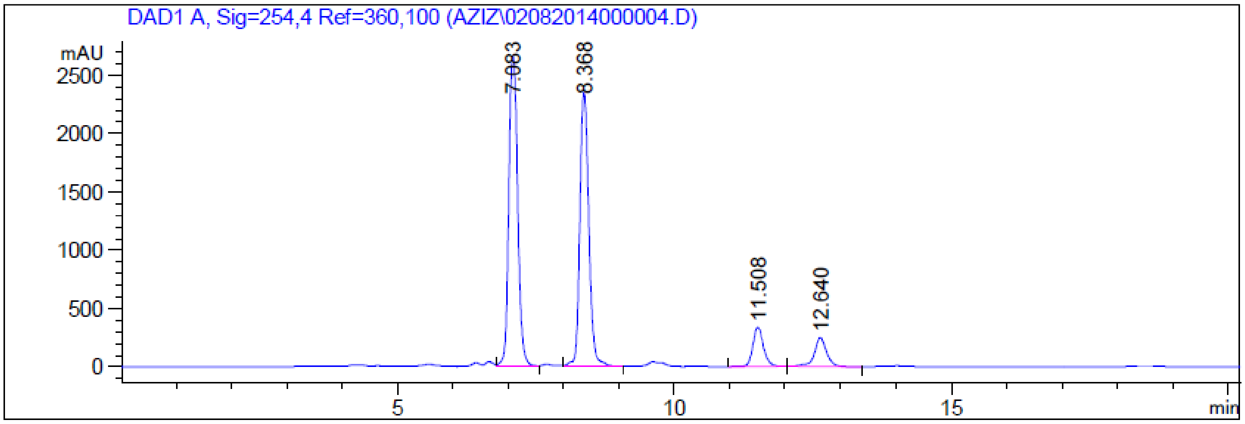

HPLC Chromatogram of Compound **2j** (catalyst **F**)

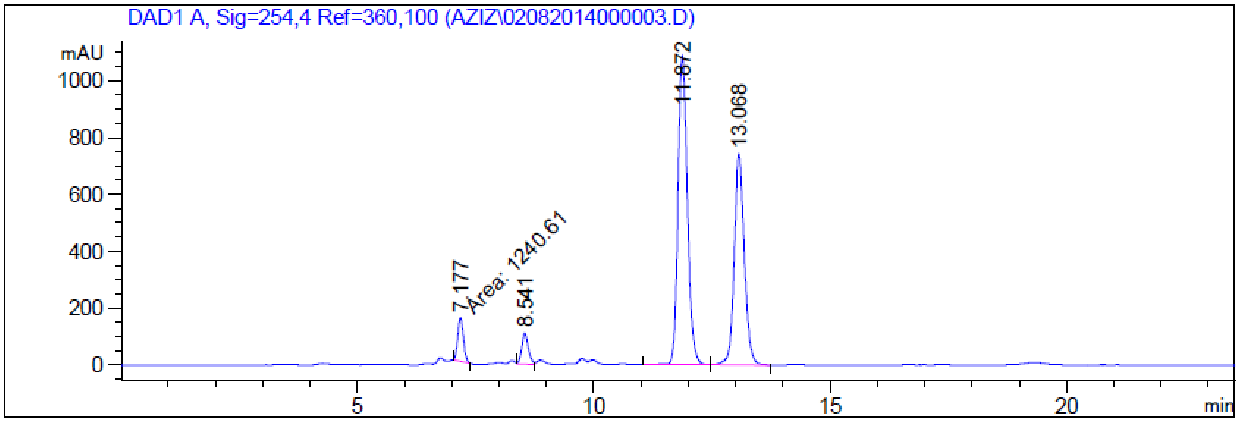

===

Area Percent Report

===

Sorted By : Signal
Multiplier : 1.0000
Dilution : 1.0000
Use Multiplier & Dilution Factor with ISTDs

Signal 1: DAD1 A, Sig=254,4 Ref=360,100

Peak #	RetTime [min]	Type	Width [min]	Area [mAU*s]	Height [mAU]	Area %
1	7.177	MM	0.1351	1240.61340	153.01846	4.4066
2	8.541	VV	0.1494	1048.92249	107.89597	3.7257
3	11.872	BB	0.2090	1.47802e4	1084.20447	52.4989
4	13.068	BB	0.2282	1.10836e4	742.25183	39.3687

HPLC Chromatogram of Compound **2j** (catalyst **H**)

Additional Info : Peak(s) manually integrated

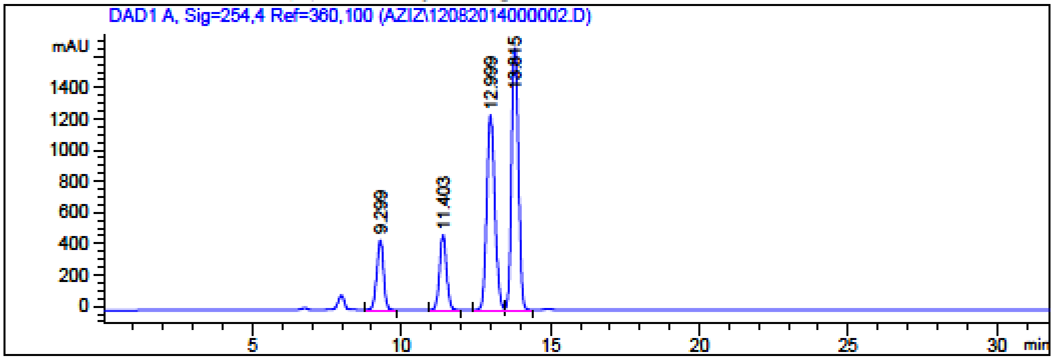

===

Area Percent Report

===

Sorted By : Signal
Multiplier : 1.0000
Dilution : 1.0000
Use Multiplier & Dilution Factor with ISTDs

Signal 1: DAD1 A, Sig=254,4 Ref=360,100

Peak #	RetTime [min]	Type	Width [min]	Area [mAU*s]	Height [mAU]	Area %
1	9.299	BB	0.2478	7143.62842	448.66660	10.8272
2	11.403	BB	0.2534	7899.78711	481.40689	11.9733
3	12.999	BV	0.2952	2.38442e4	1246.58984	36.1394
4	13.815	VB	0.2565	2.70909e4	1676.42993	41.0602

HPLC Chromatogram of Compound **2k** (catalyst **F**)

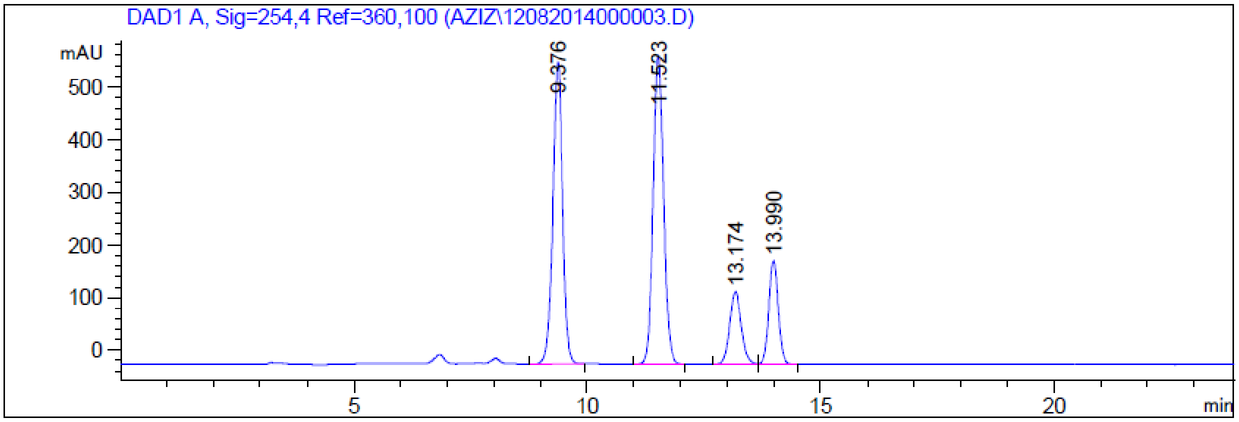


```
=====================================================================
                         Area Percent Report
=====================================================================

Sorted By             :      Signal
Multiplier            :      1.0000
Dilution              :      1.0000
Use Multiplier & Dilution Factor with ISTDs


Signal 1: DAD1 A, Sig=254,4 Ref=360,100
```

Peak #	RetTime [min]	Type	Width [min]	Area [mAU*s]	Height [mAU]	Area %
1	9.376	BB	0.2219	8352.37012	573.53320	36.9568
2	11.523	BB	0.2392	9100.32422	585.91217	40.2662
3	13.174	BV	0.2702	2409.10352	137.66310	10.6596
4	13.990	VB	0.2142	2738.58252	196.99352	12.1174

HPLC Chromatogram of Compound **2k** (catalyst **H**)

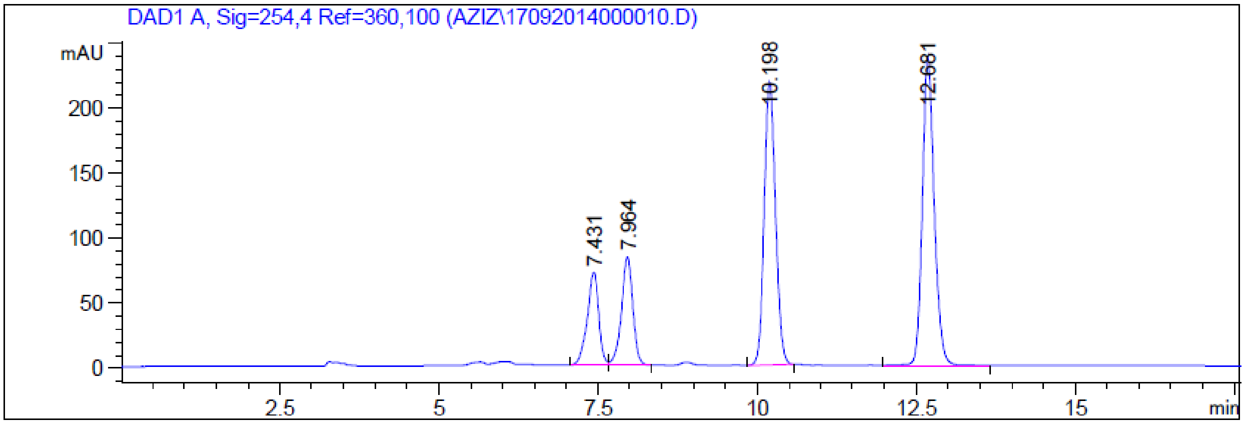

HPLC Chromatogram of Compound **2l** (catalyst **F**)

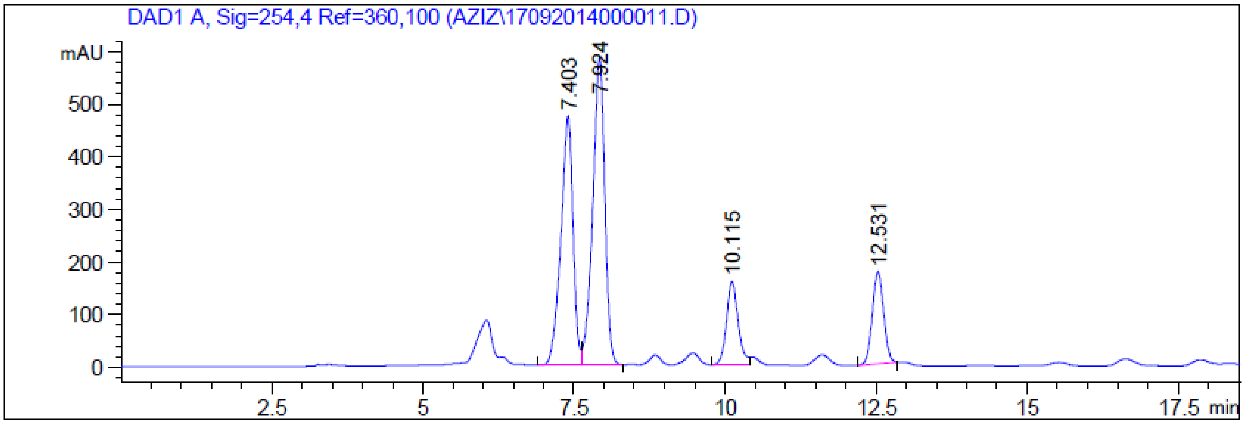

===

Area Percent Report

===

Sorted By : Signal
Multiplier : 1.0000
Dilution : 1.0000
Use Multiplier & Dilution Factor with ISTDs

Signal 1: DAD1 A, Sig=254,4 Ref=360,100

Peak #	RetTime [min]	Type	Width [min]	Area [mAU*s]	Height [mAU]	Area %
1	7.403	BV	0.2054	6618.39844	472.66504	34.7281
2	7.924	VV	0.2044	8061.20215	586.24323	42.2988
3	10.115	BV	0.2057	2140.74805	158.39182	11.2330
4	12.531	BB	0.1996	2237.39502	174.47870	11.7401

HPLC Chromatogram of Compound **2l** (catalyst **H**)

Additional Info : Peak(s) manually integrated

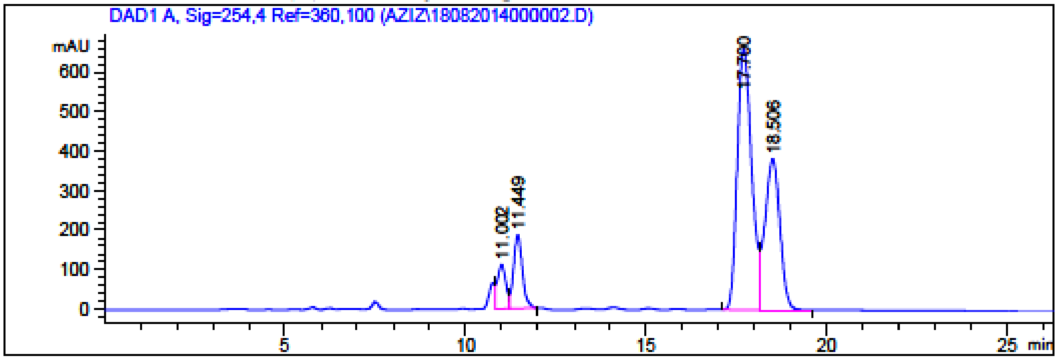

Area Percent Report

Sorted By : Signal
Multiplier : 1.0000
Dilution : 1.0000
Use Multiplier & Dilution Factor with ISTDs

Signal 1: DAD1 A, Sig=254,4 Ref=360,100

Peak #	RetTime [min]	Type	Width [min]	Area [mAU*s]	Height [mAU]	Area %
1	11.002	VV	0.2452	1834.40442	111.91914	5.3660
2	11.449	VB	0.2627	3203.34888	186.21306	9.3705
3	17.700	BV	0.4291	1.83078e4	660.98444	53.5543
4	18.506	VB	0.4303	1.08399e4	382.93246	31.7091

HPLC Chromatogram of Compound **2m** (catalyst **F**)

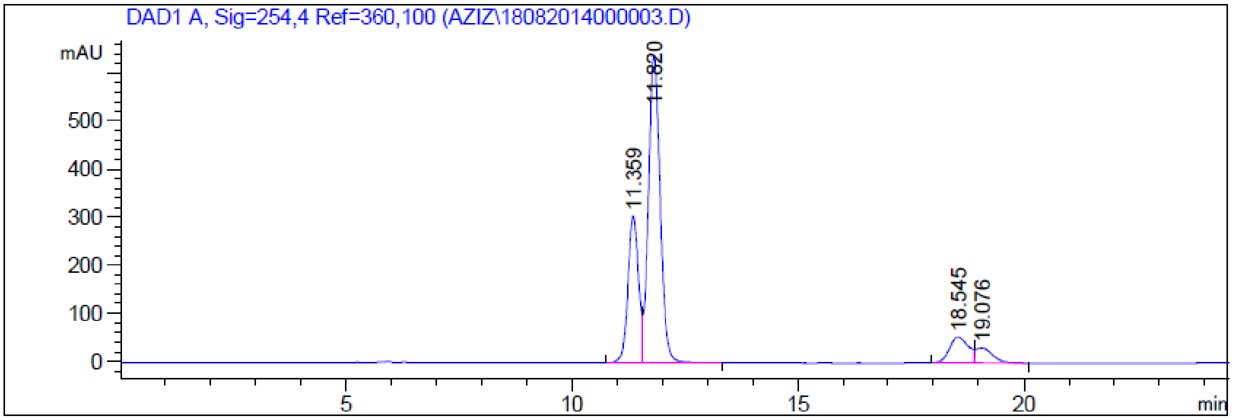

==

Area Percent Report

==

Sorted By : Signal
Multiplier : 1.0000
Dilution : 1.0000
Use Multiplier & Dilution Factor with ISTDs

Signal 1: DAD1 A, Sig=254,4 Ref=360,100

Peak #	RetTime [min]	Type	Width [min]	Area [mAU*s]	Height [mAU]	Area %
1	11.359	BV	0.2499	4980.87061	306.00101	26.4605
2	11.820	VB	0.2747	1.14358e4	639.37091	60.7519
3	18.545	BV	0.4485	1596.30933	53.75303	8.4803
4	19.076	VB	0.3913	810.80170	30.97958	4.3073

HPLC Chromatogram of Compound **2m** (catalyst **H**)

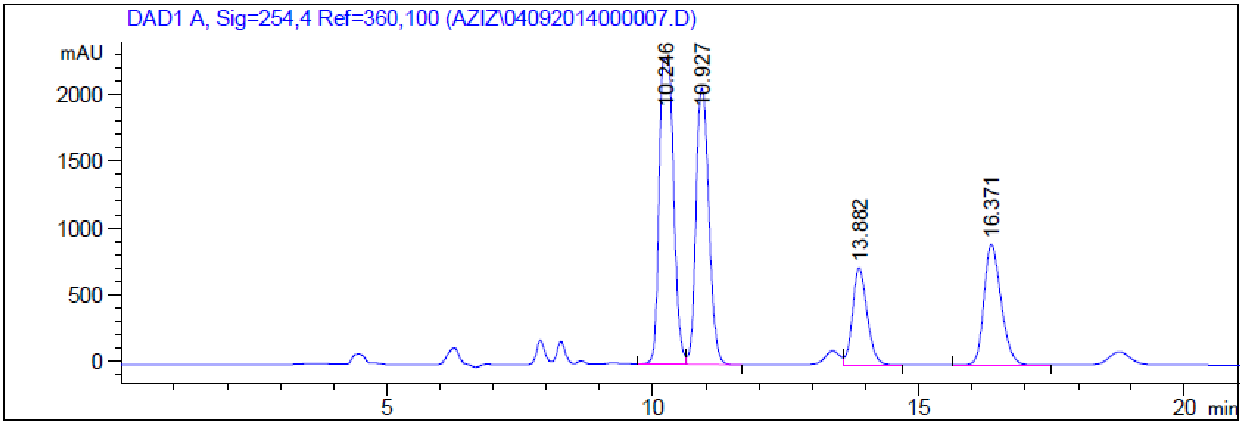

HPLC Chromatogram of Compound **2n** (catalyst **F**)

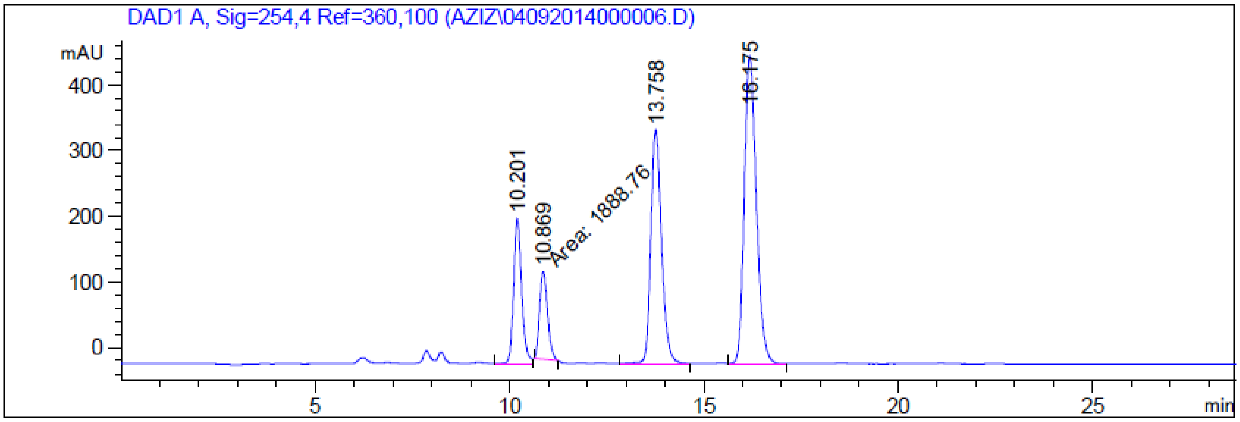

===
Area Percent Report
===

Sorted By : Signal
Multiplier : 1.0000
Dilution : 1.0000
Use Multiplier & Dilution Factor with ISTDs

Signal 1: DAD1 A, Sig=254,4 Ref=360,100

Peak #	RetTime [min]	Type	Width [min]	Area [mAU*s]	Height [mAU]	Area %
1	10.201	BV	0.2242	3219.21802	220.70094	14.7473
2	10.869	MM	0.2365	1888.75598	133.11102	8.6524
3	13.758	BB	0.2909	6735.30957	355.76675	30.8546
4	16.175	BB	0.3287	9985.94043	468.39224	45.7457

HPLC Chromatogram of Compound **2n** (catalyst **H**)

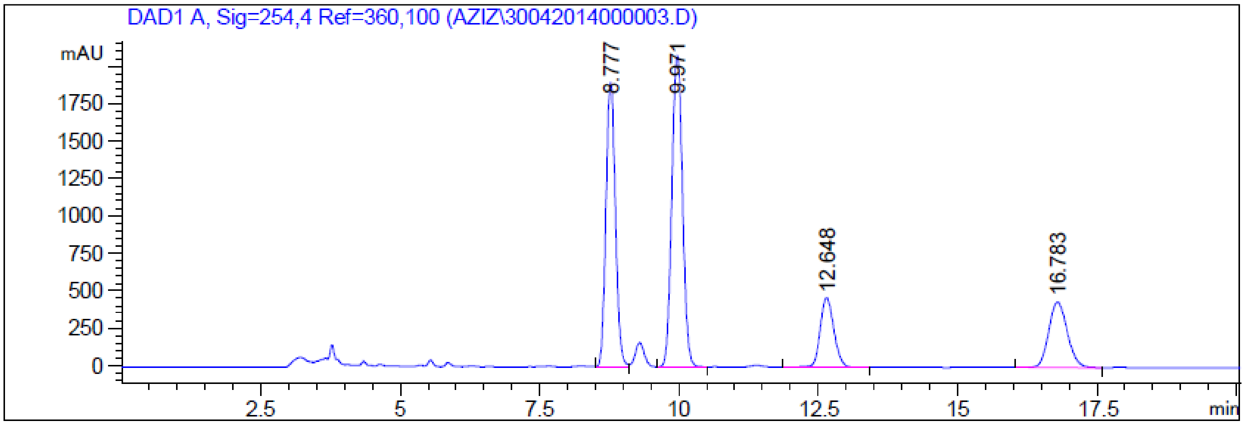

HPLC Chromatogram of Compound **2o** (catalyst **F**)

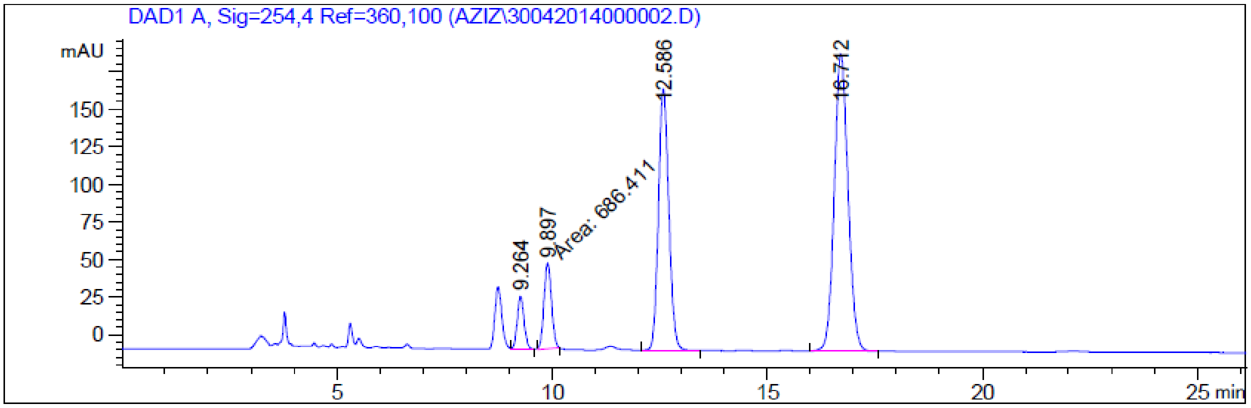


```
==================================================================
                     Area Percent Report
==================================================================

Sorted By             :      Signal
Multiplier            :      1.0000
Dilution              :      1.0000
Use Multiplier & Dilution Factor with ISTDs


Signal 1: DAD1 A, Sig=254,4 Ref=360,100

Peak RetTime Type  Width     Area      Height     Area
  #   [min]        [min]   [mAU*s]     [mAU]       %
----|-------|----|-------|----------|----------|--------|
   1   9.264 VV    0.1726  394.29639   35.22050   4.6862
   2   9.897 MM    0.2011  686.41125   56.90174   8.1580
   3  12.586 BB    0.2570 2870.93481  173.50891  34.1209
   4  16.712 BB    0.3542 4462.36670  197.05769  53.0350
```

HPLC Chromatogram of Compound **2o** (catalyst **H**)

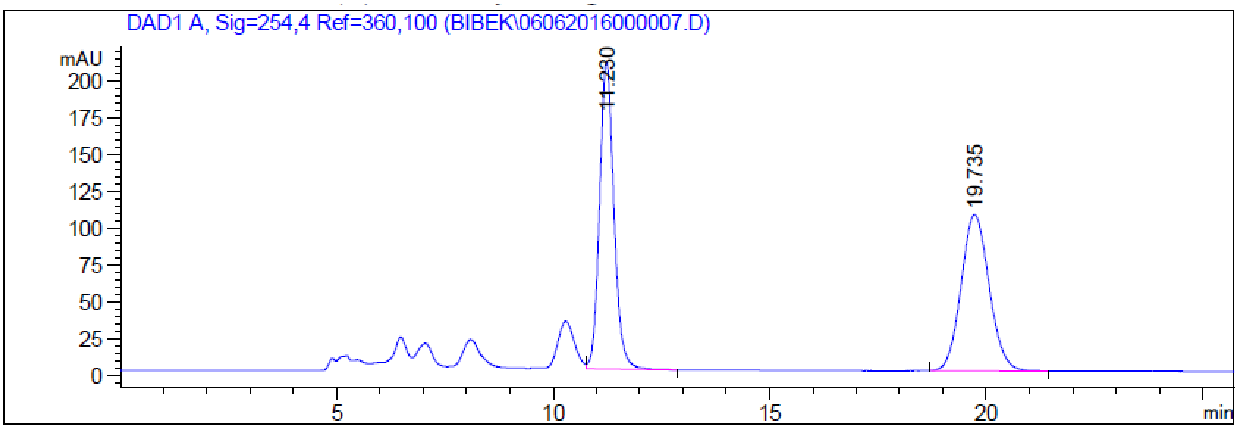

HPLC Chromatogram of Compound **(±) ABT-627**

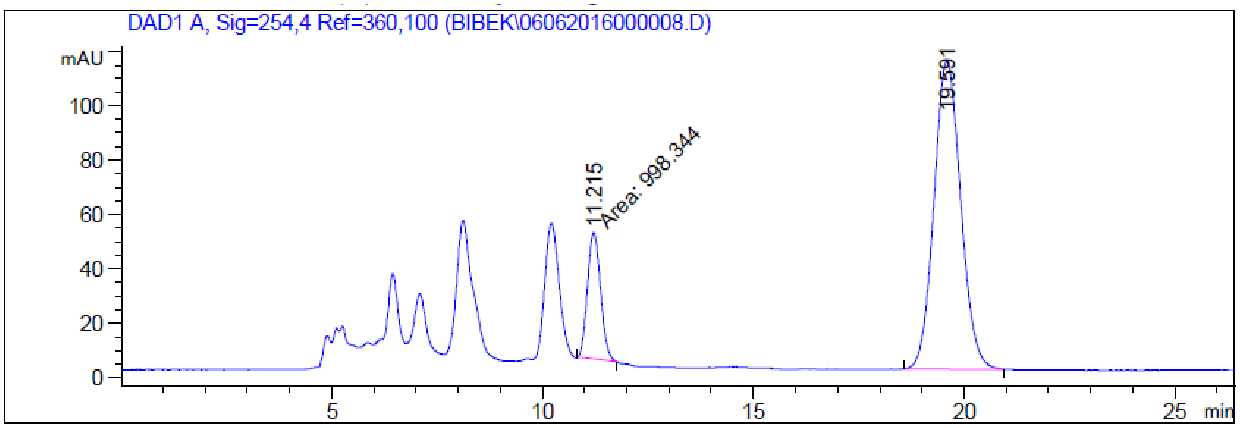


```
=====================================================================
                         Area Percent Report
=====================================================================

Sorted By             :      Signal
Multiplier            :      1.0000
Dilution              :      1.0000
Use Multiplier & Dilution Factor with ISTDs


Signal 1: DAD1 A, Sig=254,4 Ref=360,100

Peak RetTime Type  Width     Area      Height     Area
  #   [min]        [min]   [mAU*s]     [mAU]        %
----|-------|----|-------|----------|----------|--------|
   1  11.215 MM    0.3573  998.34399   46.56297  16.6861
   2  19.591 BB    0.6724 4984.74121  113.06143  83.3139
```

HPLC Chromatogram of Compound **ABT-627**
